# Nickel‐Catalyzed Arylative Cyclizations of Alkyne‐ and Allene‐Tethered Electrophiles using Arylboron Reagents

**DOI:** 10.1002/chem.202104230

**Published:** 2022-01-27

**Authors:** Simone M. Gillbard, Hon Wai Lam

**Affiliations:** ^1^ The GlaxoSmithKline Carbon Neutral Laboratories for Sustainable Chemistry University of Nottingham Jubilee Campus, Triumph Road NG7 2TU Nottingham UK; ^2^ School of Chemistry University of Nottingham University Park NG7 2RD Nottingham UK

**Keywords:** arylboron reagents, catalysis, cyclization, nickel, synthetic methods

## Abstract

The use of arylboron reagents in metal‐catalyzed domino addition–cyclization reactions is a well‐established strategy for the preparation of diverse, highly functionalized carbo‐ and heterocyclic products. Although rhodium‐ and palladium‐based catalysts have been commonly used for these reactions, more recent work has demonstrated nickel catalysis is also highly effective, in many cases offering unique reactivity and access to products that might otherwise not be readily available. This review gives an overview of nickel‐catalyzed arylative cyclizations of alkyne‐ and allene‐tethered electrophiles using arylboron reagents. The scope of the reactions is discussed in detail, and general mechanistic concepts underpinning the processes are described.

## Graphical abstract

**The use of arylboron reagents** in metal‐catalyzed domino addition‐cyclization reactions is a well‐established strategy for the preparation of diverse carbo‐ and heterocyclic products. Recent studies have demonstrated nickel catalysis is highly effective in these reactions, in many cases offering unique reactivity and access to products that might otherwise not be readily available using other metal catalysts. This review gives an overview of nickel‐catalyzed arylative cyclizations of alkyne‐ and allene‐tethered electrophiles using arylboron reagents.

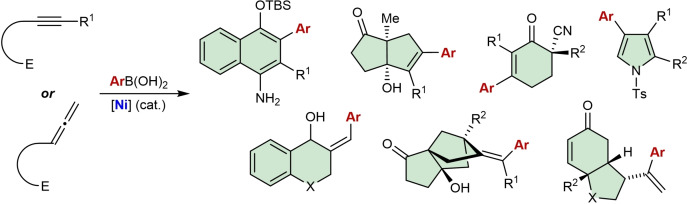

## 1 Introduction

**Scheme 1:**
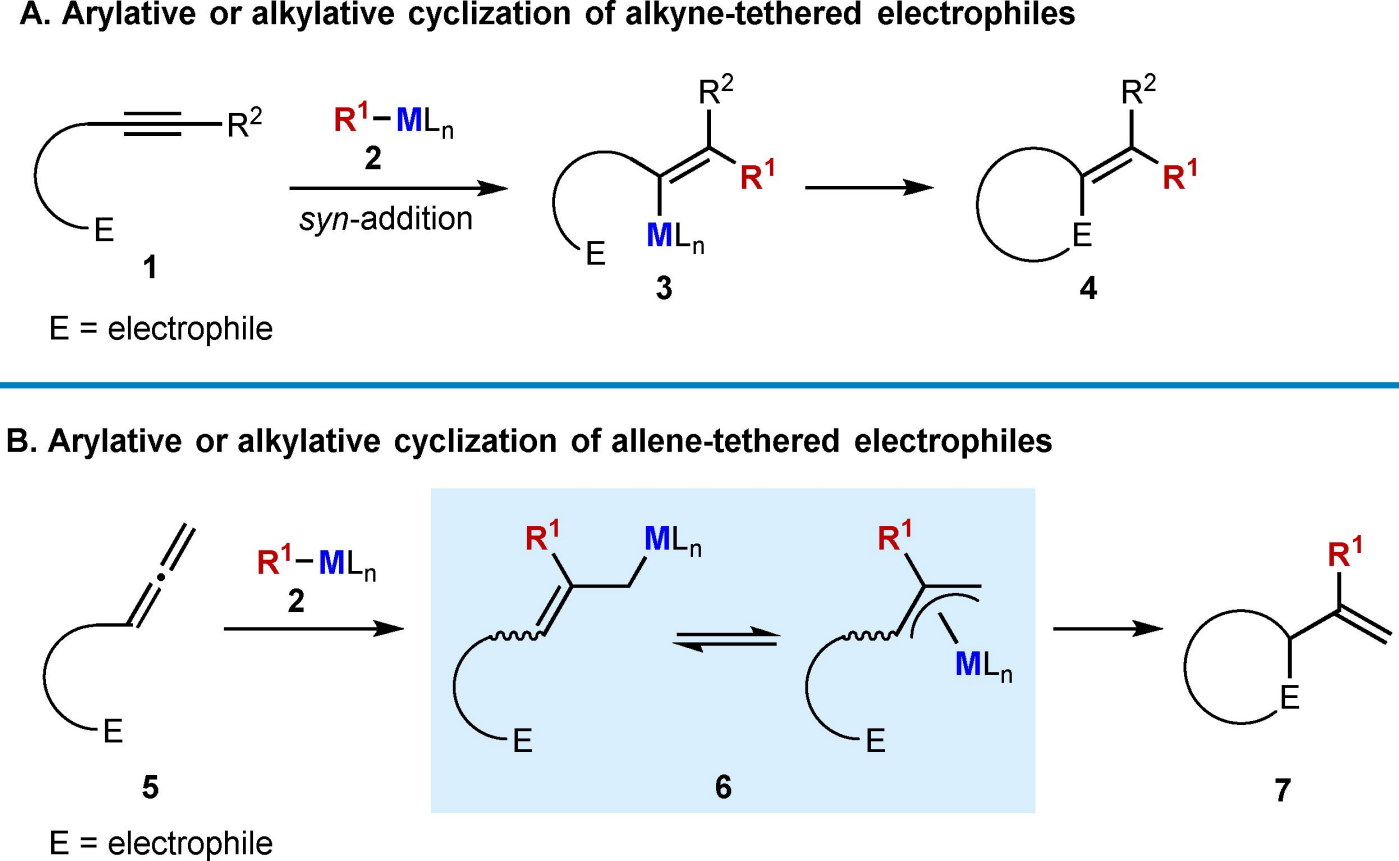
Arylative and alkylative cyclizations of alkyne‐ and allene‐tethered electrophiles by a migratory insertion‐cyclization pathway.

Domino reactions are a versatile tool in synthetic chemistry by combining several bond formation steps into one process, allowing for the synthesis of complex molecules in a step‐economic manner.[^1^, ^2^, ^3^, ^4^, ^5^] Alkyne‐ and allene‐tethered electrophiles are excellent substrates for domino reactions because their multiple reactive sites provide many possibilities for reaction design. These substrates (**1** and **5**) have been used in a wide range of transition‐metal catalyzed arylative and alkylative cyclization reactions to prepare diverse carbo‐ and heterocycles (Scheme 1).[^6^, ^7^, ^8^, ^9^, ^10^, ^11^, ^12^, ^13^, ^14^, ^15^, ^16^, ^17^, ^18^, ^19^, ^20^, ^21^, ^22^, ^23^, ^24^, ^25^, ^26^, ^27^, ^28^, ^29^, ^30^, ^31^, ^32^, ^33^, ^34^, ^35^, ^36^, ^37^, ^38^, ^39^, ^40^, ^41^, ^42^, ^43^, ^44^] These processes typically occur by the reaction of a pronucleophilic reagent with the transition‐metal catalyst to generate an organometallic species **2**. The alkyne or allene of the substrate then undergoes migratory insertion into this organometallic species to generate an alkenylmetal species **3** or allylmetal species **6**, respectively, which can then cyclize onto the tethered electrophile. Overall, two new bonds are formed to give cyclic products, typically of general structure **4** or **7**. It should be mentioned that mechanistically different transition‐metal‐catalyzed arylative and alkylative cyclizations of alkyne‐ and allene‐tethered electrophiles also exist, which proceed via oxidative cyclization to give metallacyclic intermediates,[^45^, ^46^, ^47^, ^48^, ^49^, ^50^, ^51^, ^52^, ^53^, ^54^, ^55^, ^56^, ^57^, ^58^, ^59^] and these reactions are not covered in this review.

Because of their generally good chemical stability, low toxicity, and widespread availability, arylboron compounds are commonly used as pronucleophiles in transition‐metal‐catalyzed domino carbometalation‐cyclization reactions of alkyne‐^[60]^ or allene‐tethered electrophiles. Arylative cyclization reactions of alkyne‐tethered electrophiles involving an arylboron species have been described using rhodium,[^19^, ^20^, ^21^, ^22^, ^23^, ^24^, ^25^, ^26^, ^27^, ^28^, ^29^, ^30^, ^31^, ^32^, ^33^, ^34^] palladium,[^32^, ^33^, ^34^, ^35^, ^36^, ^37^, ^38^, ^39^, ^40^, ^41^] and copper catalysis.^[42]^ Similarly, palladium‐catalyzed arylative cyclization reactions of allene‐tethered electrophiles involving arylboron reagents have been described.[^43^, ^44^] By varying the tether or the electrophile of the substrate, a variety of cyclic products can be obtained, and by employing non‐racemic chiral ligands, enantioselective reactions can be achieved. More recently, nickel catalysis has also been shown to be highly effective in these reactions.[^61^, ^62^, ^63^, ^64^, ^65^, ^66^, ^67^, ^68^, ^69^, ^70^, ^71^, ^72^, ^73^, ^74^, ^75^] As well as being less expensive and more readily available than the more commonly used rhodium or palladium catalysts, nickel catalysis can offer unique possibilities in reaction development not readily available to these other catalyst systems.[^76^, ^77^, ^78^, ^79^, ^80^, ^81^, ^82^, ^83^, ^84^, ^85^, ^86^, ^87^, ^88^, ^89^]

This review will describe nickel‐catalyzed arylative cyclizations of alkyne‐ and allene‐tethered electrophiles using arylboron reagents, that proceed by the general mechanistic pathways shown in Scheme 1. It will highlight the breadth of carbo‐ and heterocyclic compounds that can be obtained and will discuss the scope and limitations of the reactions. The review begins with a consideration of mechanistic aspects of nickel‐catalyzed arylative cyclizations. This section is followed by discussion of various classes of reactions that differ according to whether alkyne‐ or allene‐tethered electrophiles are used, the mode of cyclization taking place, and whether the reactions are enantioselective or not. Related processes that do not utilize alkyne‐ or allene‐tethered electrophiles, but instead involve the annulation of 2‐formyl or 2‐acetylarylboronic acids with alkynes or allenes, are also described because they proceed by similar mechanisms.

Some elements of this review have been covered in other reviews on nickel‐catalyzed alkyne functionalization reactions. Liu and Kong recently reviewed nickel‐catalyzed difunctionalization of alkynes,^[88]^ while Wilger and co‐workers focused on nickel‐catalyzed functionalization of alkynes that proceed with *anti*‐selectivity.^[89]^ These reviews describe both inter‐ and intramolecular alkyne (di)functionalizations using a wide variety of reaction manifolds, whereas our review focuses specifically on arylative cyclizations using organoboron reagents and describes in detail the scope and limitations of these reactions.

## 2 Mechanistic Aspects of Nickel‐Catalyzed Arylative Cyclizations

**Scheme 2:**
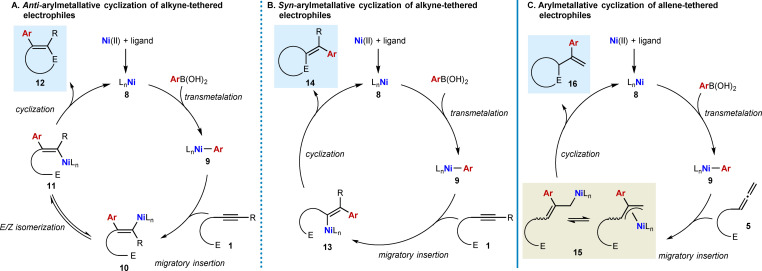
Generalized catalytic cycles for nickel‐catalyzed arylative cyclizations of alkyne‐ or allene‐tethered electrophiles using arylboronic acids.

This section will introduce mechanistic aspects relevant to nickel‐catalyzed arylative cyclizations. Scheme 2 depicts generalized catalytic cycles for the arylative cyclization of alkyne‐ or allene‐tethered electrophiles using arylboronic acids, which are by far the most commonly employed arylboron reagents in these reactions. Only a few examples using other arylboron reagents such as aryl pinacolboronates, aryl trifluoroborates, or arylboroxines have been reported.[^68^, ^73^]

In nickel‐catalyzed arylative cyclizations of alkyne‐tethered electrophiles, two modes of cyclization are generally possible, which differ in both their regio‐ and stereochemical outcomes.^[90]^ The first of these is *anti*‐arylmetallative cyclization.[^17^, ^18^, ^61^, ^62^, ^63^, ^64^, ^65^, ^66^, ^67^, ^68^, ^69^, ^70^] Here, the reactions are initiated by transmetalation of the arylboronic acid with the nickel complex **8** (formed by coordination of a ligand to a nickel(II) salt) to give arylnickel species **9** (Scheme 2A). Coordination of **9** to the alkyne of the substrate **1**, followed by *syn*‐stereospecific migratory insertion of the alkyne places nickel distal to the electrophile. Direct cyclization of the resulting alkenylnickel species **10** onto the electrophile is not possible because of geometric constraints. However, **10** can undergo reversible *E*/*Z* isomerization to give the stereoisomeric alkenylnickel species **11**, which can now cyclize onto the electrophile to give product **12** containing an endocyclic alkene. The mechanism for the *E*/*Z* isomerization step is currently not clear; however, this step has been discussed in some detail by Wilger and co‐workers in their review on nickel‐catalyzed *anti*‐selective alkyne functionalizations.^[89]^ The *E/Z* isomerization of alkenylnickel species has also been observed in other types of reactions.[^91^, ^92^, ^93^, ^94^, ^95^, ^96^] Regarding the oxidation state of nickel in *anti*‐carbometallative cyclizations, different possibilities involving nickel in either the +1 or +2 oxidation states have been proposed.

The second common mode of nickel‐catalyzed arylative cyclization of alkyne‐tethered electrophiles is *syn*‐arylmetallative cyclization (Scheme 2B). The initial steps of the catalytic cycle are identical to those shown in Scheme 2A, to form an arylnickel species **9**. This time, however, *syn*‐stereospecific migratory insertion of the alkyne of the substrate **1** into **9** occurs to place nickel proximal to the electrophile. Direct cyclization of the resulting alkenylnickel species **13** onto the electrophile then occurs to give cyclic products **14** containing an exocyclic alkene.

Because nickel‐catalyzed *anti*‐ and *syn*‐arylmetallative cyclizations require opposite regioselectivities in the alkyne migratory insertion step, the factors that influence this regioselectivity deserve some comment. It is known that alkynes containing an alkyl group on one side and an aryl or alkenyl substituent on the other generally undergo migratory insertion with organometallic species to form alkenylmetal species with the metal adjacent to the aryl or alkenyl group. Presumably, the alkenylmetal species is better stabilized by adjacent sp^2^‐hybridized, rather than sp^3^‐hybridized groups, because the higher s‐character leads to a stronger electron‐withdrawing effect. Therefore, it is not surprising that the majority of nickel‐catalyzed *anti*‐arylmetallative cyclizations of alkyne‐tethered electrophiles employ substrates **1** where R=(hetero)aryl or alkenyl (see Scheme 2A and the reaction scope in Sections 3 and 4) because this results in the selective formation of alkenylnickel species **10**, where nickel is distal to the electrophile. Only a few examples of alkyne‐tethered electrophiles **1** where R=alkyl successfully resulting in *anti*‐arylmetallative cyclization have been reported,[^61^, ^70^, ^73^] but lower yields of products are generally observed [see Tables 1 and 12, and Equation (38)] and in many cases, no desired products were observed.[^62^, ^63^, ^64^, ^65^, ^66^]

In contrast, in nickel‐catalyzed *syn*‐arylmetallative cyclizations of alkyne‐tethered electrophiles, which require migratory insertion to place nickel proximal to the electrophile, substrates containing terminal or dialkyl‐substituted alkynes are typically employed (see the reaction scope in Section 5), though there are examples where an aryl‐alkyl alkyne is employed (Table 16).^[73]^

In nickel‐catalyzed arylative cyclizations of allene‐tethered electrophiles, migratory insertion of the allene of the substrate **5** into the arylnickel species **9** invariably occurs to place the aryl group at the central carbon of the allene to give an allylnickel intermediate **15** (Scheme 2C). Allylnickel intermediate **15** can then cyclize onto the electrophile in a nucleophilic allylation to give product **16** containing a terminal alkene.

As well as arylboron reagents, other organoboron reagents have been employed in nickel‐catalyzed carbometallative cyclizations. Heteroarylboronic acids are well‐known to be more challenging than arylboronic acids in transition‐metal‐catalyzed reactions because of their higher propensity to undergo protodeboronation. However, certain heteroarylboronic acids that are less susceptible to protodeboronation (such as 3‐furyl‐ and 3‐thienylboronic acid), have been successfully employed.[^61^, ^62^, ^63^, ^65^, ^66^, ^67^, ^68^, ^69^, ^70^, ^71^, ^72^, ^73^, ^74^, ^75^, ^113^] The use of alkenylboron reagents in these reactions have also been described [see Tables 7, and Equation (41)][^66^, ^70^, ^75^] although low yields are often observed because of competitive protodeboronation. To our knowledge, no examples of nickel‐catalyzed alkylative cyclization using alkylboron reagents have been reported.

## 3 Non‐Enantioselective *anti*‐Arylmetallative Cyclizations of Alkyne‐Tethered Electrophiles

**Scheme 3:**
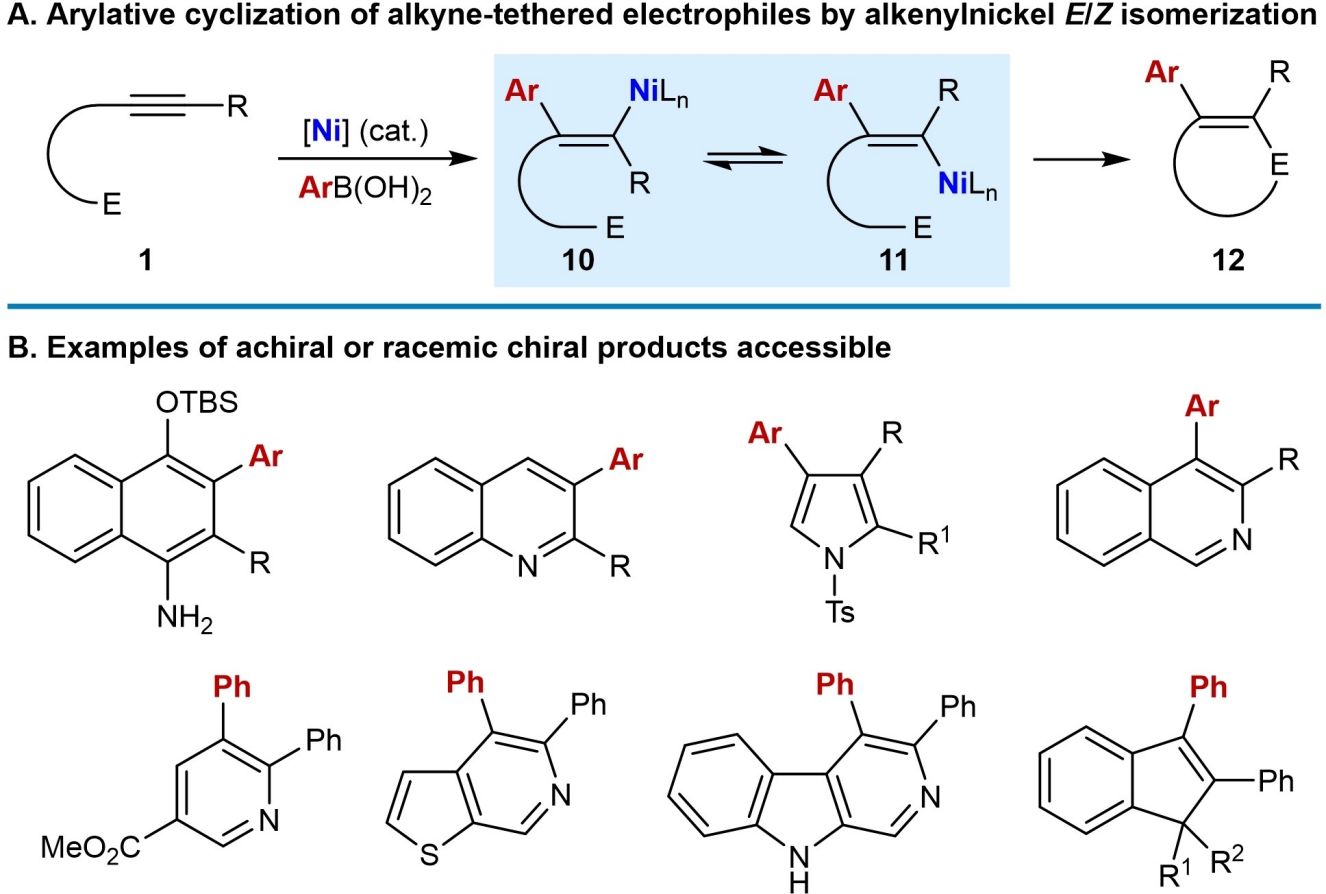
Overview of non‐enantioselective nickel‐catalyzed *anti*‐arylmetallative cyclizations of alkyne‐tethered electrophiles.

As discussed in the previous section, nickel catalysis enables the development of *anti*‐arylmetallative cyclization of alkyne‐tethered electrophiles. A key step in these reactions is the reversible *E*/*Z* isomerization of the intermediate alkenylnickel species (Scheme 2A and 3A). This section describes non‐enantioselective arylative cyclizations of alkyne‐tethered electrophiles that produce either achiral products or racemic chiral products. These reactions encompass a wide range of electrophiles that includes nitriles, azides, *N*‐tosyl amides, ketones, and α,β‐unsaturated ketones to give diverse products such as 1‐naphthylamines, quinolines, pyrroles, isoquinolines, pyridines, thiophenopyridines, β‐carbolines, and indenes (Scheme 3B).

In 2016, Liu and co‐workers reported the seminal report of nickel‐catalyzed *anti*‐arylmetallative cyclizations of alkyne‐tethered electrophiles using arylboronic acids, which involves the cyclization of alkenylnickel intermediates onto nitriles (Table 1).^[61]^ 2‐(Cyano)phenyl propargyl ethers were reacted with an arylboronic acid (2.0 equiv.), Ni(acac)_2_ ⋅ 2H_2_O (10 mol %), P(4‐F_3_CC_6_H_4_)_3_ (10 mol %), and Cs_2_CO_3_ (0.2 equiv.) in 1,4‐dioxane at 90 °C to give highly functionalized 1‐naphthylamines. As well as phenylboronic acid (**18 a**), the reaction tolerates electron‐withdrawing (**18 b**) and electron‐donating (**18 c** and **18 d**) groups on the arylboronic acid. However, the use of alkylboronic acids such as *n*‐butylboronic acid did not give the desired products. Aryl groups on the alkyne are tolerated (**18 a**–**18 e**) as are heteroaryl groups such as 2‐thienyl (**18 f**) and 3‐benzothienyl (**18 g**). Furthermore, cyclohexenyl (**18 h**), *n*‐propyl (**18 i**), and cyclopropyl (**18 j**) substituents on the alkyne are compatible with the reaction; however, lower yields were obtained. The latter two cases are rare examples of alkyl substitution on the alkyne in nickel‐catalyzed *anti*‐arylmetallative cyclization. As discussed in section 2, a (hetero)aryl or alkenyl substituent on the alkyne is generally necessary to obtain high regioselectivities in the migratory insertion step and the low yields of **18 i** and **18 j** may be a consequence of lower regioselectivity.

**Table 1:**
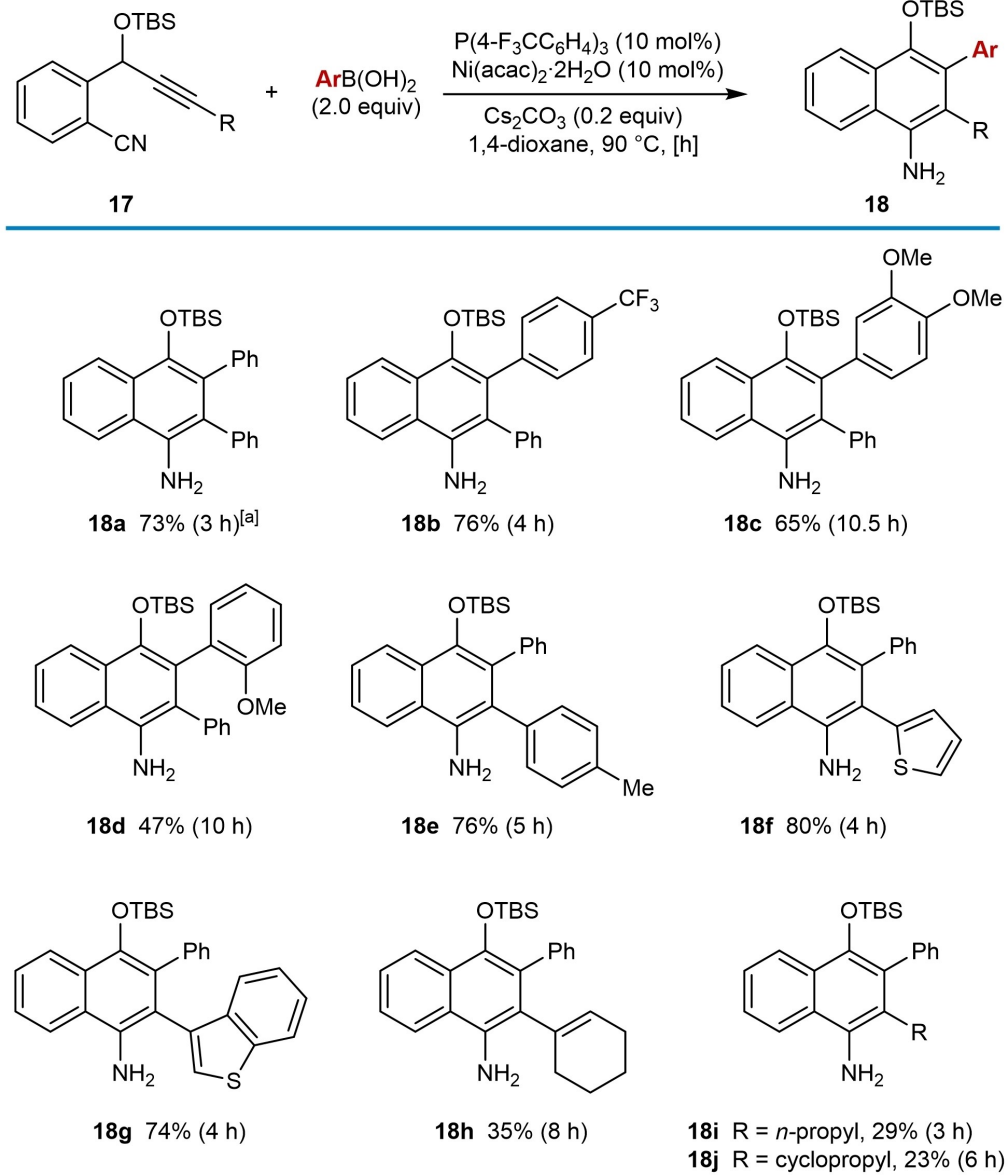
Synthesis of 1‐naphthylamines by *anti*‐arylmetallative cyclization onto nitriles.

[a] Using Ni(acac)_2_ ⋅ 2H_2_O (5 mol %), P(4‐F_3_CC_6_H_4_)_3_ (5 mol %) and Cs_2_CO_3_ (0.1 equiv.).

**Scheme 4:**
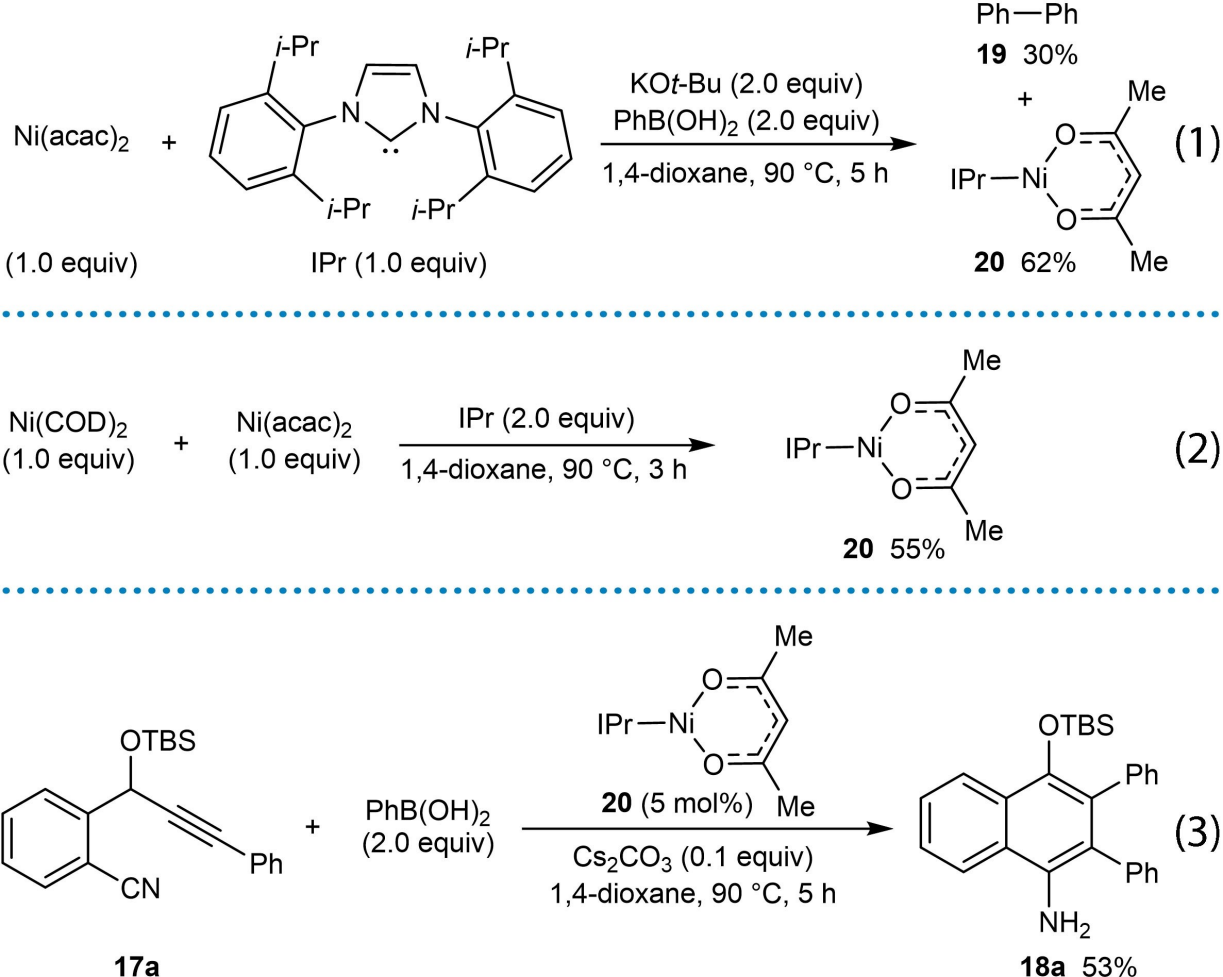
Mechanistic studies.

Experiments were carried out to gain mechanistic insight into the reactions (Scheme 4). To gain insight into the oxidation state of the active nickel species, Ni(acac)_2_ was reacted with 1,3‐bis(2,6‐diisopropylphenyl)‐1,3‐dihydro‐2*H*‐imidazol‐2‐ylidene (IPr), which was also found to be an effective ligand during optimization studies, in the presence of two equivalents each of KO*t*‐Bu and PhB(OH)_2_ [Eq. (1)]. This experiment gave biphenyl (**19**) in 30 % yield and the three‐coordinate distorted T‐shaped Ni(I) complex **20** in 62 % yield, which was characterized by X‐ray crystallography. It was suggested that biphenyl (**19**) is formed by reductive elimination of a biarylnickel(II) species which would result in the release of a Ni(0) species. A comproportionation reaction between Ni(0) and Ni(II) could then provide the observed Ni(I) species **20**. The stoichiometric reaction of Ni(COD)_2_ and Ni(acac)_2_ in the presence of IPr (2.0 equiv.) was carried out and provided Ni(I) species **20** in 55 % yield [Eq. (2)], which provides some support for this hypothesis. Ni(I) complex **20** was also found to catalyze the arylative cyclization reaction of 2‐(cyano)phenyl propargyl ether **17 a** with PhB(OH)_2_ to give the desired product **18 a** in 53 % yield, suggesting that a Ni(I) species is catalytically competent [Eq. (3)]. The proposed mechanism for the nickel‐catalyzed *anti*‐arylmetallative cyclization of alkyne‐tethered nitriles follows the general catalytic cycle shown in Scheme 2A with nickel in the +1 oxidation state.

In 2018, Reddy and co‐workers reported the synthesis of 2,3‐diarylquinolines by the nickel‐catalyzed *anti*‐arylmetallative cyclization of azidophenyl propargyl alcohols **21** with arylboronic acids (1.2 equiv.) in the presence of Ni(acac)_2_ (10 mol %), PPh_3_ (10 mol %), and Cs_2_CO_3_ (0.2 equiv.) in 1,4‐dioxane at 90 °C. (Table 2).^[62]^ The reaction tolerates a range of arylboronic acids including 4‐bromophenylboronic acid (**23 b**) as well as 1,3‐benzodioxole‐5‐boronic acid (**23 c**). The reaction works well with electron‐donating (**23 d**) or electron‐withdrawing (**23 e**) aryl groups on the alkyne. A gram‐scale reaction using phenylboronic acid gave **23 a** in 86 % yield. The reaction of a substrate containing a cyclohexenyl‐substituted alkyne with phenylboronic acid gave **23 f** in 71 % yield. A substrate with an alkyl azide did not afford any of the product **23 g**.

**Table 2:**
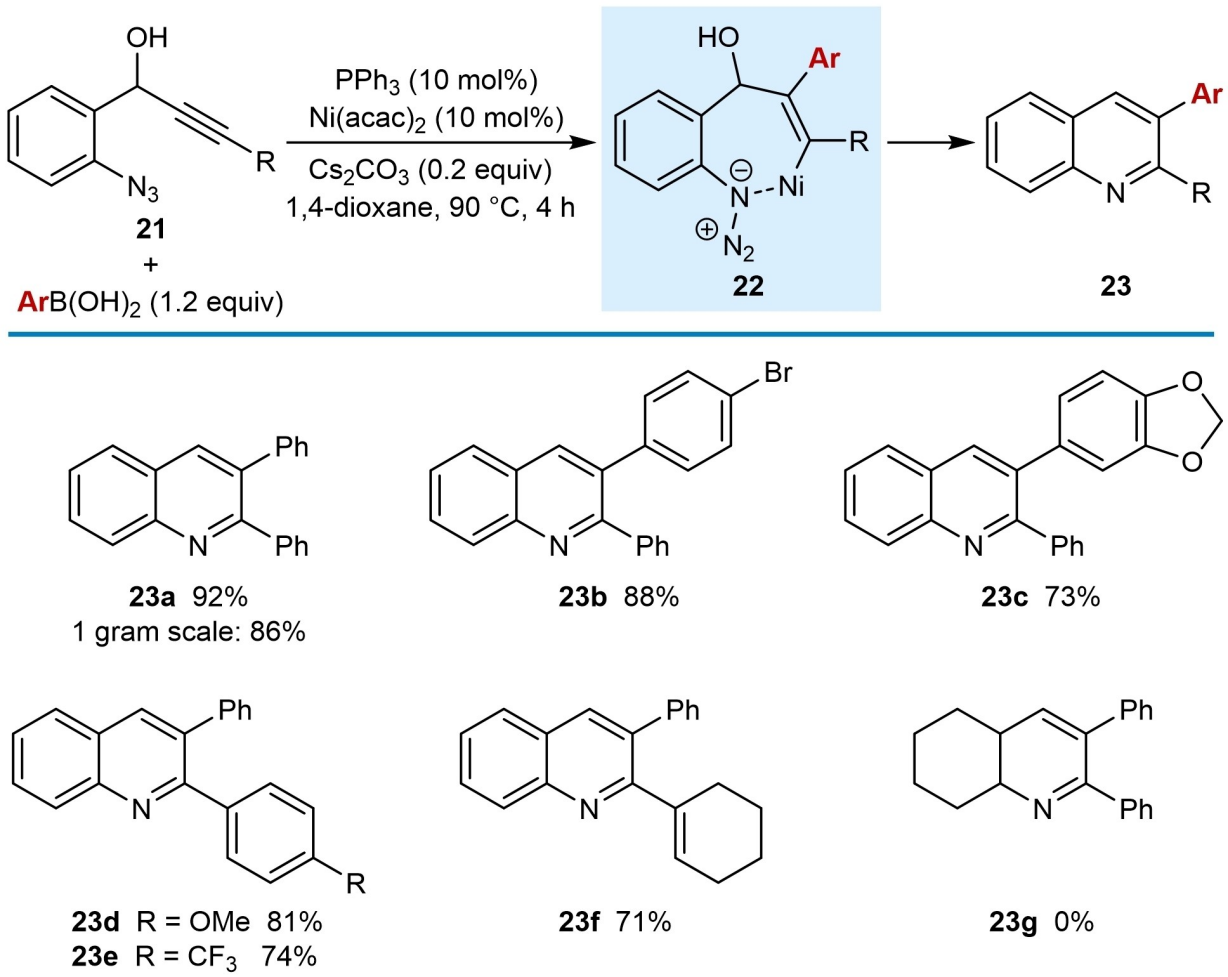
Synthesis of quinolines by *anti*‐arylmetallative cyclizations onto azides.

The proposed mechanism follows the general catalytic cycle shown in Scheme 2A, with the intermediate alkenylnickel species cyclizing onto the azide (as in **22**) to eject dinitrogen as a leaving group. The authors proposed that nickel adopts the +2 oxidation state throughout the catalytic cycle, in contrast to the proposal by Liu and co‐workers for their nickel‐catalyzed synthesis of 1‐naphthylamines (Table 1).^[61]^

The propargylic hydroxyl group in the substrates is important for the success of the reaction, as shown by the reaction of a substrate without this functionality, which led only to slow decomposition and none of the desired 2,3‐diarylquinoline being formed [Eq. 4].

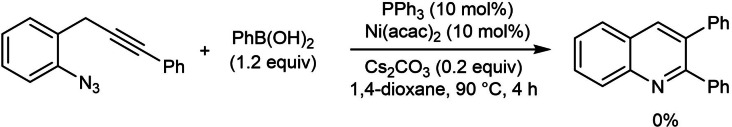




In 2018, Lam and co‐workers described the synthesis of multisubstituted pyrroles by the nickel‐catalyzed arylative cyclization of *N*‐tosyl alkynamides with (hetero)arylboronic acids (2.0 equiv.) (Table 3).^[63]^ The conditions employed were 5 mol % each of Ni(OAc)_2_ ⋅ 4H_2_O and racemic Ph‐PHOX (*rac*‐**L1**) in TFE at 80 °C. The process is tolerant of a range of substituents on the alkyne such as phenyl (**26 a**), 2‐fluorophenyl (**26 b**), 2‐thienyl (**26 c**), and an alkenyl group (**26 d**); however, a substrate with a methyl‐substituted alkyne led to a complex mixture of products. Excellent yields were obtained with various aryl (**26 a**–**26 e**) or alkyl substituents (**26 f**–**26 h**) on the *N*‐acyl group. Substituted phenylboronic acids are tolerated in the reaction (**26 i**) as well as heteroarylboronic acids such as 5‐indolyl (**26 j**), 3‐thienyl (**26 k**), and 3‐furylboronic acid (**26 l**). However, 4‐pyridinylboronic acid, methylboronic acid, and cyclopropylboronic acid did not provide the desired products. The proposed mechanism follows the generalized catalytic cycle shown in Scheme 2A, with cyclization of the intermediate alkenylnickel species onto the *N*‐acyl group giving nickel alkoxide **25** (Table 3, top). Protonation of **25**, followed by elimination of water, gives the pyrrole products.

**Table 3:**
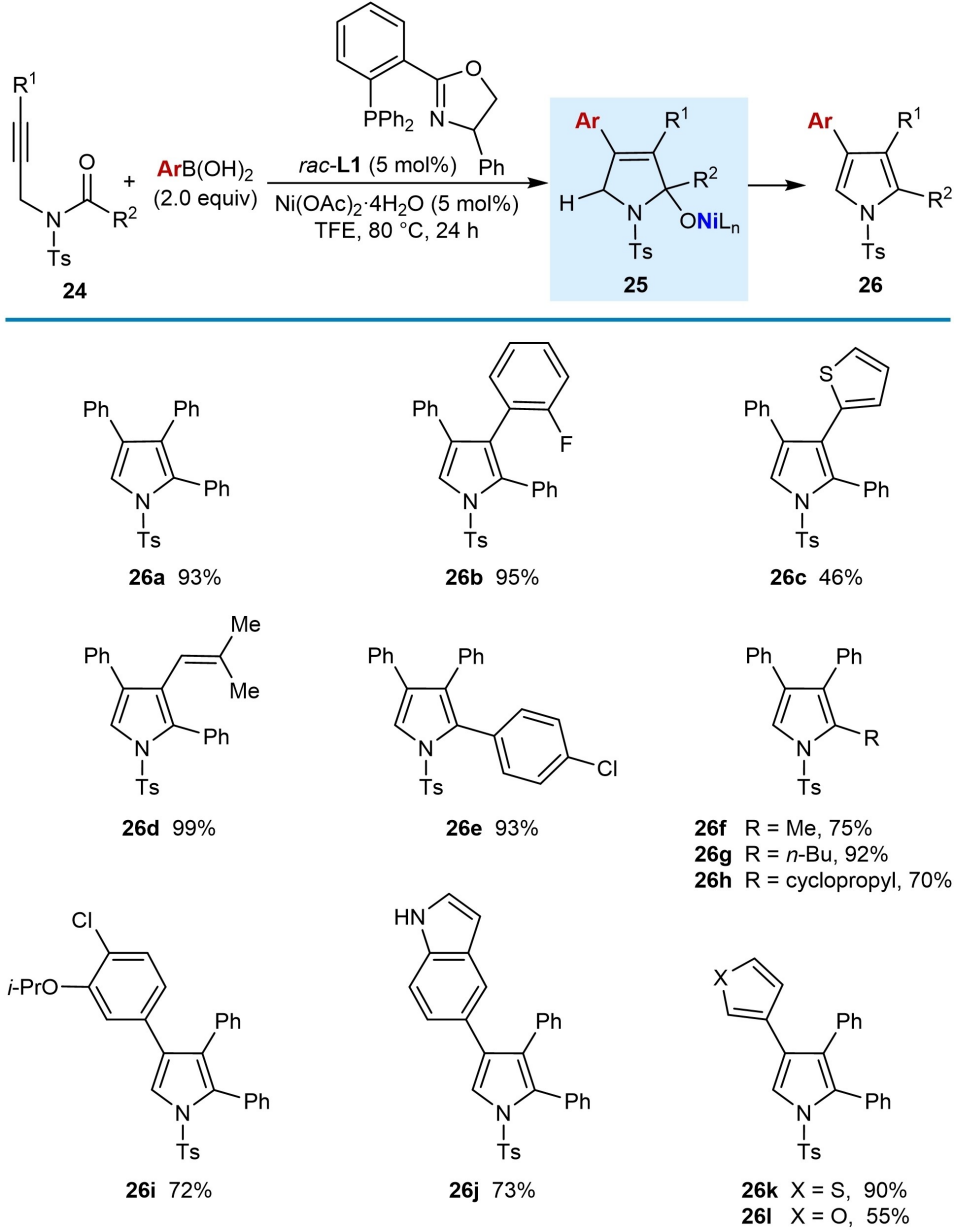
Synthesis of pyrroles by *anti*‐arylmetallative cyclizations onto *N*‐tosylamides.

**Scheme 5:**
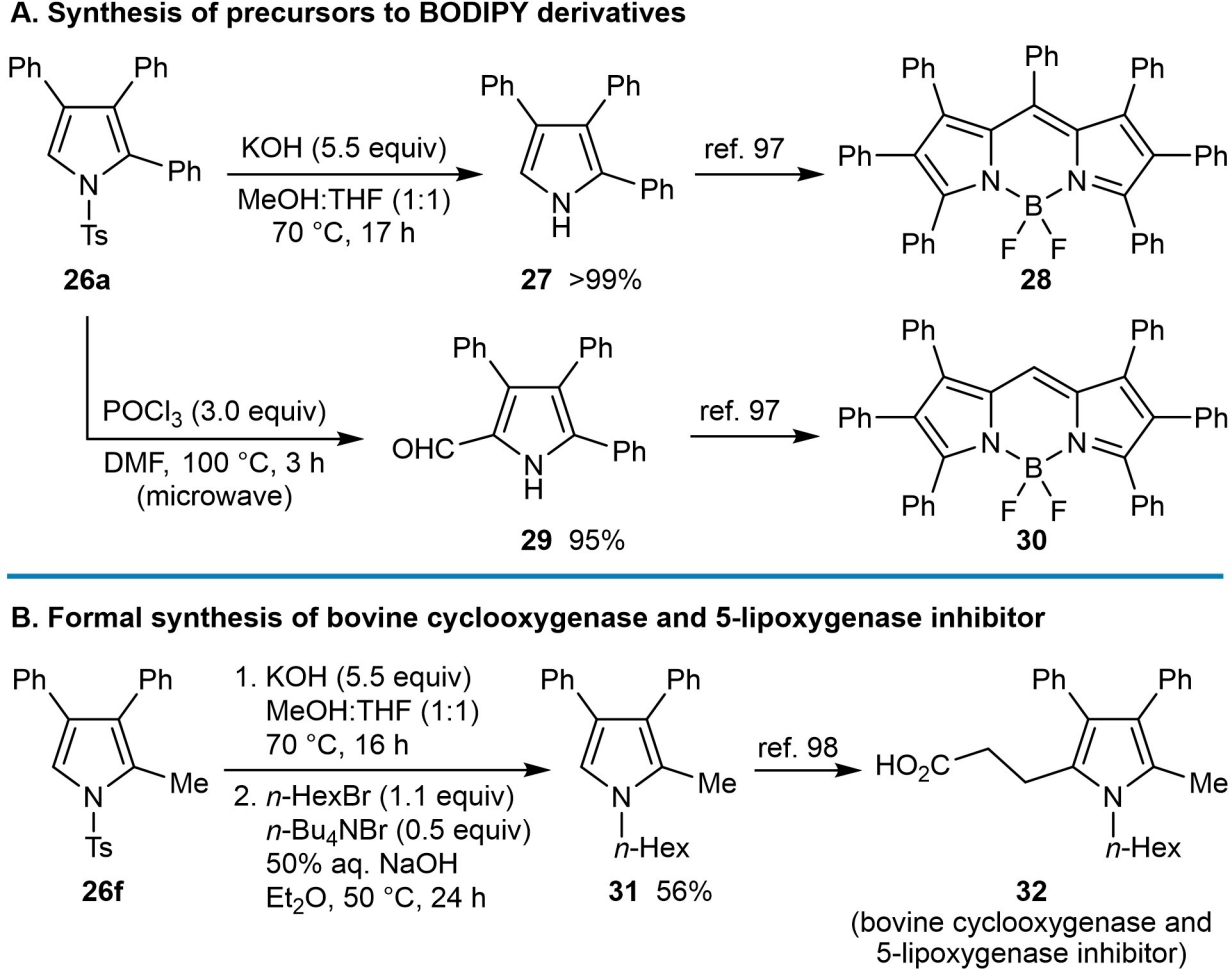
Synthetic applications of pyrroles prepared by nickel‐catalyzed arylmetallative cyclizations.

The utility of this process was demonstrated in the synthesis of pyrroles **27** and **29** that have been used in the preparation of 4,4‐difluoro‐4‐bora‐3*a*,4*a*‐diaza‐*s*‐indacene (BODIPY) derivatives **28** and **30** ^[97]^ (Scheme 5A) and bovine cyclooxygenase and 5‐lipoxygenase inhibitor **32** ^[98]^ (Scheme 5B). Removal of the tosyl group from pyrrole **26 a** was achieved using KOH in MeOH:THF (1 : 1) at 70 °C to obtain pyrrole **27**, a precursor to BODIPY derivative **28**. In addition, the reaction of **26 a** with POCl_3_ in DMF at 100 °C led to formylation with concomitant tosyl deprotection to give pyrrole **29**, a precursor to BODIPY derivative **30**. A further application was described in the synthesis of pyrrole **31**, a precursor to the bovine cyclooxygenase and 5‐lipoxygenase inhibitor **32**, through tosyl deprotection of **26 f** followed by *N*‐alkylation with *n*‐hexyl bromide.

**Scheme 6:**
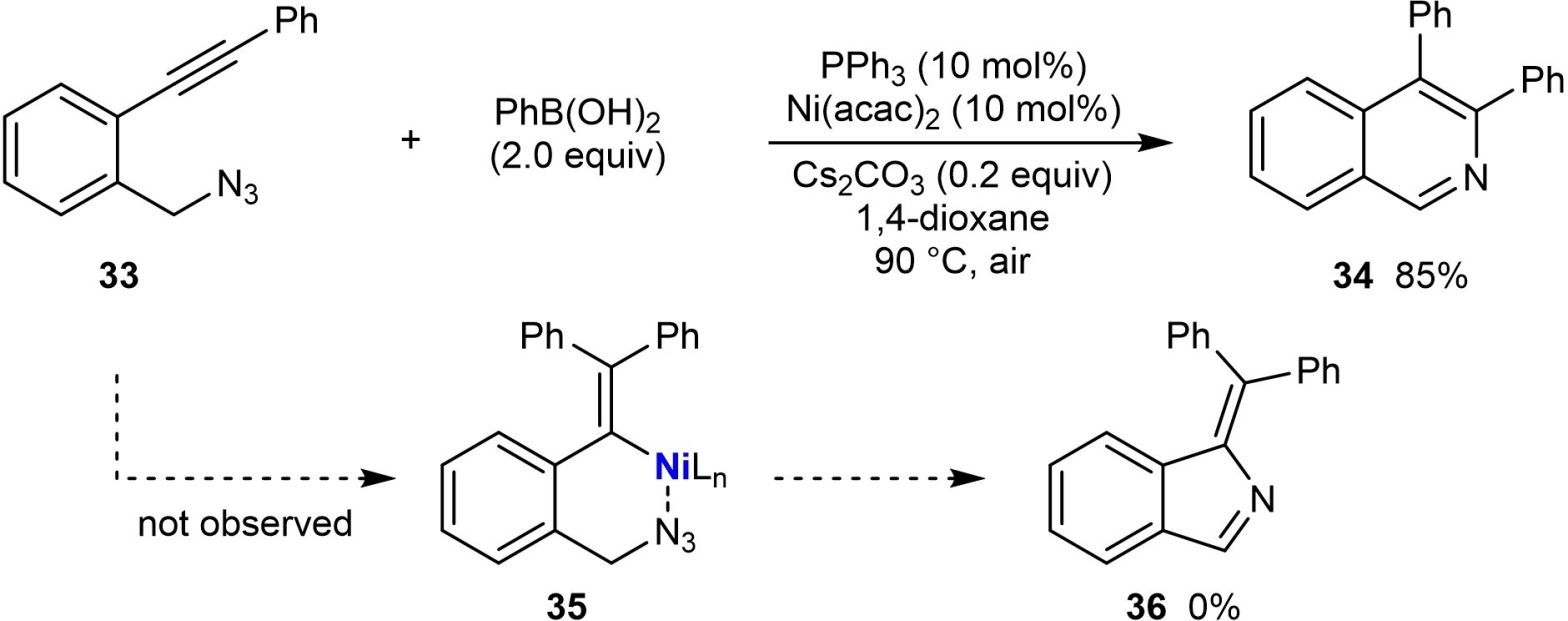
Nickel‐catalyzed arylative cyclizations of *orth*o‐functionalized diarylalkynes.

In 2020, Reddy and co‐workers reported the nickel‐catalyzed arylative cyclization of substrates containing seemingly electronically and sterically unbiased diaryl alkynes to give various pyridine and indene derivatives [Scheme 6 and Equations (7)‐(17)].^[64]^ The reactions were conducted by heating the substrate with the (hetero)arylboronic acid (2.0 equiv.), Ni(acac)_2_ (10 mol %), PPh_3_ (10 mol %), and Cs_2_CO_3_ (0.2 equiv.) in 1,4‐dioxane under air at 90 °C. The arylative cyclization reaction of substrate **33**, which contains an azide and a diarylalkyne, with phenylboronic acid led to the isoquinoline **34** in 85 % yield (Scheme 6). This result was initially surprising because with a diarylalkyne one might have expected some of the alternative product **36** to be formed, resulting from migratory insertion of the alkyne with the intermediate arylnickel species with the opposite regioselectivity to give **35**. Indeed, nickel‐catalyzed hydroarylation of an diarylalkyne that lacks the azido group led to a mixture of regioisomers **37** and **38** [Eq. (5)], which suggests the presence of the electrophile is important in controlling regioselectivity.

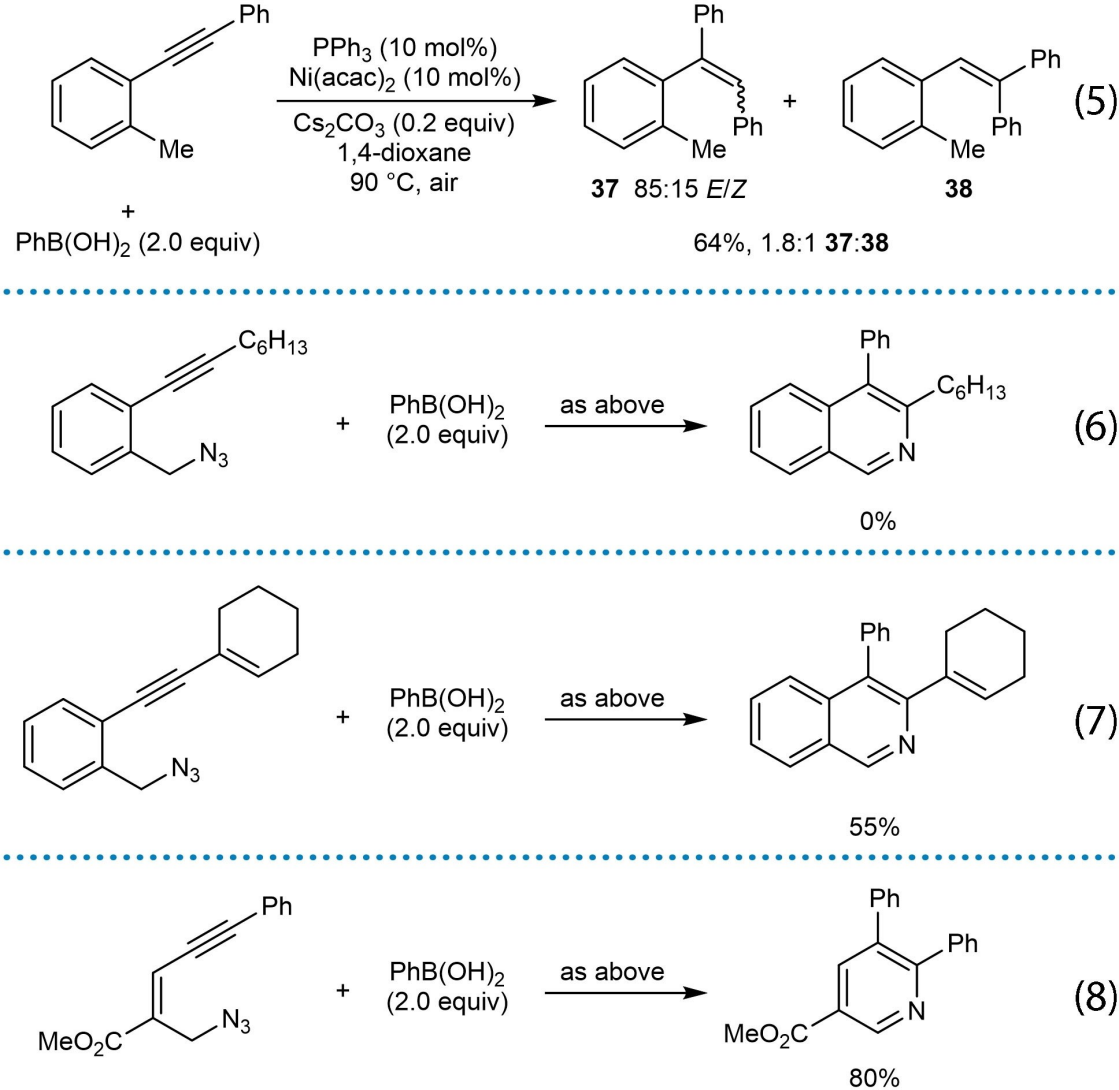




**Scheme 7:**
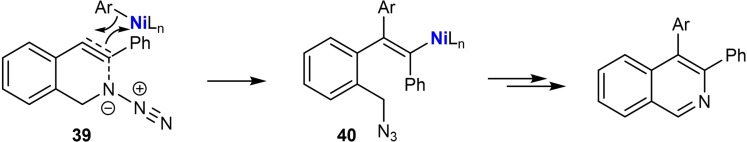
Proposed electrophile‐driven migratory insertion.

A substrate containing an alkyl‐substituted aryl alkyne did not provide any of the desired isoquinoline [Eq. (6)]. However, substrates containing an aryl group and an alkenyl group on either side of the alkyne in one of two alternative connectivities successfully led to the desired products [Eq. (7) and (8)]. These results further highlight the importance of the electrophile in these arylative cyclizations of alkynes that have seemingly electronically unbiased alkynes. The authors suggest two possible mechanisms for these reactions. In the first possibility, favored by the authors, it was proposed that the regioselectivity of the migratory insertion of the alkyne with the arylnickel species is controlled by a polarizing effect of the tethered electrophile (as in **39**) as opposed to any steric or electronic effect of the alkyne substituents, to give the alkenylnickel species **40** (Scheme 7). Following the generalized catalytic cycle in Scheme 2A, **40** can then undergo reversible *E*/*Z* isomerization and cyclization onto the azide to eventually give the isoquinoline. In this mechanism, the oxidation state of nickel was not specified. The second suggested mechanism involves an initial *“anti*‐Wacker“‐type addition.[^32^, ^99^, ^100^]

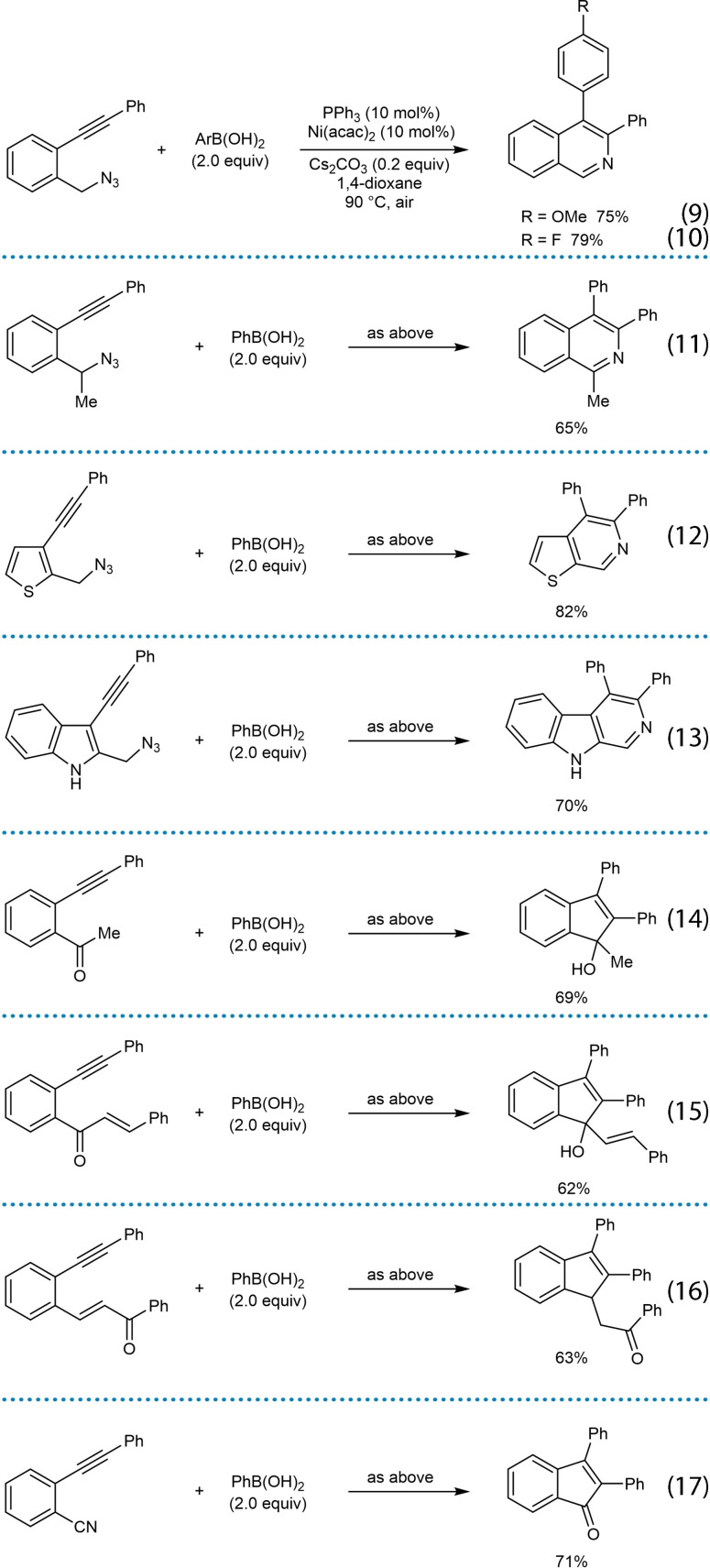




Further examples of the scope of this process are shown in Equations (9)‐(17). Arylative cyclization with substituted phenylboronic acids bearing methoxy [Eq. (9)] or fluoride groups [Eq. (10)] worked well. A substrate containing a secondary alkyl azide led to a trisubstituted isoquinoline in 65 % yield [Eq. (11)]. Thiophenopyridines [Eq. (12)] and β‐carbolines [Eq. (13)] were successfully prepared from thiophene‐ and indole‐containing substrates, respectively. The reaction also worked with substrates containing other electrophiles such as ketones [Eq. (14) and (15)] or conjugated enones [Eq. (16)], leading to the synthesis of racemic chiral indenes. Arylative cyclization onto a nitrile gave an indenone [Eq. (17)].

## 4 Enantioselective Arylative Cyclization of Alkyne‐Tethered Electrophiles Involving Reversible Alkenylnickel *E*/*Z* Isomerization

**Scheme 8:**
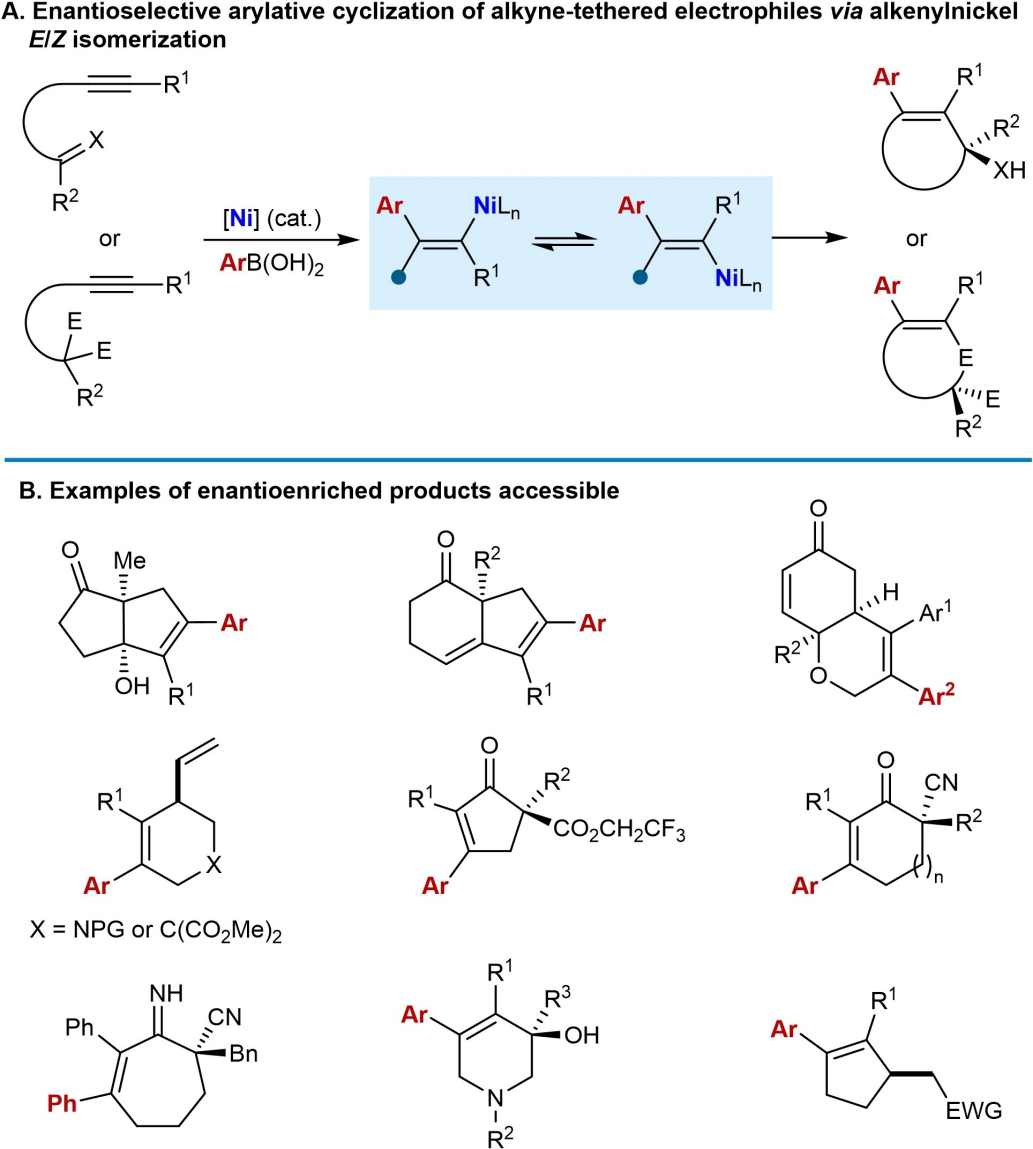
Overview of enantioselective nickel‐catalyzed *anti*‐arylmetallative cyclizations of alkyne‐tethered electrophiles.

Enantioselective variants of arylative cyclizations of alkyne‐tethered electrophiles involving reversible alkenylnickel *E*/*Z* isomerization have been achieved using chiral phosphine–oxazoline ligands (Scheme 8A). A diverse range of functionalized carbo‐ and heterocyclic compounds containing tertiary or quaternary centers have been prepared using this strategy (Scheme 8B), often via desymmetrization reactions, and the range of electrophiles used include ketones, electron‐deficient alkenes, allylic phosphates, esters, and nitriles.

In 2016, Lam and co‐workers reported the first example of enantioselective nickel‐catalyzed *anti*‐carbometallative cyclization of alkyne‐tethered electrophiles involving reversible alkenylnickel *E*/*Z* isomerization (Table 4).^[65]^ Treatment of substrates **41**, which contain an aryl alkyne tethered to a cyclic 1,3‐diketone, with a (hetero)arylboronic acid (2.0 equiv.), Ni(OAc)_2_ ⋅ 4H_2_O (10 mol %), and (*R*)‐Ph‐PHOX (**L1**, 10 mol %) in a 3 : 2 mixture of MeCN and 2‐MeTHF at 80 °C gave fused bicyclic products **42** with often high enantioselectivities. As well as phenylboronic acid (**42 a**), 4‐substituted (**42 b**) and 2‐substituted (**42 c**) phenylboronic acids are tolerated in the reaction; however, 2‐fluorophenylboronic acid led to a lower yield of the corresponding product **42 c** but with a higher enantioselectivity. 3‐Thienylboronic acid is also effective (**42 d**) but a decrease in enantioselectivity was observed. The use of alkenylboronic acids instead of arylboronic acids did not lead to any desired products. The reaction is tolerant of a range of aryl groups on the alkyne, including those with methoxy (**42 e**) or chloro substituents (**42 f**). None of the desired products were obtained with substrates containing a terminal alkyne, methyl alkyne, or trimethylsilyl‐substituted alkyne, though in the latter two cases, some success was obtained using the achiral ligand 2‐[2‐(diphenylphosphino)ethyl]pyridine (pyphos) in place of **L1** to give racemic products. Arylative cyclization onto an indan‐1,3‐dione led to the tricyclic product **42 g** in 70 % yield and 42 % ee.

**Table 4:**
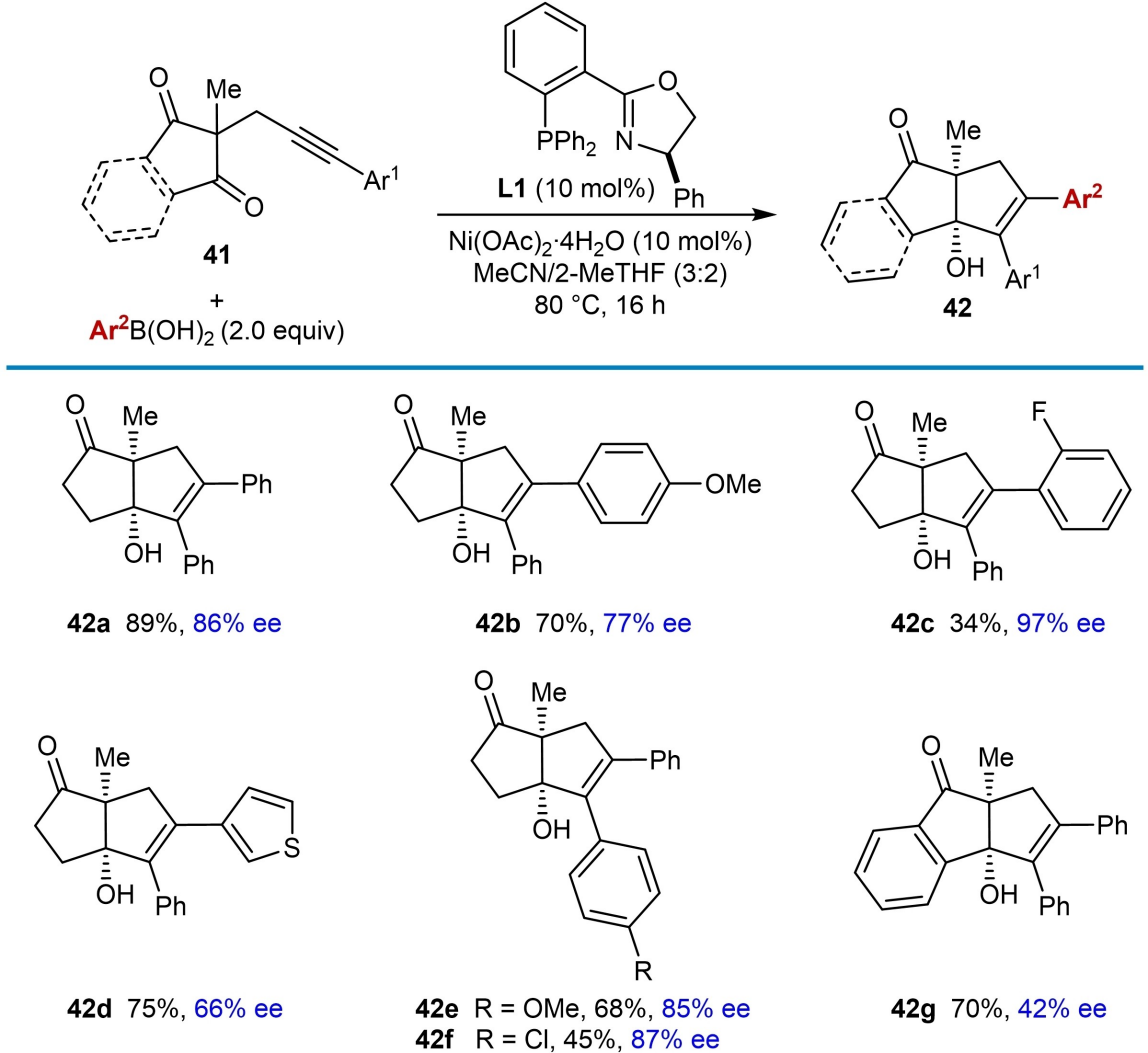
Enantioselective *anti*‐arylmetallative cyclizations onto five‐membered cyclic 1,3‐diketones.

Six‐membered cyclic 1,3‐diketones are also effective electrophiles in this process (Table 5). However, under the standard conditions, mixtures of the expected tertiary‐alcohol‐containing cyclization product and dehydration product (**44**) were obtained. Therefore, after cyclization was complete, 20 % H_2_SO_4_ in AcOH was added to drive the dehydration reaction to completion. Compared with the corresponding reactions of five‐membered cyclic 1,3‐diketones, the products **44 a**–**44 c** were obtained in generally higher enantiomeric excesses.

**Table 5:**
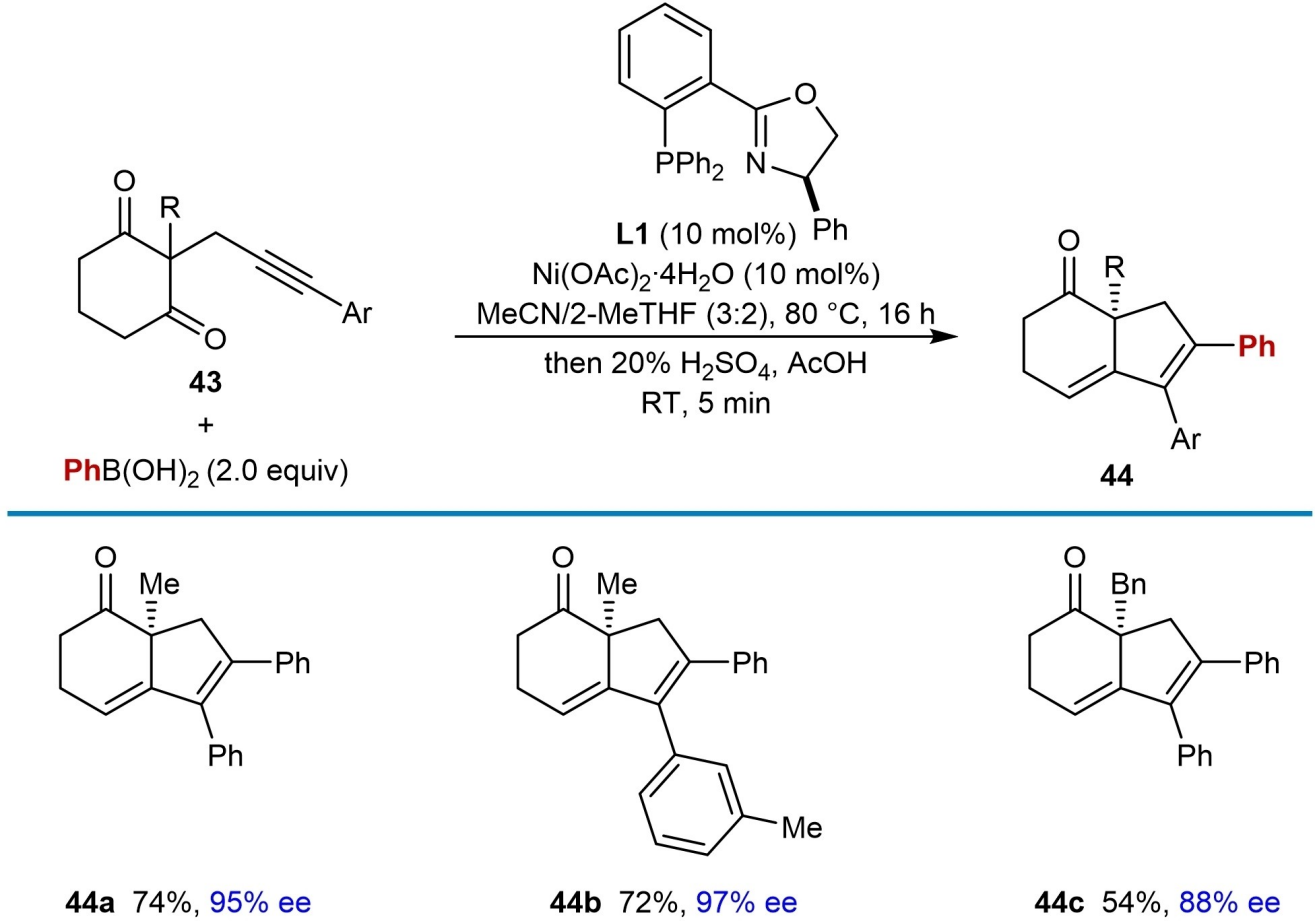
Enantioselective *anti*‐arylmetallative cyclizations onto six‐membered cyclic 1,3‐diketones.

Changing the electrophile from cyclic 1,3‐diketones to cyclohexa‐2,5‐dienones in substrates **45** was also investigated and the products **46** were isolated together with small quantities of minor products **47**, which resulted from migratory insertion of the alkyne into the intermediate arylnickel species with the opposite regioselectivity (Table 6). As well as phenylboronic acid (**46 a**), 4‐acetylphenylboronic acid (**46 b**), and 3‐thienylboronic acid (**46 c**) are tolerated. The substituent at the quaternary center of the substrates can be changed from a methyl (**46 a**–**46 c** and **46 f**) to an ethyl group (**46 d**); however, a phenyl group led to a lower yield and enantioselectivity (**46 e**, 20 %, 69 % ee). A substrate containing a 4‐cyanophenyl group on the alkyne also gave good results (**46 f**).

**Table 6:**
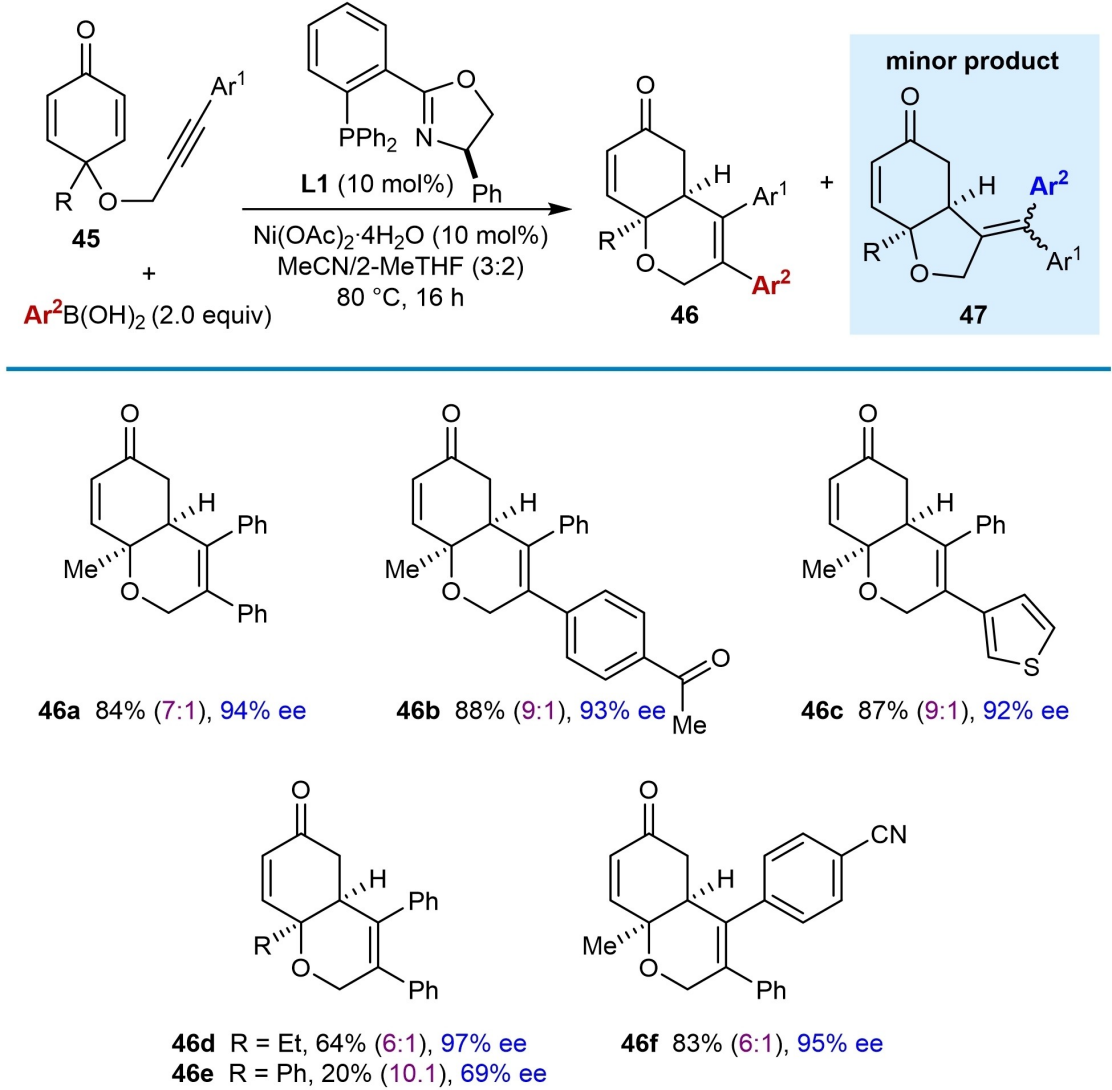
Enantioselective *anti*‐arylmetallative cyclizations onto cyclohexa‐2,5‐dienones.

Regarding the proposed mechanism, the authors suggested a catalytic cycle analogous to the one shown in Scheme 2A with nickel in the +2 oxidation state throughout; however, they do not rule out alternative mechanisms involving Ni(I) species, for example as suggested by Liu and co‐workers.^[61]^

In 2020, Kong and co‐workers reported reactions similar to the arylative cyclizations onto cyclic 1,3‐diketones shown in Tables 4 and 5; however, this process is a reductive cyclization using aryl bromides instead of arylboronic acids, and manganese was used as a stoichiometric reductant [Eq. (18)].^[17]^ One enantioselective example using (*S*)‐Ph‐PHOX (*ent*‐**L1**) was reported, which gave **42 a** in 65 % yield and 81 % ee [Eq. 18].

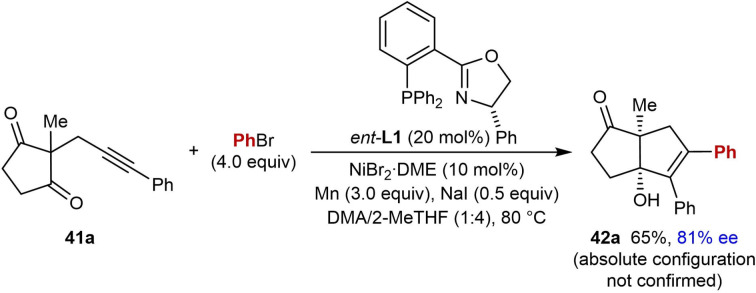




In 2017, the Lam group reported enantioselective nickel‐catalyzed intramolecular arylative allylic alkenylations, where the intermediate alkenylnickel species cyclizes onto a *Z‐*allylic phosphate to give chiral 1,4‐diene‐containing hetero‐ and carbocycles (Table 7).^[66]^ These reactions used (*S*)‐*t*‐Bu‐NeoPHOX (**L2**) as the chiral ligand in TFE as the solvent, and excellent enantioselectivities were observed. Similar to cyclizations onto cyclohexa‐2,5‐dienones reported previously (Table 6),^[65]^ small quantities of minor products **50** were observed in most of these reactions. Substrates containing aryl‐ (**49 a**, **49 b** and **49 f**–**49 i**), heteroaryl‐ (**49 d** and **49 e**), or alkenyl‐substituted (**49 c**) alkynes are effective in the reaction; however, the alkenyl‐substituted alkyne gave a decreased yield and enantioselectivity (**49 c**, 45 %, 49 % ee). The use of a methyl‐substituted alkyne led to a complex mixture of products, which is similar to other reports of nickel‐catalyzed *anti*‐carbometallative cyclizations using dialkyl alkynes.[^62^, ^63^, ^64^, ^65^] A disubstituted phenylboronic acid worked in the reaction (**49 d**), as did 2‐naphthylboronic acid (**49 e**). Interestingly, using an alkenylboronic acid was also successful; however, the product **49 f** was obtained in a low yield (13 %) most likely due to extensive protodeboronation of the alkenylboronic acid. No reaction occurred when methylboronic acid was used. Variation of the tethering group showed that a 4‐nitrophenylsulfonamide is compatible (**49 g**). Substrates containing an all‐carbon tether also cyclized successfully to give carbocyclic products **49 h** and **49 i**. The proposed mechanism follows the general catalytic cycle shown in Scheme 2A with nickel in the +2 oxidation state throughout; however, an additional β‐phosphate elimination step of intermediate **51** is required to liberate the product **49** and regenerate the active Ni(II) species [Eq. 19].

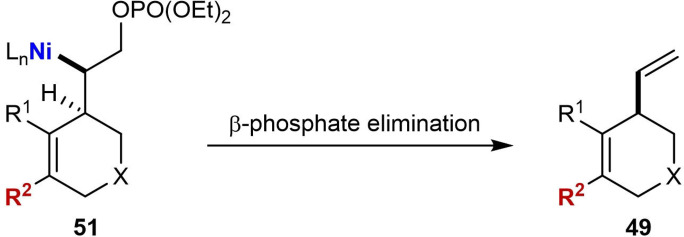




**Table 7:**
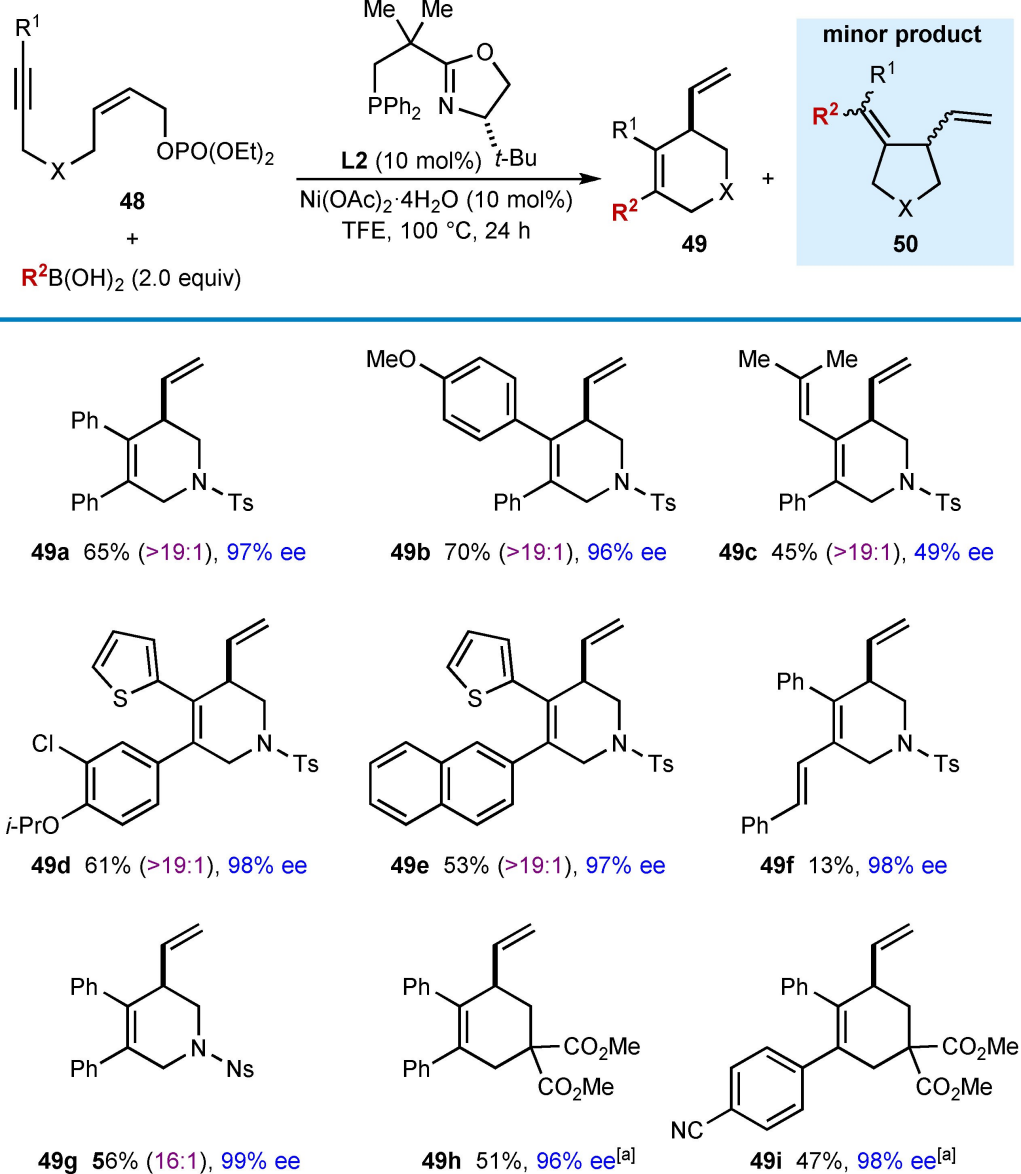
Enantioselective *anti*‐arylmetallative cyclizations onto *Z*‐allylic phosphates.

Yields are of isolated products **49**, free from the minor isomers **50**. [a] The product contained trace quantities of inseparable, unidentified impurities, and the ratio of **49 : 50** could not be determined.

**Scheme 9:**
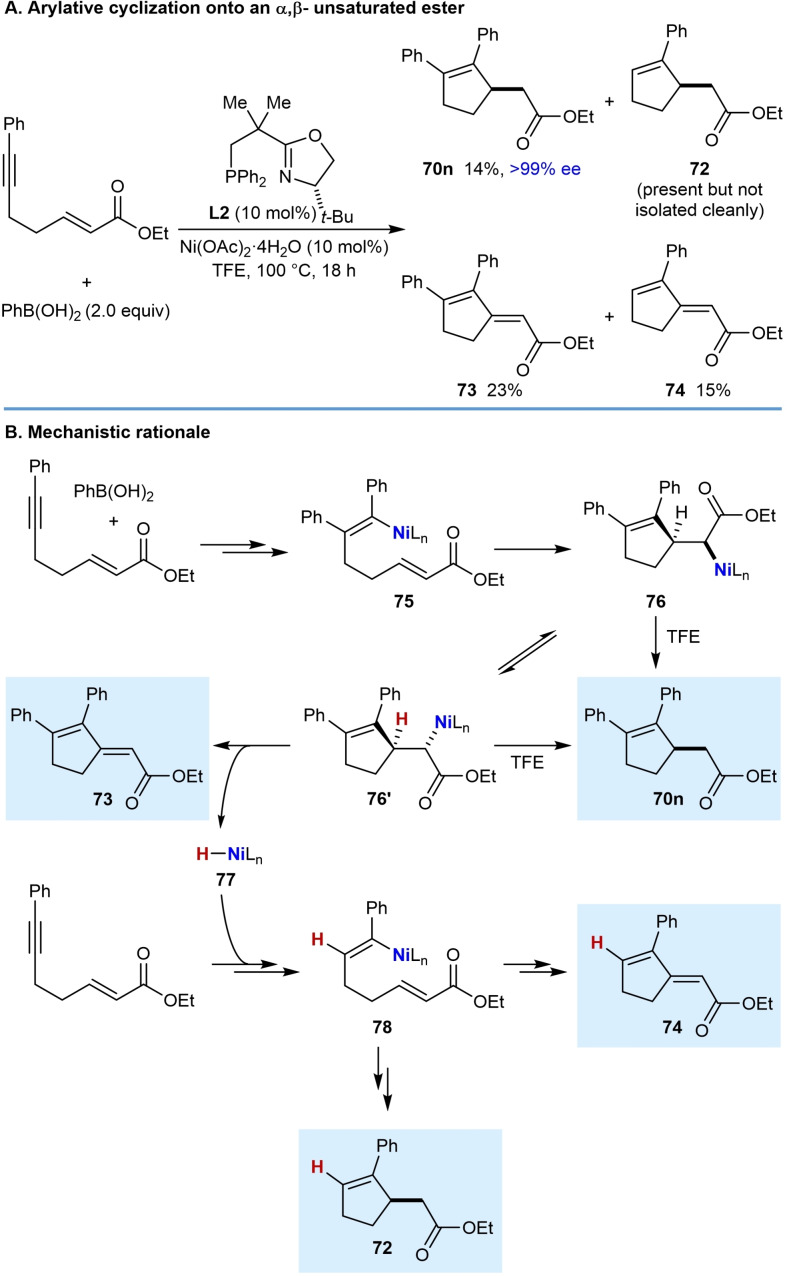
Enantioselective *anti*‐arylmetallative cyclization onto an α,β‐unsaturated ester and mechanistic rationale.

The attempted arylative cyclization of a substrate containing an *E*‐allylic phosphate was not successful and gave only the alkyne hydroarylation product as a 2 : 1 mixture of geometric isomers [Eq. (20)]. This experiment demonstrates that the *Z*‐stereochemistry of the allylic phosphate is crucial for cyclization to occur, perhaps because the steric requirements of this particular reaction are better accommodated by a *Z*‐allylic phosphate. However, it should be noted that successful cyclization onto acyclic Michael acceptors containing an *E*‐alkene have been described in other reactions [Eq. (16), Table 12, Eq. (27), Scheme 9A, Eq. (29), and Table 14].[^64^, ^70^, ^71^]

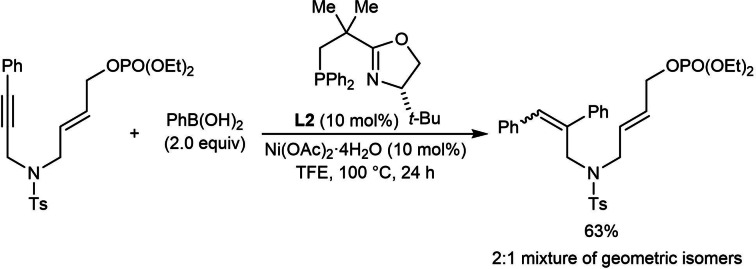




In contrast to previous work,^[65]^ a substrate containing a trimethylsilyl‐substituted alkyne is compatible with the enantioselective arylative cyclization [Eq. (21)]. (*S*)‐*i*‐Pr‐NeoPHOX (**L3**) gave better results than (*S*)‐*t*‐Bu‐NeoPHOX (**L2**), and gave the desired product in 70 % yield and 69 % ee.

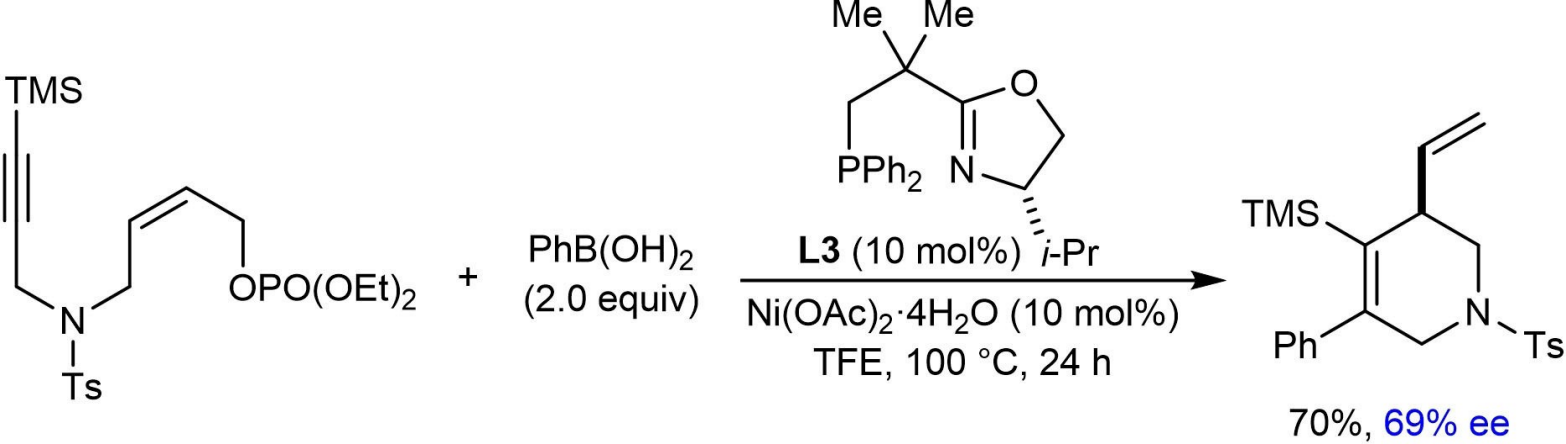




A five‐membered product could also be obtained from a 1,5‐enyne in reasonable yield but with only 42 % ee using *(R)*‐PhPHOX (**L1**) in place of (*S*)‐*t*‐Bu‐NeoPHOX (**L2**) [Eq. 22].

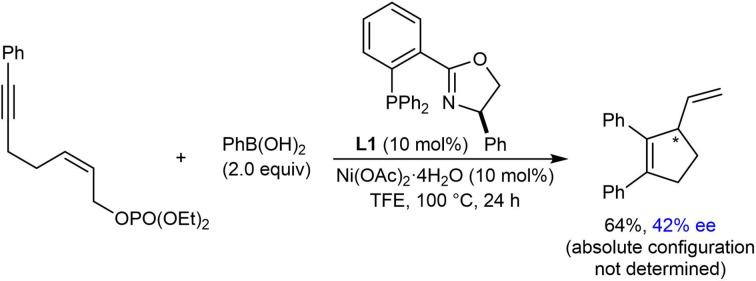




In 2018, the Lam group reported the synthesis of chiral cyclopent‐2‐enones by the enantioselective nickel‐catalyzed desymmetrizing arylative cyclization of alkyne‐tethered malonate esters (Table 8).^[67]^ Bis(2,2,2‐trifluoroethyl) malonates **52** were found to exhibit excellent reactivities in this reaction. Treatment of substrates **52** with a (hetero)arylboronic acid (2.0 equiv.) and 10 mol % each of Ni(OAc)_2_ ⋅ 4H_2_O and (*R*)‐Ph‐PHOX (**L1**) in TFE at 80 °C (Table 8) gave cyclopent‐2‐enones **53** in generally good yields and high enantioselectivities. The reaction is tolerant of substrates containing various groups (R^2^) at the 2‐position, such as 2‐thienyl (**53 a**–**53 c**), 4‐methoxyphenyl (**53 d**, **53 h**, and **53 i**), anilino (**53 e**), 3‐thienylmethoxy (**53 f**) and benzyloxy groups (**53 g**). As well as phenylboronic acid (**53 a** and **53 d**–**53 i**), various other boronic acids can be used such as 3‐bromophenylboronic acid (**53 b**) and 3‐thienylboronic acid (**53 c**). Regarding the alkynyl substituents, phenyl (**53 a**–**53 g**), alkenyl (**53 h**), and 2‐thienyl (**53 i**) groups are tolerated.

**Table 8:**
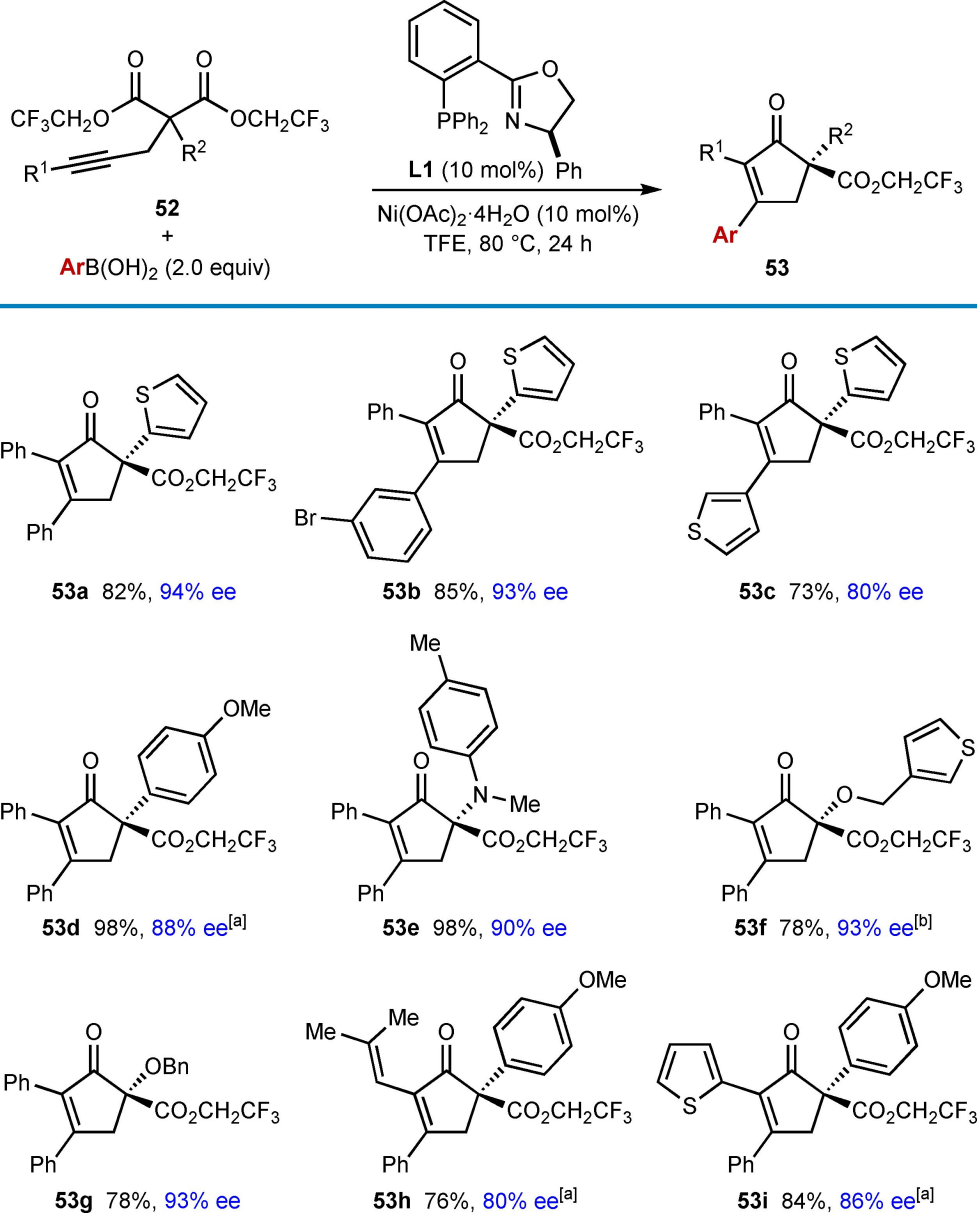
Enantioselective *anti*‐arylmetallative desymmetrizing cyclizations onto malonate esters.

[a] Conducted at 100 °C. [b] Using 20 mol % each of Ni(OAc)_2_ ⋅ 4H_2_O and **L1**.

The reaction of substrates containing a methyl or benzyl substituent at the 2‐position led to moderate yields and poor enantioselectivities when using (*R*)‐PhPHOX (**L1**) as the ligand; however, switching to (*S*)‐*t*‐Bu‐NeoPHOX (**L2**) gave improved but still modest ee values [Eqs. (23) and (24), respectively].

The arylative cyclization of a substrate **54** containing an alkyne tethered to a phenyl ester was also described using (*S*)‐*i*‐ Pr‐NeoPHOX (**L3**) as a ligand, and this gave a 27 : 1 inseparable mixture of the desired product **55** and minor product **56** in 68 % yield [Eq. 25].

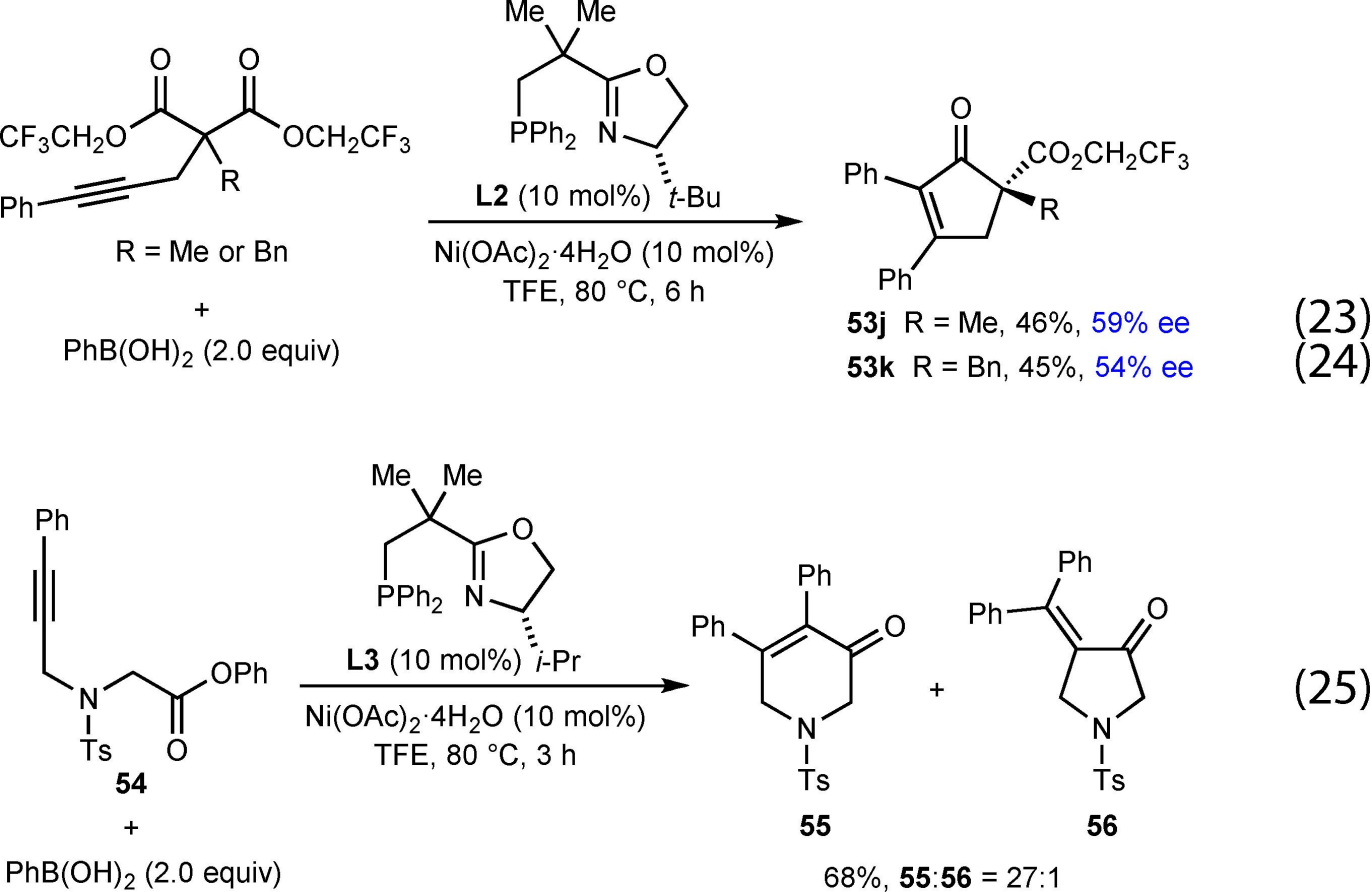




In 2020, Liu and co‐workers reported the enantioselective nickel‐catalyzed *anti*‐carbometallative desymmetrization of malononitriles to give cyclic enones with a nitrile‐containing all‐carbon quaternary center (Table 9).^[68]^ The reactions were conducted by treatment of malononitriles that are tethered to (hetero)aryl alkynes with a (hetero)arylboronic acid (2.0 equiv.), (*S*)‐*t*‐Bu‐PHOX (**L4**, 12 mol %), Ni(OTf)_2_ (10 mol %), and H_2_O (4.0 equiv.) in toluene at 80 °C. Changing the substituent at the α‐position of the malononitrile from a benzyl group (**60 a**–**60 d**) to allyl (**60 e**), 3‐oxobutyl (**60 f**), methyl (**60 g**), or phenyl groups (**60 h**) was tolerated. Various boronic acids can be used in this reaction, including phenylboronic acid (**60 a**–**60 c**, **60 e**–**60 h** ), 3‐furylboronic acid (**60 i**), and 4‐substituted phenylboronic acids with formyl (**60 j**) or vinyl (**60 k**) groups. 4‐Carboxyphenylboronic acid, 4‐aminocarbonylphenylboronic acid, 3‐pyridylboronic acid, unprotected 5‐indolylboronic acid, 2‐methoxycarbonylphenylboronic acid, and (*E*)‐phenylvinylboronic acid did not react successfully. Cyclopentenone **60 l** and seven‐membered imine **61** were obtained by shortening or extending the carbon tether of the substrate, respectively. Interestingly, seven‐membered imines are stable enough to be isolated by column chromatography; however, they are readily hydrolyzed to the corresponding ketone by treatment with 3 M HCl at 0 °C. The arylative cyclization of a malononitrile containing a 2‐pyridyl‐substituted alkyne was also attempted but none of the product **60 d** was observed. The reaction of a methylalkyne‐containing malononitrile gave a mixture of isomers **60 m** and **62**, likely because of poor regioselectivity in the migratory insertion of the alkyne into the arylnickel species as discussed in Section 2.

**Table 9:**
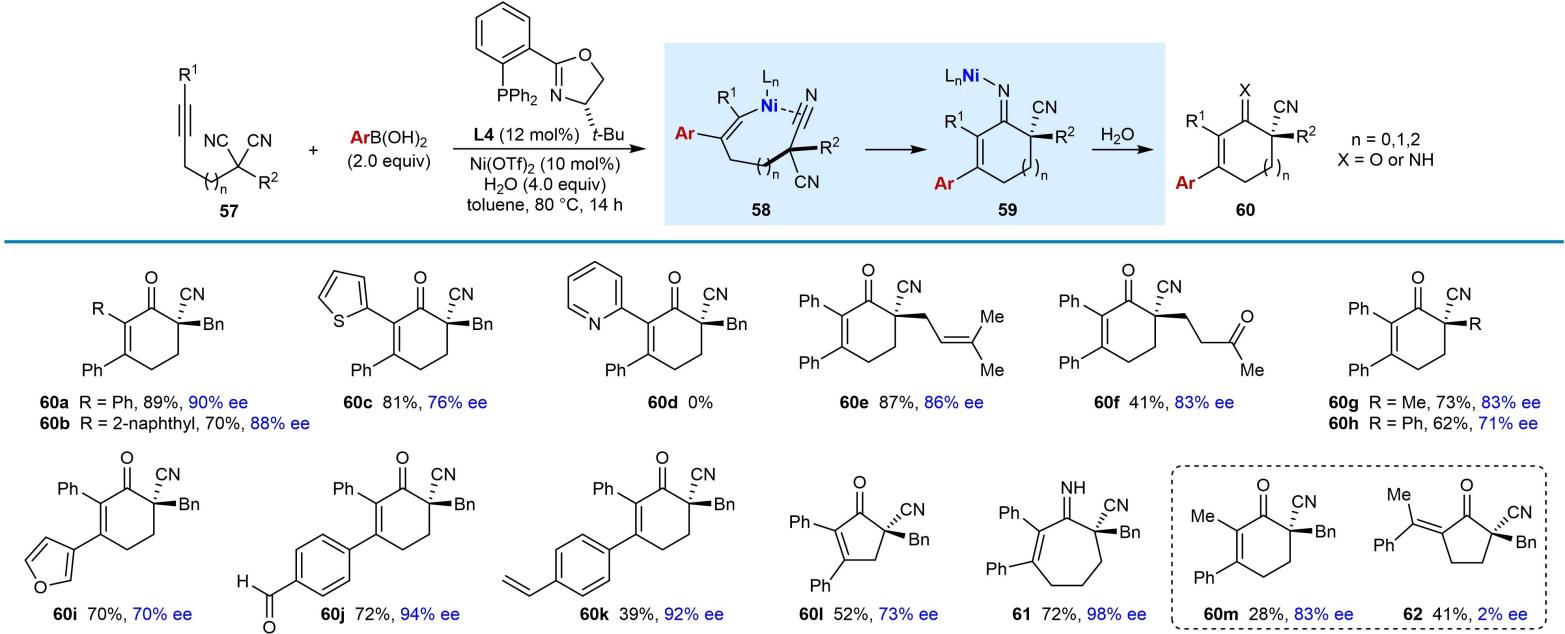
Enantioselective *anti*‐arylmetallative desymmetrizing cyclizations onto malononitriles.

The proposed mechanism is analogous to that shown in Scheme 2A where in this case cyclization of the alkenynickel intermediate occurs onto one of the nitrile groups (as in **58**, Table 9). Following cyclization, protonation of **59** initially gives an imine that, with the exception of the reaction producing **61**, undergoes hydrolysis to the ketone in situ. Competition experiments revealed that electron‐rich arylboronic acids react slightly faster than electron‐poor arylboronic acids. Also, electron‐rich aryl alkynes react significantly faster than electron‐poor aryl alkynes. ^13^C kinetic isotopic effect (KIE) experiments of a substrate at natural abundance revealed a significant ^13^C KIE for the nitrile carbon, suggesting that the addition to the nitrile (**58** to **59**) is likely the rate‐determining step (RDS); however, the transmetalation step cannot be ruled out as the RDS. Finally, ^31^P NMR studies suggested that water aids the transmetalation step.

Various phenylboron sources were investigated in the reaction with alkyne‐tethered malononitrile **57 a**, and triphenylboroxine and potassium phenyltrifluoroborate performed comparably to phenylboronic acid with respect to both yield and enantioselectivity (Table 10). The use of phenylboronic acid pinacol ester did not provide the desired product, perhaps due to slow transmetalation under base‐free conditions.[^101^, ^102^]

**Table 10:**
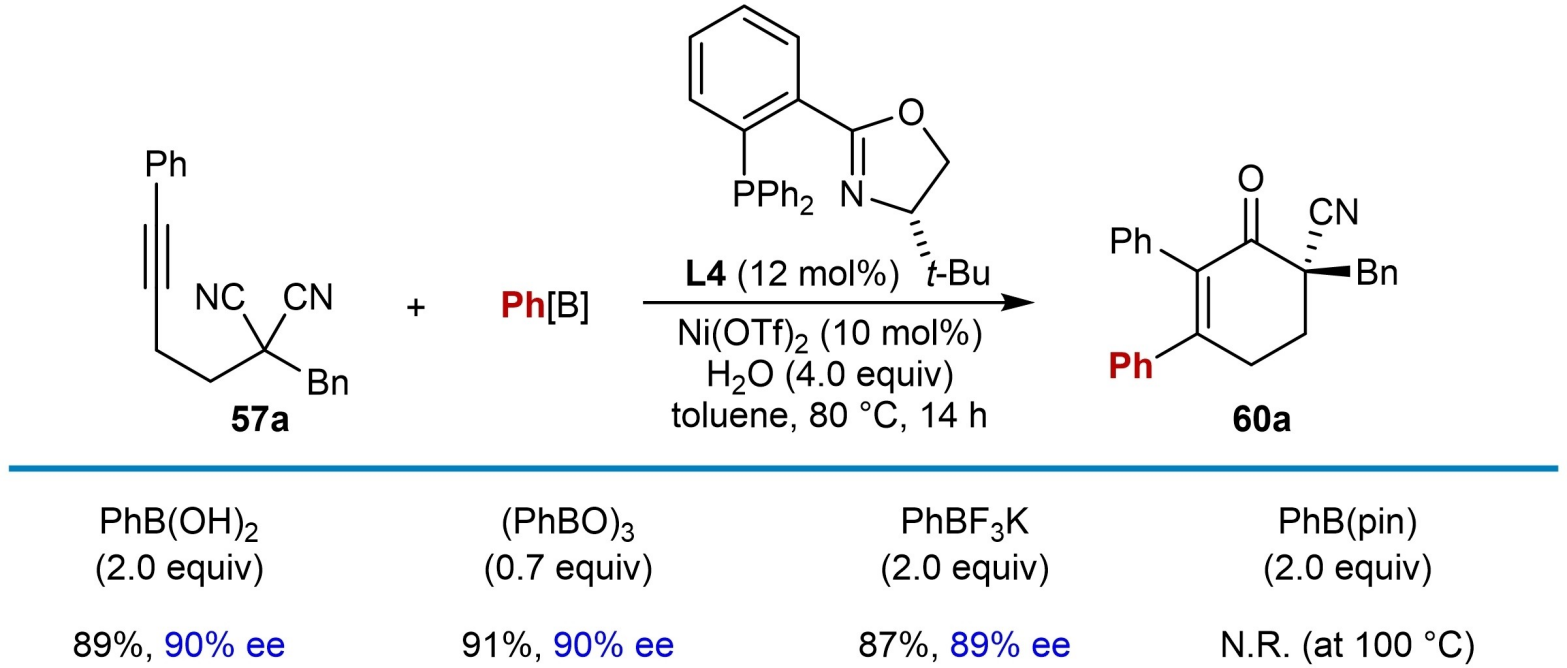
Investigation of various arylboron reagents.

As discussed previously (Tables 4 and 5), the use of cyclic 1,3‐diketones as electrophiles in enantioselective nickel‐catalyzed arylative cyclizations was reported by the Lam group.^[65]^ However, until recently, less reactive acyclic ketones had not been investigated in enantioselective reactions, though Reddy and co‐workers did report two non‐enantioselective examples [Eqs. (14) and (15)].^[64]^ In 2021, the Lam group reported that substrates **63** containing an alkyne tethered to an acyclic ketone can indeed be employed in enantioselective cyclizations, when using a (hetero)arylboronic acid (2.0 equiv.), (*S*)‐*t*‐Bu‐PHOX (**L4**, 10 mol %), and Ni(OAc)_2_ ⋅ 4H_2_O (10 mol %) in TFE at 60 °C, to give products **64** (Table 11).^[69]^ Similar to previous examples,[^65^, ^66^] small quantities of minor arylative cyclization products **65** were formed resulting from the arylnickel species adding across the alkyne with the opposite regioselectivity. The reaction tolerates a range of substituents at the ketone including methyl (**64 a**, **64 e**, and **64 g**–**64 j**), ethyl (**64 f**), *i*‐propyl (**64 b**), 3‐(trifluoromethyl)phenyl, (**64 c**) and 3‐methoxy‐3‐oxopropyl groups (**64 d**). Various alkynyl substituents are tolerated including phenyl (**64 a**–**64 d** and **64 h**–**64 j**), 4‐carbomethoxyphenyl (**64 e**), and vinyl (**64 f**), although the enantioselectivity was only 45 % ee in the latter case. Interestingly, a substrate containing a chloroalkyne was successful in providing product **64 g**; however, the yield was only 12 % and the enantioselectivity was modest (71 % ee). A substrate containing a 4‐nitrophenylsulfonamide was used to investigate the scope of the boronic acid. 4‐(Trimethylsilyl)phenylboronic acid (**64 h**), 3‐hydroxyphenylboronic acid (**64 i**), and 3‐thienylboronic acid (**64 j**) worked well, and good yields and excellent enantioselectivities were observed.

**Table 11:**
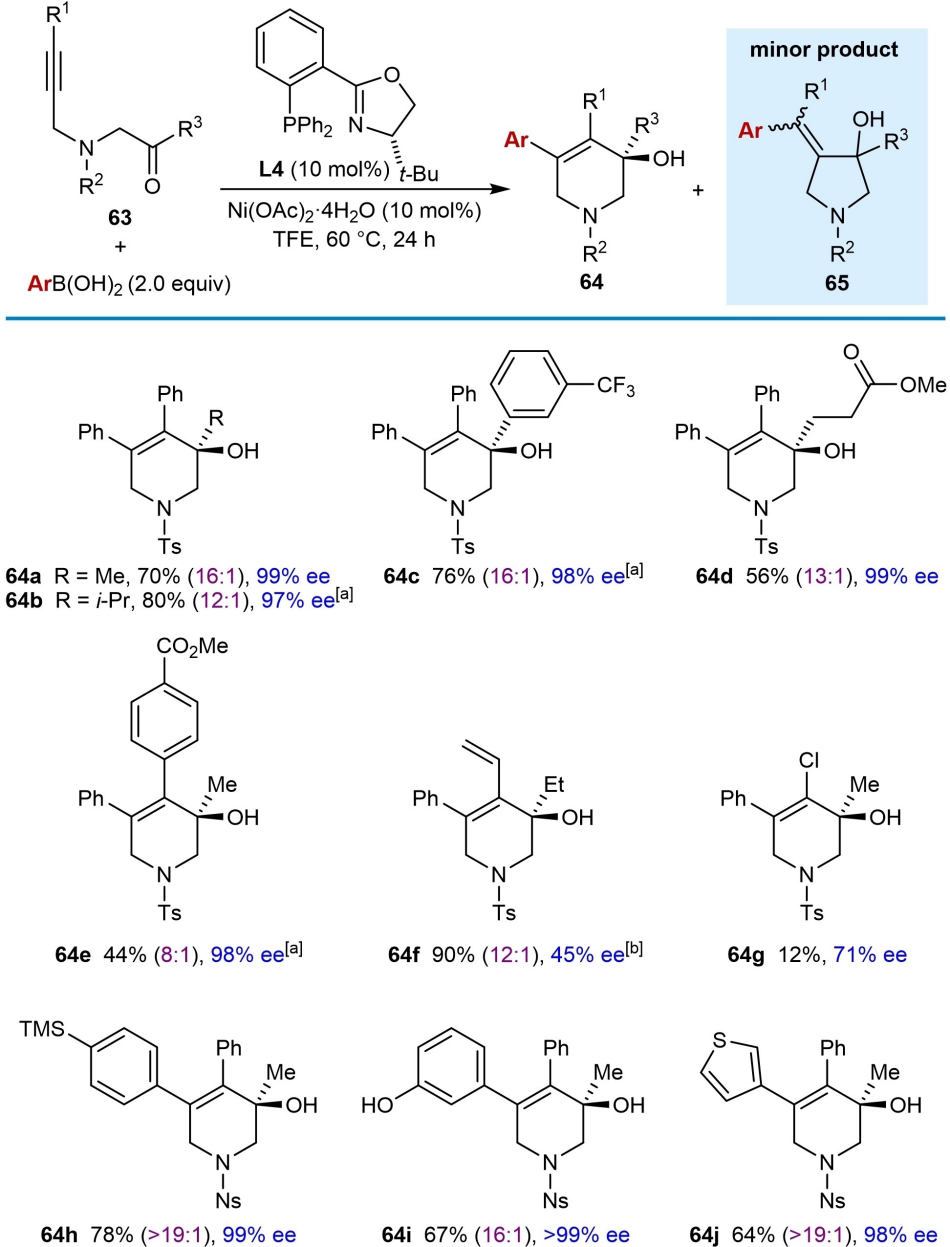
Enantioselective *anti*‐arylmetallative cyclizations onto acyclic ketones.

Unless otherwise stated, yields are of isolated products **64**, free from the minor isomers **65**. [a] Conducted at 80 °C. [b] Product **64 f** was obtained as an inseparable 12 : 1 mixture together with the minor product **65 f** in 90 % combined yield.

To prepare a carbocyclic product **67**, the reaction of alkyne‐tethered ketone **66** was conducted and the desired product was obtained in 25 % yield with 84 % ee [(Eq. (26)]. However, a second product **68** was obtained in 14 % yield and 85 % ee, which resulted from a desymmetrizing cyclization of the intermediate alkenylnickel species onto one of the ester groups.^[67]^ Attempts at shortening and extending the tether to obtain five‐ or seven‐membered products were unsuccessful.

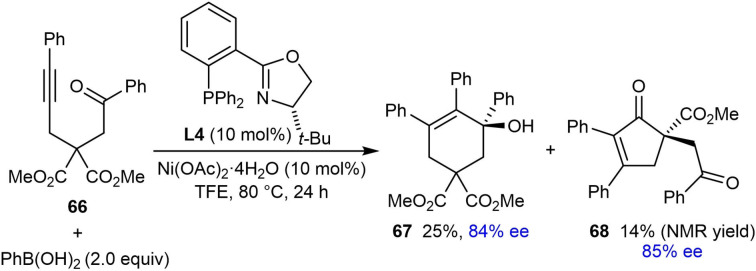




Nickel‐catalyzed arylative cyclizations onto electron‐deficient alkenes to give enantioenriched cyclopentenes were reported by the Lam group (Table 12).^[70]^ Successful arylative cyclizations were achieved in high enantioselectivities when heating the substrates **69** with aryl‐ or alkenylboronic acids (1.2 equiv.), (*S*)‐*t*‐Bu‐NeoPHOX (**L2**, 5 mol %), and Ni(OAc)_2_ ⋅ 4H_2_O (5 mol %) in TFE at 100 °C for 16–18 h. Regarding the electron‐deficient alkene, the reaction tolerates α,β‐unsaturated ketones with a range of substituents at the ketone, including methyl (**70 a**, **70 f**, and **70 g**), chloromethyl (**70 c**), and various (hetero)aryl groups (**70 d**, **70 e**, and **70 h**). An α,β‐unsaturated aldehyde (**70 b**) and nitroalkenes (**70 i**–**70 l**) are also competent electrophiles. Variation of the substituent on the alkyne showed that as well as phenyl groups (**70 a**–**70 e** and **70 i**–**70 l**), vinyl (**70 f**), and 2‐thienyl (**70 g**) groups are also tolerated; however, slight decreases in enantioselectivity were observed in the latter two cases. A substrate with a methyl‐substituted alkyne led to desired product **70 h** in 92 % ee but only 31 % yield, with the low yield likely resulting from poor regioselectivity in migratory insertion of the alkyne into the phenylnickel species as disussed in Section 2. A range of arylboronic acids worked well in the reaction (product **70 i** is one representative example). Various alkenylboronic acids also reacted to give the desired products **70 j**–**70 l** in high enantioselectivities but in low yields (25–36 %). These results are in contrast with comparable nickel‐catalyzed arylative cyclizations where alkenylboronic acids did not work,[^65^, ^68^, ^71^, ^73^] though it should be noted that an alkenylboronic acid could be used in nickel‐catalyzed intramolecular allylic alkenylations, though also in a low (13 %) yield (product **49 f**, Table 7).^[66]^

**Table 12:**
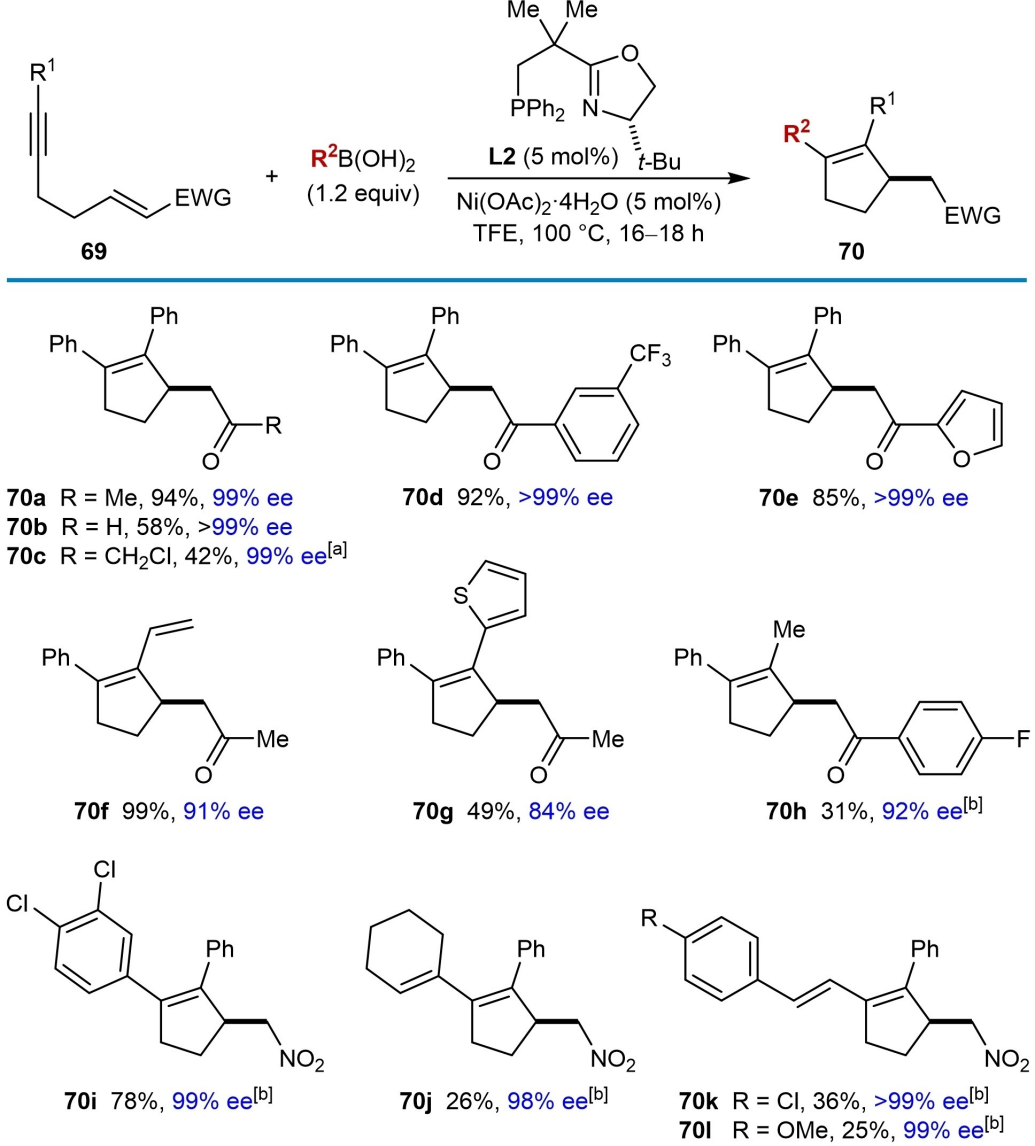
Enantioselective *anti*‐arylmetallative cyclizations onto acyclic electron‐deficient alkenes.

[a] Using 2.0 equiv. of PhB(OH)_2_ and 20 mol % each of Ni(OAc)_2_ ⋅ 4H_2_O and **L2**. [b] Using 2.0 equiv. of boronic acid and 10 mol % each of Ni(OAc)_2_ ⋅ 4H_2_O and **L2**.

Interesting results were obtained with substrates containing α,β‐unsaturated *t*‐butyl ketones [Eq. (27) and (28)]. Arylative cyclization of a substrate with the enone in the *E*‐configuration led to the desired cyclopentene **70 m** in 25 % yield and >99 % ee, along with hydroarylation product **71** in 30 % yield as a 0.7 : 1 inseparable mixture of *E : Z* isomers [(Eq. (27)]. Interestingly, however, the reaction of a stereoisomeric substrate with the enone in the *Z*‐configuration gave cyclopentene **70 m** in 90 % yield and >99 % ee [(Eq. (28)]. The greater propensity of electron‐deficient *Z*‐alkenes to undergo nickel‐catalyzed arylative cyclization compared with their *E*‐configured counterparts was also observed in intramolecular allylic alkenylations reported previously [compare Table 7 and Eq. (20)].^[66]^ The absolute configuration of cyclopentene **70 m** was the same when starting from either the *Z*‐ or *E*‐alkene, which is in contrast to some other enantioselective 1,4‐additions of carbon nucleophiles to electron‐deficient alkenes where *E*‐ and *Z*‐isomers of the substrates provide opposite enantiomers of the products.[^103^, ^104^, ^105^, ^106^, ^107^] However, examples of conjugate additions where *E*‐ and *Z*‐isomers give the same major enantiomers of the products have also been reported.[^30^, ^104^]

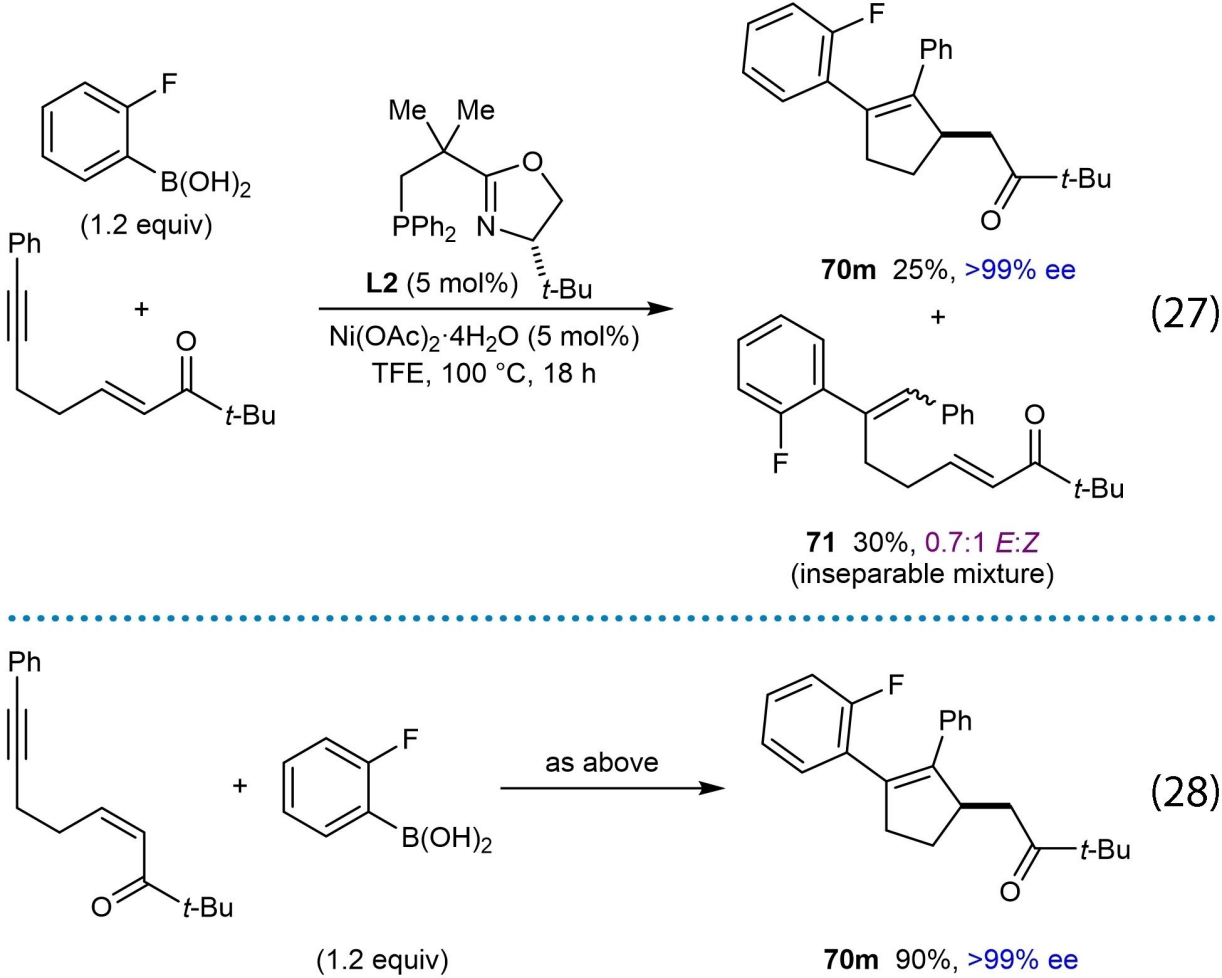




The reaction of phenylboronic acid with a substrate containing an α,β‐unsaturated ester as the electron‐deficient alkene gave some unexpected products. (Scheme 9A). The desired cyclopentene **70 n** was obtained in 14 % yield and >99 % ee, but the conjugated dienes **73** (23 % yield) and **74** (15 % yield), along with the reductive cyclization product **72** (which could not be isolated cleanly) were also formed. A mechanistic rationale for the formation of these products is depicted in Scheme 9B. Initially, addition of a phenylnickel species across the alkyne followed by *E*/*Z* isomerization gives alkenylnickel species **75**. A stereospecific migratory insertion of the alkene into the alkenylnickel species leads to a *C*‐bound nickel enolate **76**, which can undergo protodenickelation to give the cyclopentene **70 n**. However, the low yield of **70 n** (14 %) suggests that this step is slow compared with all the substrates described thus far, which would proceed via ketone enolate or nitronate intermediates. A possible reason for the slower protodenickelation of ester‐derived nickel enolates is that this step proceeds faster via the *O*‐bound, rather than the *C*‐bound enolate (or nitronate), and ester‐derived enolates would be expected to have a higher ratio of *C*‐ vs. *O‐*bound forms compared to ketone‐derived enolates and nitronates. A competing reaction can occur where the nickel enolate **76** can undergo rotation around the C−C bond to give **76′**, followed by *syn*‐β‐hydride elimination to give diene **73** and a nickel hydride **77**. This type of reactivity has been observed previously in nickel‐catalyzed additions of boronic acids to α,β‐unsaturated esters, amides, nitriles, and ketones giving either Mizoroki‐Heck products or 1,4‐addition products by fine‐tuning of the ligand.^[108]^ The nickel hydride **77** can then undergo hydronickelation with the alkyne of the substrate followed by *E*/*Z* isomerization to give alkenylnickel species **78**, which produces the reductive cyclization product **72** and conjugated diene **74** via a sequence of steps analogous to those discussed above. Similar to the examples discussed above [(Eq. (27)) and (28)], arylative cyclization onto an α,β‐unsaturated nitrile was much more successful when the alkene had the *Z*‐configuraton. Attempted arylative cyclization of a substrate with an *E*‐configured α,β‐unsaturated nitrile gave diene (*E*)‐**79** in 18 % yield and a trace of diene **80** [(Eq. (29)]. However, the corresponding reaction of a substrate with a *Z*‐configured α,β‐unsaturated nitrile led to the desired cyclopentene **70 o** in 60 % yield and 99 % ee along with diene (*Z*)‐**79** in 25 % yield [(Eq. (30)]. The formation of the dienes **79** and **80** can be explained by mechanistic pathways similar to those shown in Scheme 9. Interestingly, the dienes (*E*)‐**79** and (*Z*)‐**79** obtained from the reaction of *E*‐ and *Z*‐configured substrates, respectively, are of opposite configuration, presumably because the migratory insertion of the α,β‐unsaturated nitrile into the intermediate alkenylnickel species, and the β‐hydride elimination steps, are both *syn*‐stereospecific.

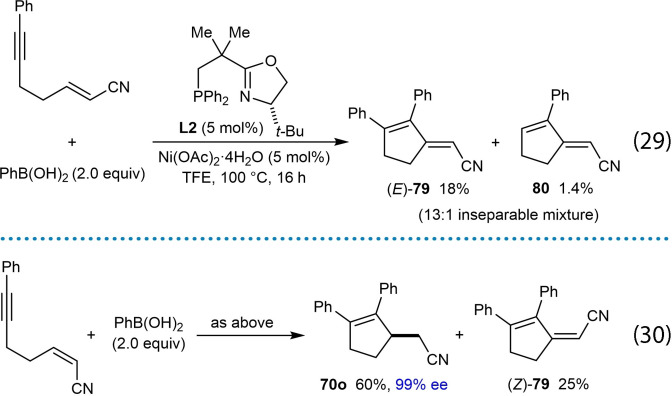




## 5 *Syn*‐Arylmetallative Cyclizations of Alkyne‐Tethered Electrophiles

**Scheme 10:**
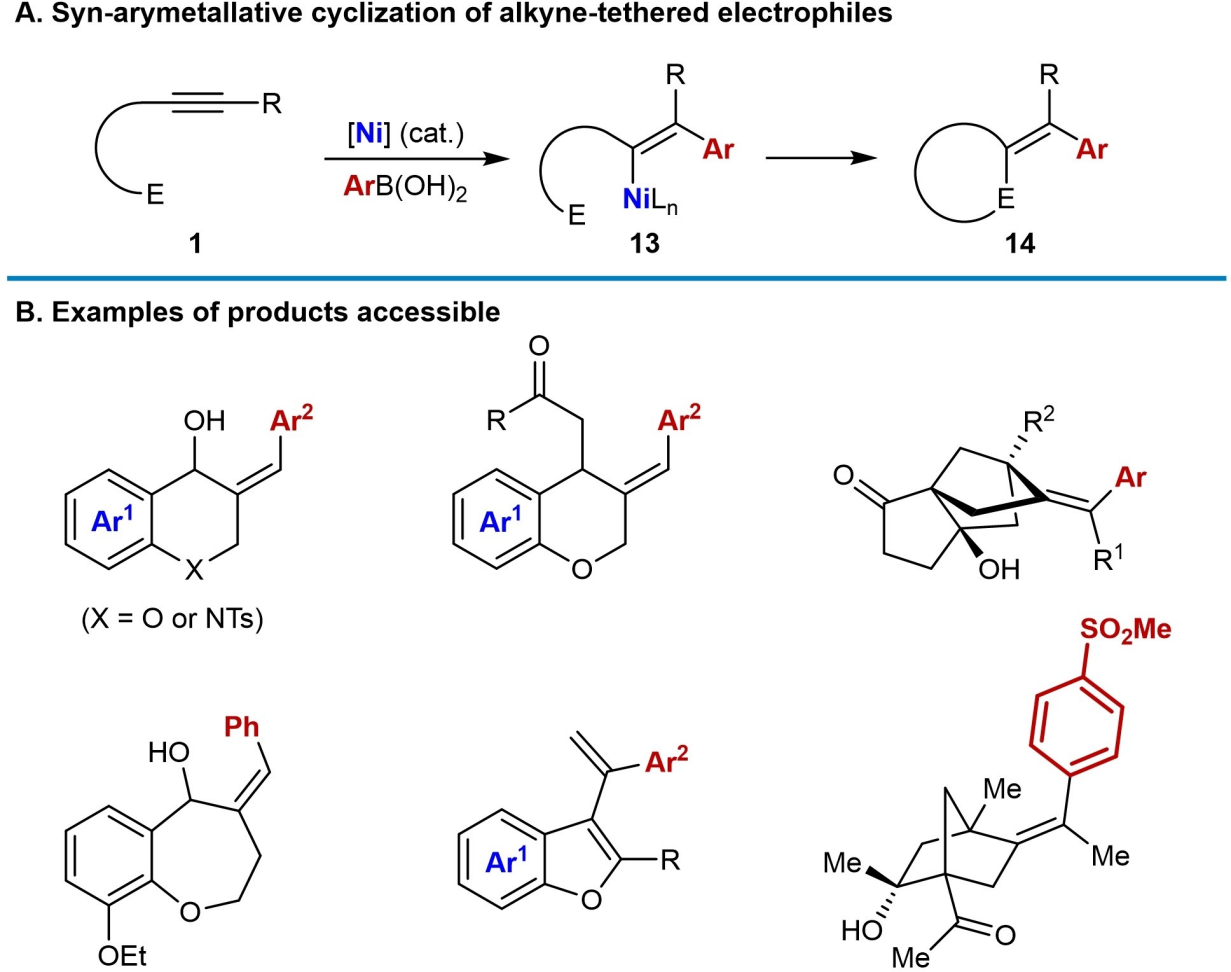
Overview of *syn*‐arylmetallative cyclizations of alkyne‐tethered electrophiles.

Rhodium‐,[^19^, ^20^, ^21^, ^22^, ^23^, ^24^, ^25^, ^26^, ^27^, ^28^, ^29^, ^30^, ^31^] palladium‐,[^32^, ^33^, ^34^, ^35^, ^36^, ^37^, ^38^, ^39^, ^40^, ^41^] or copper‐catalyzed^[42]^ arylative cyclizations of alkyne‐tethered electrophiles involving 1,2‐arylmetallation of the alkyne to place the metal proximal to the electrophile, followed by direct intramolecular trapping of the intermediate alkenylmetal species with the tethered electrophile (see Scheme 1A), have been reported extensively. However, the use of catalyst systems based upon other metals are of interest because this could allow access to new reactivity and different reaction outcomes, with a consequent increase in scope. Recently, nickel has been reported to be effective in such reactions (Scheme 10A). Nickel‐catalyzed arylative cyclizations onto ketones, α,β‐unsaturated ketones, or esters are described in this section, along with more complex domino sequences where cyclization onto an alkene is followed by a second cyclization onto a ketone. These reactions lead to the synthesis of various carbo‐ and heterocyclic products such as chromanes, tetrahydroquinolines, benzoxepines, benzofurans, tricyclo[5.2.1.0^1,5^]decanes, and bicyclo[2.2.1]heptanes (Scheme 10B).

The first example of *syn*‐selective nickel‐catalyzed carbometalation of alkynes using arylboron reagents, followed by cyclization of the resultant alkenylnickel species onto a tethered electrophile, was reported by Reddy and co‐workers in 2018 (Table 13).^[71]^ Treatment of substrates **80**, which contain a terminal alkyne tethered to a ketone, with a (hetero)arylboronic acid (2.0 equiv.), Ni(acac)_2_ (10 mol %), PPh_3_ (10 mol %), and Cs_2_CO_3_ (0.2 equiv.) in 1,4‐dioxane at 90 °C gave various chromane and tetrahydroquinoline products. The proposed mechanism follows the generalized catalytic cycle shown in Scheme 2B. Disubstituted phenylboronic acids with electron‐withdrawing (**81 a**) or electron‐donating groups (**81 b**), as well as 3‐furylboronic acid (**81 c**) are effective in the reaction. However, alkenylboronic acids were found to be unsuitable. Substitution at the aryl moiety of the *o*‐propargyloxy benzaldehyde was also explored and bromo (**81 d**), nitro (**81 e**), and alkoxy (**81 e** and **81 f**) groups are well‐tolerated. The scope of the arylboronic acid was investigated in reactions of 2‐propargylamino benzaldehydes. 1,3‐Benzodioxole‐5‐boronic acid (**81 g**), 3‐nitrophenylboronic acid, (**81 h**) and 4‐cyanophenylboronic acid (**81 i**) are all tolerated. The reaction of 2‐homopropargyloxy benzaldehyde **82** provided benzoxepine **83** in 68 % yield [(Eq. 31].

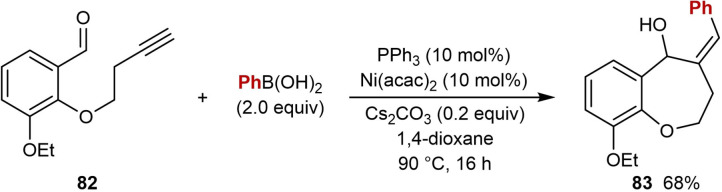




**Table 13:**
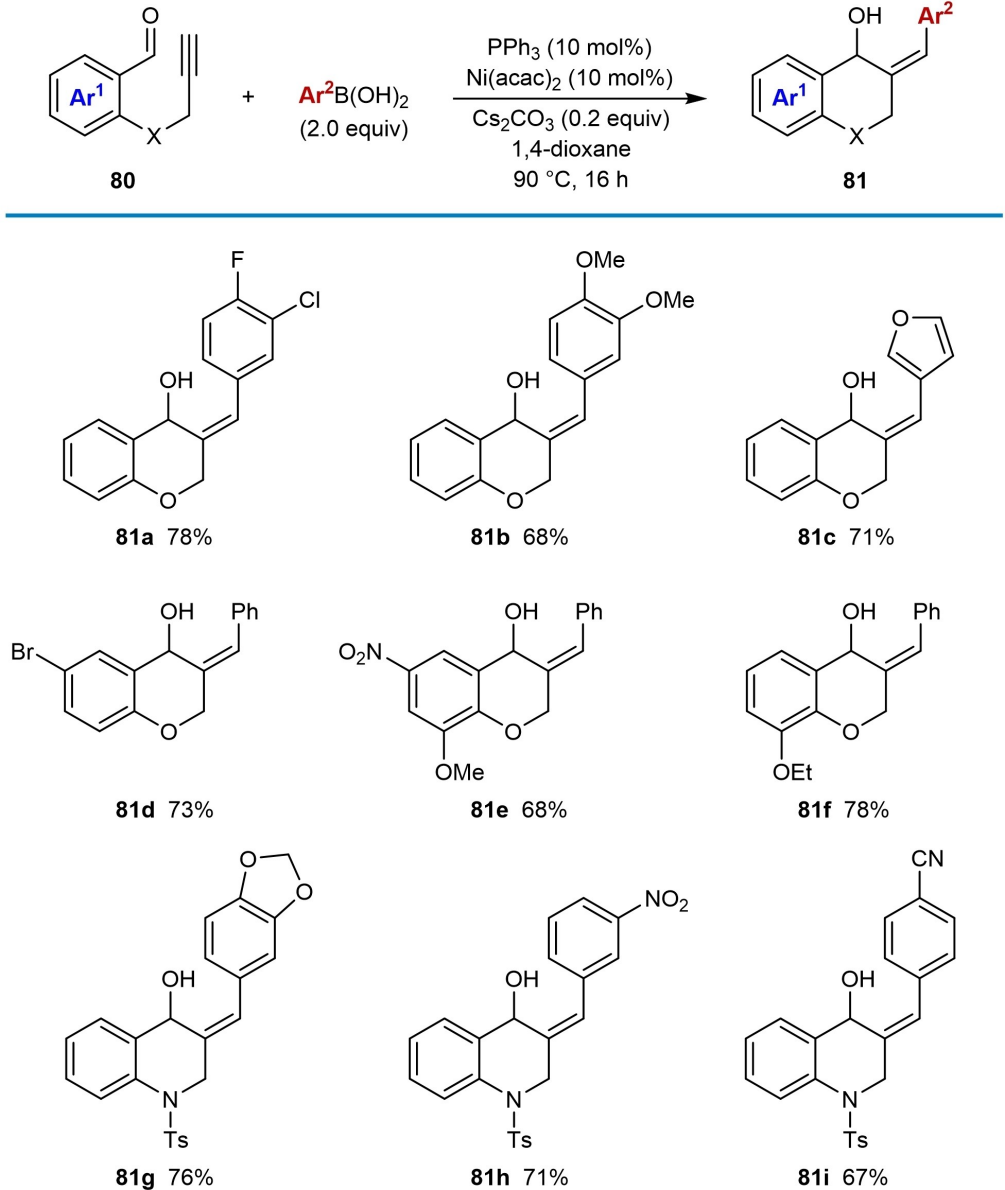
*Syn*‐arylmetallative cyclizations onto aldehydes.

The scope of this process was successfully increased by changing the electrophile from a ketone to an enone (Table 14). The reaction tolerates enones with phenyl (**85 a**) or methyl ketones (**85 b** and **85 c**). A chloride within the benzene tethering moiety is also tolerated (**85 c**). Phenylboronic acid (**85 a** and **85 c**) and 3‐furylboronic acids (**85 b**) were used successfully.

**Table 14:**
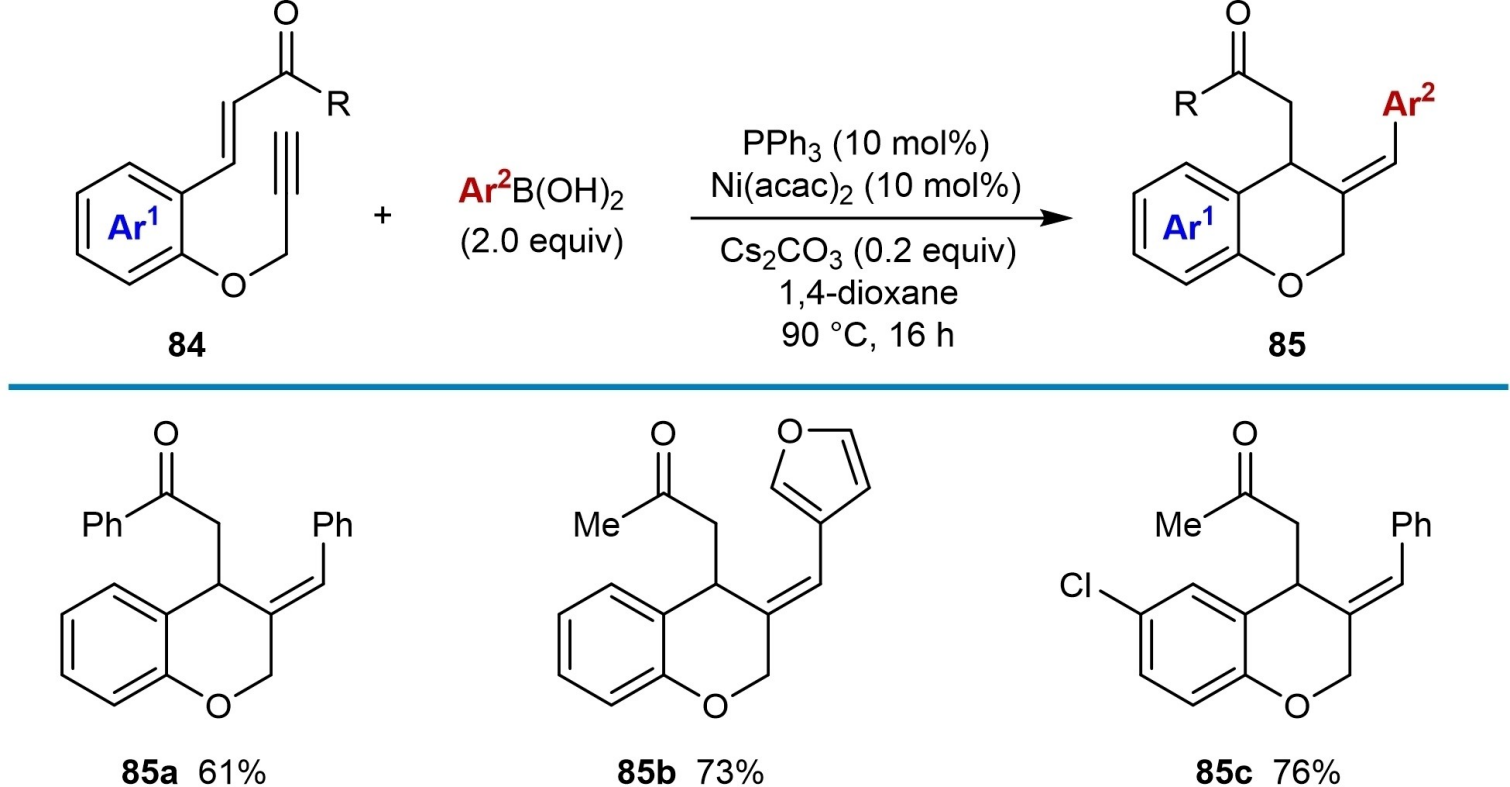
*Syn*‐arylmetallative cyclizations onto enones.

In 2019, Cho and co‐workers reported nickel‐catalyzed arylative cyclizations onto ester electrophiles to give multisubstituted benzofurans **88** (Table 15).^[72]^ The reaction conditions involved heating alkyne‐tethered phenyl esters **86** with (hetero)arylboronic acids (1.5 equiv.), Ni(OAc)_2_ ⋅ 4H_2_O (1–5 mol %), and pyphos (**L5**, 1.2–6 mol %) in TFE at 80 °C. Investigation of the scope of the arylboronic acid revealed that substituents such as a trifluoromethyl group (**88 b**) or a free hydroxyl group (**88 c**) are tolerated. Benzothiophen‐2‐ylboronic acid was also successfully utilized (**88 d**). Exploration of the scope of the alkyne‐tethered phenyl ester showed that electron‐withdrawing groups on the benzene ring are well‐tolerated (**88 e**–**88 g**). Substrates with various primary or secondary alkyl substituents at the acyl group led to products **88 a**–**88 k** in good yields; however, a substrate with a chloroalkyl group gave **88 l** in a lower 33 % yield. The reaction of a benzoyl ester was also successful to give benzofuran **88 m**. The proposed mechanism follows the generalized catalytic cycle shown in Scheme 2B; however, the cyclization step is followed by protonation of the intermediate nickel alkoxide and subsequent elimination of water from the resulting species **87** to give the product.

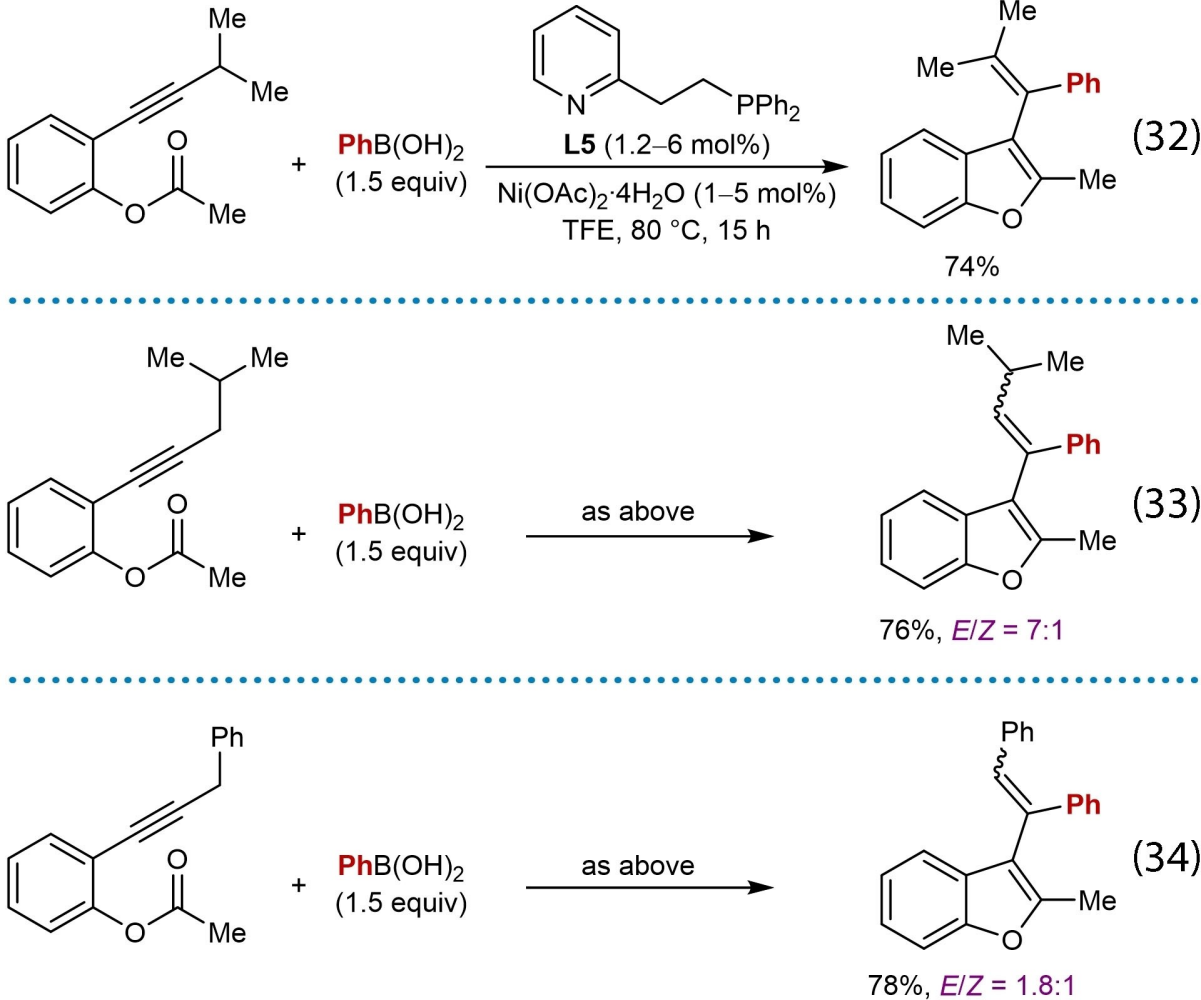




**Table 15:**
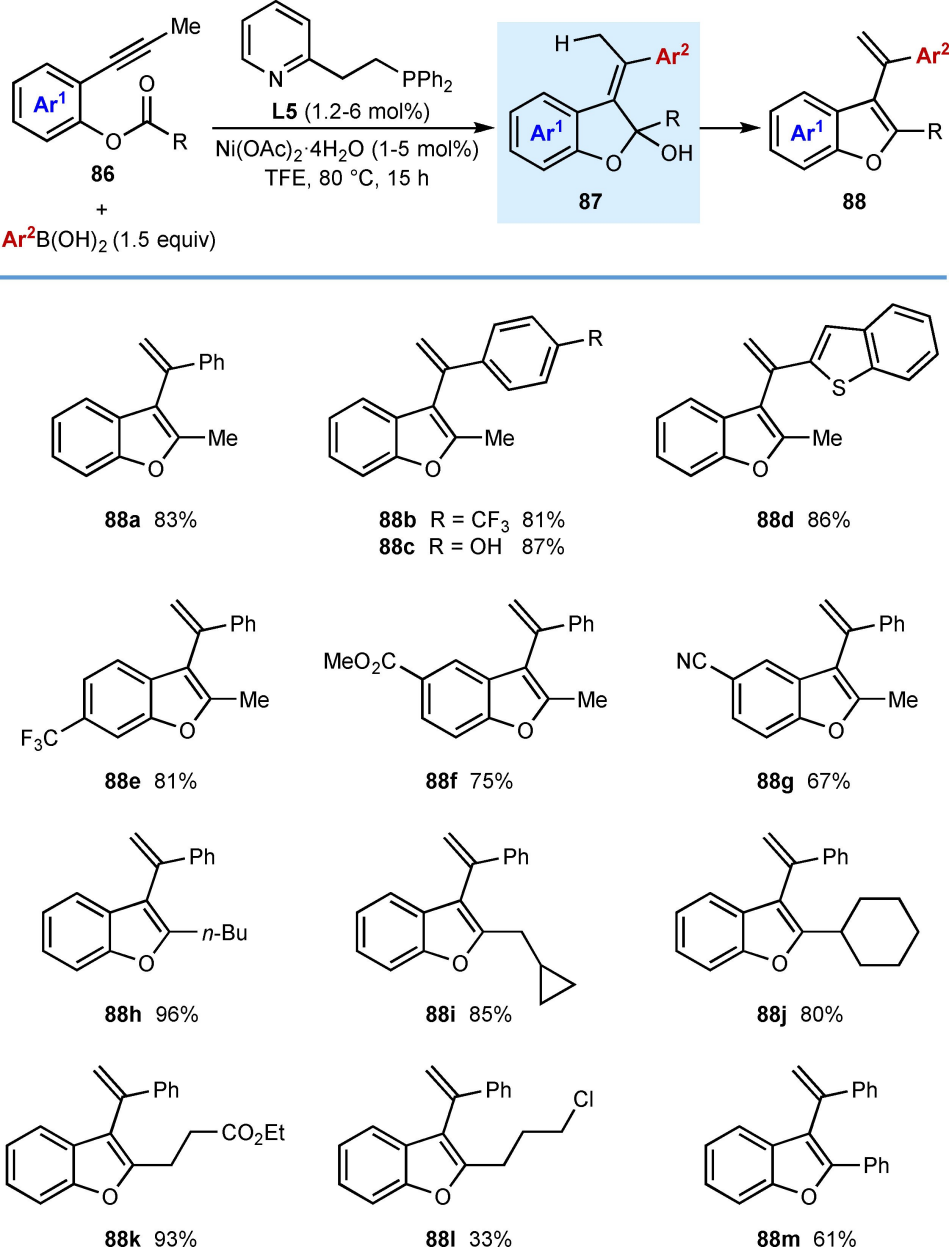
*Syn*‐arylmetallative cyclizations onto esters.

The alkynyl substituent can be changed from a methyl group to isopropyl [Eq. (32)], isobutyl [Eq. (33)], and benzyl [Eq. (34)] groups, though in the latter two cases, the products were isolated as mixtures of *E*/*Z* isomers at the trisubstituted alkene.

In 2020, Kong and co‐workers developed a process that incorporates nickel‐catalyzed difunctionalizations of alkynes into a more complex domino reaction, by using substrates containing three reactive sites in the form of an alkyne, an unactivated alkene, and a cyclic 1,3‐diketone (Table 16).^[73]^ Heating enynones **89** in the presence of a (hetero)arylboronic acid (2.0 equiv.), Ni(OAc)_2_ ⋅ 4H_2_O (10 mol %), and (1*R*,1′*R*,2*S*,2′*S*)‐DuanPhos (**L6**) (12 mol %) in TFE at 100 °C gave complex bridged tricyclo[5.2.1.0^1,5^]decanes **93** with three new carbon‐carbon bonds in high regio‐ and enantioselectivities. The first steps of the proposed mechanism are identical to those depicted in the generalized catalytic cycle in Scheme 2B; however, the alkenylnickel intermediate **90** cyclizes onto the unactivated alkene to give alkylnickel species **91**, which then undergoes cyclization onto one of the ketones to give nickel alkoxide **92**. Finally, protonation of **92** releases the product **93**.

**Table 16:**
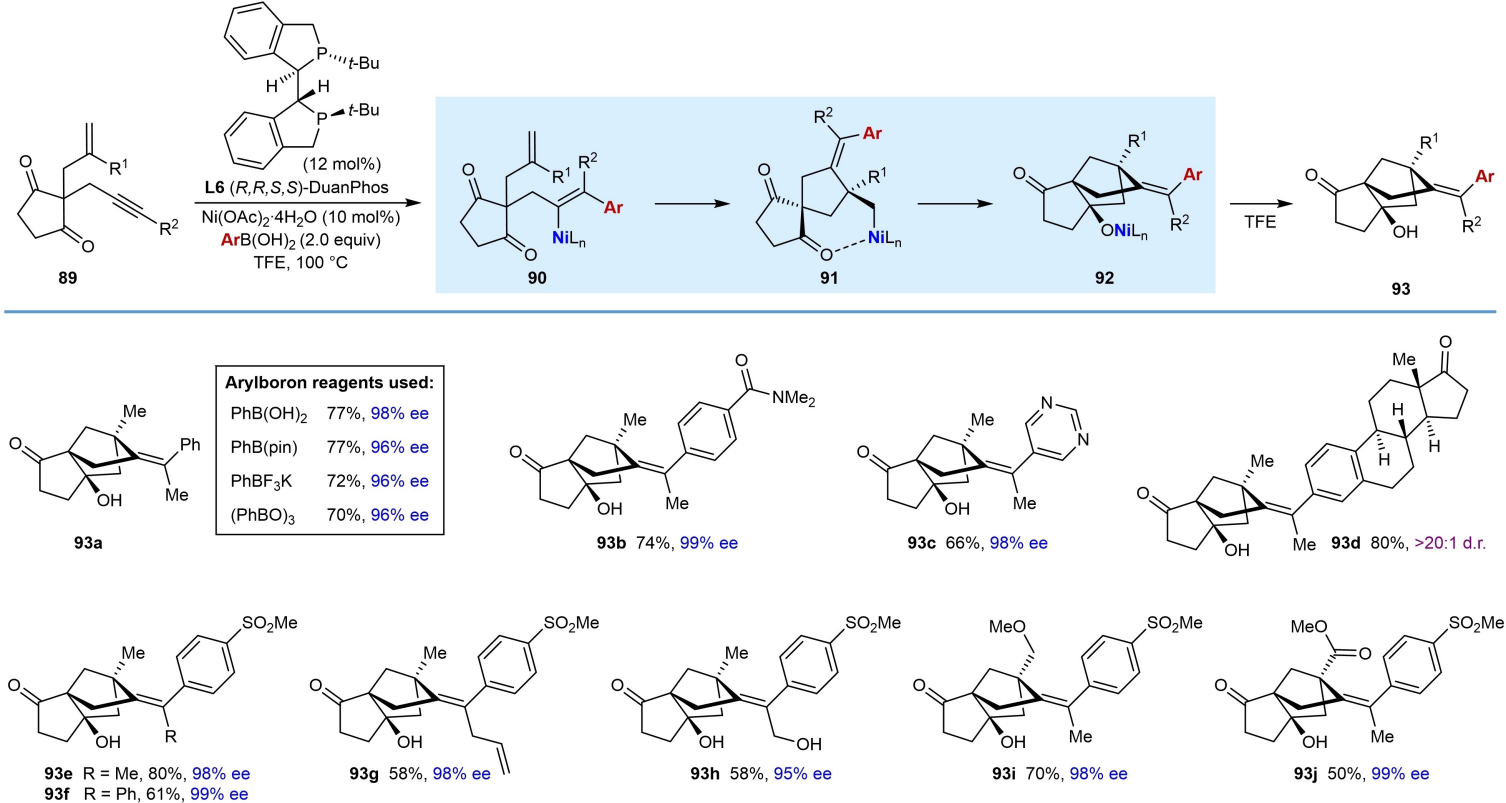
Synthesis of bridged tricyclo[5.2.1.0^1,5^]decanes by enantioselective nickel‐catalyzed asymmetric domino cyclization of enynones.

Various (hetero)arylboronic acids can be used, including phenylboronic acid (**93 a**), an amide‐substituted boronic acid (**93 b**), pyrimidine‐5‐boronic acid (**93 c**), and 4‐(methanesulfonyl)phenylboronic acid (**93 e**–**93 j**). A boronic acid derived from estrone also worked well to give the corresponding product **93 d** in 80 % yield and >20 : 1 diastereomeric ratio. However, alkylboronic acids and alkenylboronic acids failed to give the desired products. The scope of the alkyne substituent was also investigated. In addition to methyl‐substituted alkynes (**93 a**–**93 e**, **93 i**, and **93 j**), alkynes containing allyl (**93 g**) or hydroxymethyl groups (**93 h**) are compatible with the reaction. Interestingly, a phenyl‐substituted alkyne also provided the desired product in a moderate yield (**93 f**, 61 %). Based upon other nickel‐catalyzed arylative cyclizations of substrates containing aryl‐substituted alkynes, and as discussed in Section 2, the phenyl‐substituted alkyne of this substrate might have been expected to undergo migratory insertion with the intermediate arylnickel species with the regioselectivity opposite to that required to form product **93 f**. Therefore, the formation of **93 f** in 61 % yield is notable. It appears likely that the ligand (1*R*,1′*R*,2*S*,2′*S*)‐DuanPhos (**L6**) plays an important role in controlling this regioselectivity. Terminal alkynes are not tolerated in the reaction and provided only complex mixtures of unidentified products. The scope of the alkene substituent was also explored and substrates containing methoxymethyl or ester groups performed well to give products **93 i** and **93 j**, respectively.

Other arylboron reagents such as PhB(pin), PhBF_3_K, and (PhBO)_3_ were also investigated to give **93 a**, and all gave results similar to phenylboronic acid.

**Scheme 11:**
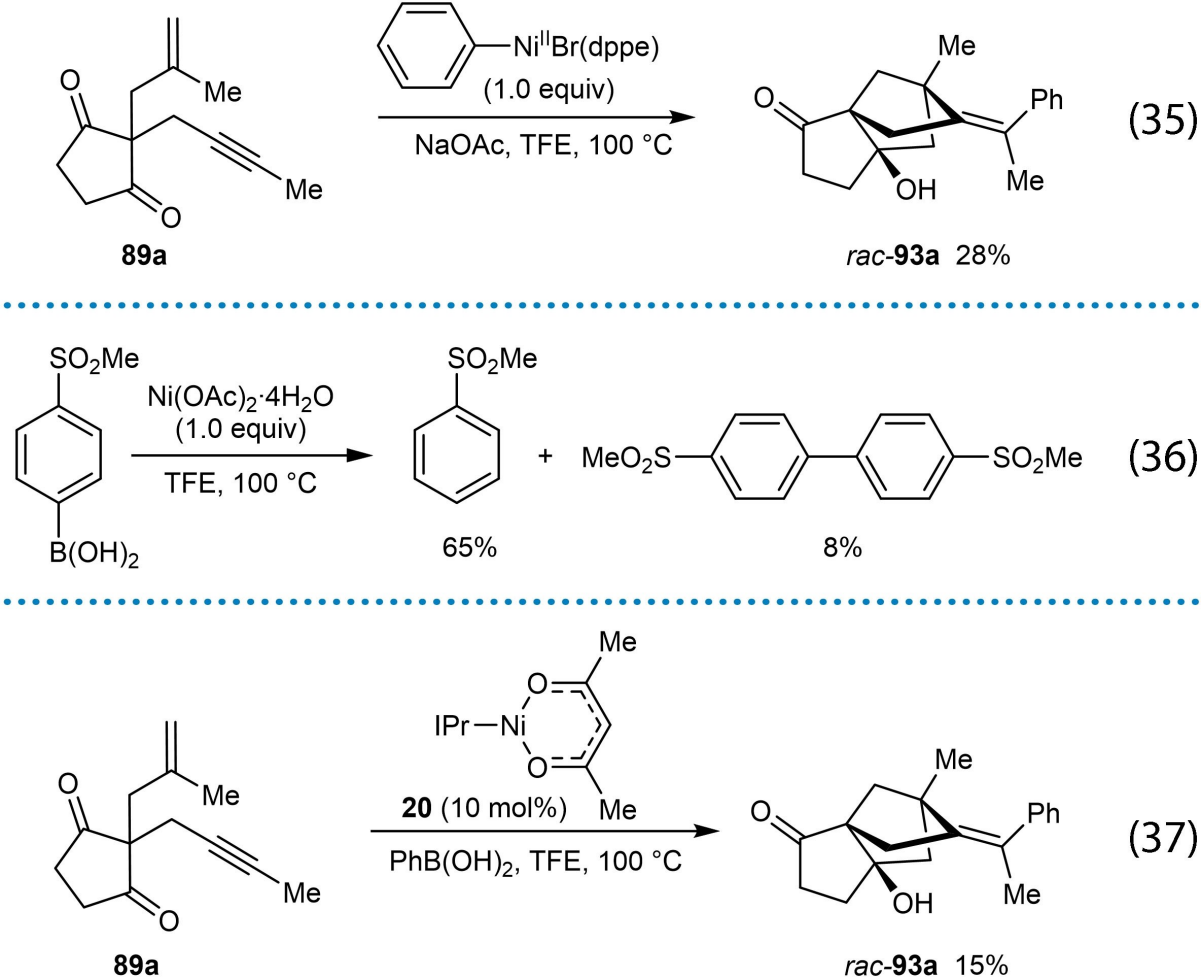
Mechanistic studies.

To gain insight into the oxidation state of the active nickel catalyst, mechanistic studies were carried out (Scheme 11). Reaction of enynone **89 a** with a stoichiometric quantity of PhNiBr(dppe) led to product *rac*‐**93 a** in 28 % yield, suggesting that a Ni(II) species is involved in the catalytic cycle [Eq. (35)]. However, similar to the report by the Liu group on the synthesis of 1‐naphthylamines by nickel‐catalyzed arylative cyclizations [Eq. (1)],^[61]^ a biaryl species was observed in the stoichiometric reaction of 4‐(methylsulfonyl)phenylboronic acid with Ni(OAc)_2_ ⋅ 4H_2_O [Eq. (36)], and as discussed previously,^[61]^ this could indicate the formation of a Ni(I) species. Therefore, the reaction of enynone **89 a** with Ni(I) species **20** was performed, which gave *rac*‐**93 a** in 15 % yield [Eq. (37)], suggesting that a catalytic cycle involving arylnickel(I) intermediates is also viable.

During optimization of the process to produce bridged tricyclo[5.2.1.0^1,5^]decanes, Kong and co‐workers observed that the reaction of methyl‐substituted enynone **89 a** with PhB(OH)_2_, Ni(OAc)_2_ ⋅ 4H_2_O, and (*S*)‐Ph‐PHOX (*ent*‐**L1**) in MeCN led to only a trace amount of the desired product **93 a** [Eq. (38)].^[73]^ Instead, product **94**, resulting from *anti*‐arylmetallative cyclization as also described by the Lam group (Table 4),^[65]^ was formed in 60 % yield. It appears the chiral ligand used; either (*R,R,S,S*)‐DuanPhos (**L6**) or (*S*)‐Ph‐PHOX (*ent*‐**L1**) has a significant impact on the reaction outcome. The formation of **94** rather than **93 a** stems from migratory insertion of the alkyne into the arylnickel intermediate occurring with the opposite regioselectivity, and the fact that **94** is obtained in 60 % yield is interesting because alkyne‐tethered electrophiles containing two alkyl substituents are generally less effective substrates for nickel‐catalyzed *anti*‐arylmetallative cyclizations. Usually, the formation of complex mixtures or significantly reduced yields are observed in other studies using this type of substrate.[^62^, ^63^, ^64^, ^65^, ^66^]

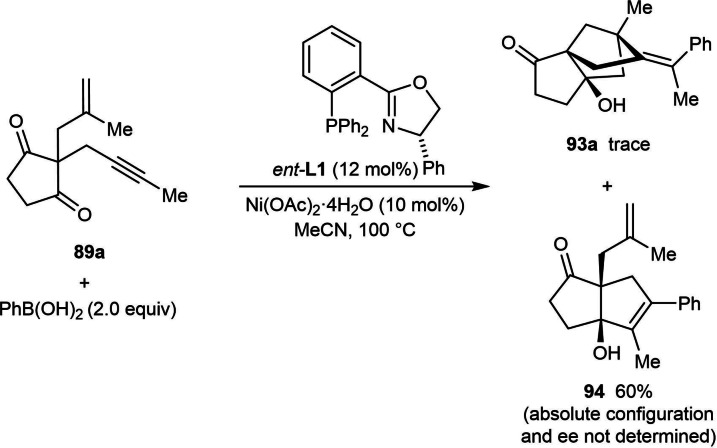




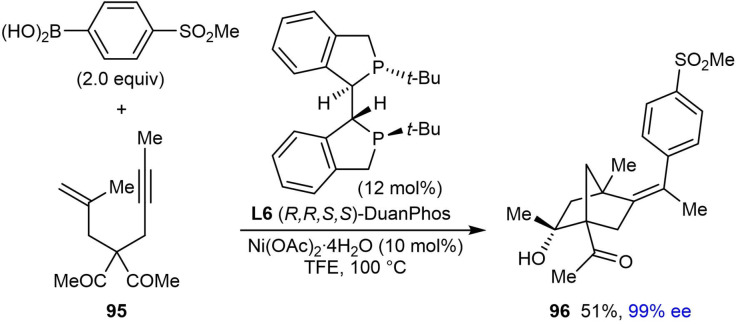




Under the standard conditions, the reaction of substrate **95**, which contains an acyclic 1,3‐diketone, gave highly functionalized bicyclo[2.2.1]heptane **96** in 51 % yield and 99 % ee [(Eq. (39)].

The arylative cyclization reaction of substrate **97**, which contains an allene in place of an alkene, was also successful using dppe as an achiral ligand to give *rac‐* **98** in a moderate 35 % yield [(Eq. 40].

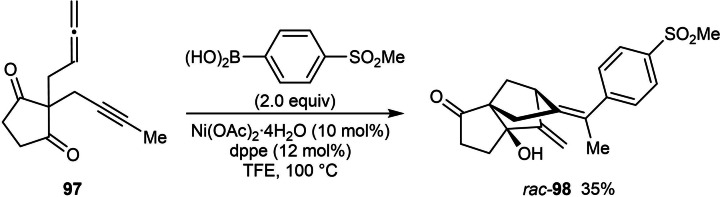




## 6 Enantioselective Arylative Cyclizations of Allene‐Tethered Electrophiles

Intermolecular nickel‐catalyzed additions of arylboron reagents examples to allenes have been reported,[^109^, ^110^, ^111^] where the intermediate allylnickel species are either protonated^[111]^ or engage in nucleophilic attack of an aldehyde.[^109^, ^110^] However, at the time of writing, only two reports of nickel‐catalyzed arylative cyclization of allene‐tethered electrophiles involving an arylboron reagent have been reported. In these studies, cyclohexa‐2,5‐dienones or ketones were used as electrophiles, leading to the synthesis of various carbo‐ and heterocyclic products such as hexahydroindol‐5‐ones, hexahydrobenzofuran‐5‐ones, pyrrolidine‐2‐ones, pyrrolidines, cyclopentanes, and piperidines in high diastereo‐ and enantioselectivities.

The first enantioselective nickel‐catalyzed arylative cyclizations of allene‐tethered electrophiles were reported by the Lam group in 2018 (Table 17).^[74]^ Treatment of substrates containing an allene tethered to a cyclohexa‐2,5‐dienone with (hetero)arylboronic acids (2.0 equiv.), Ni(OAc)_2_ ⋅ 4H_2_O (10 mol %), and (*R*)‐Ph‐PHOX (**L1**) in a 3 : 2 mixture of MeCN/1,4‐dioxane at 80 °C gave hexahydroindol‐5‐ones and hexahydrobenzofuran‐5‐ones with three contiguous stereocenters in high diastereo‐ and enantioselectivities. The reactions are successful using both *N*‐sulfonyl‐tethered and *O*‐tethered substrates leading to 6,5‐bicycles **100**. Methyl (**100 e**–**100 j**), ethyl (**100 a** and **100 b**), or phenyl (**100 c**) groups at the quaternary center of substrate **99** are tolerated. The reaction of a substrate containing a longer tether was largely unsuccessful and provided only a trace of the 6,6‐bicycle **100 d**. The reaction tolerates various substituted phenylboronic acids with substituents such as vinyl (**100 e**), acetoxy (**100 h**), and chloro (**100 i**) groups. A disubstituted phenylboronic acid (**100 f**), 2‐naphthylboronic acid (**100 g**), and 3‐furylboronic acid (**100 j**) were also successful in the reactions. The proposed mechanism for these reactions is analogous to that shown in Scheme 2C.

**Table 17:**
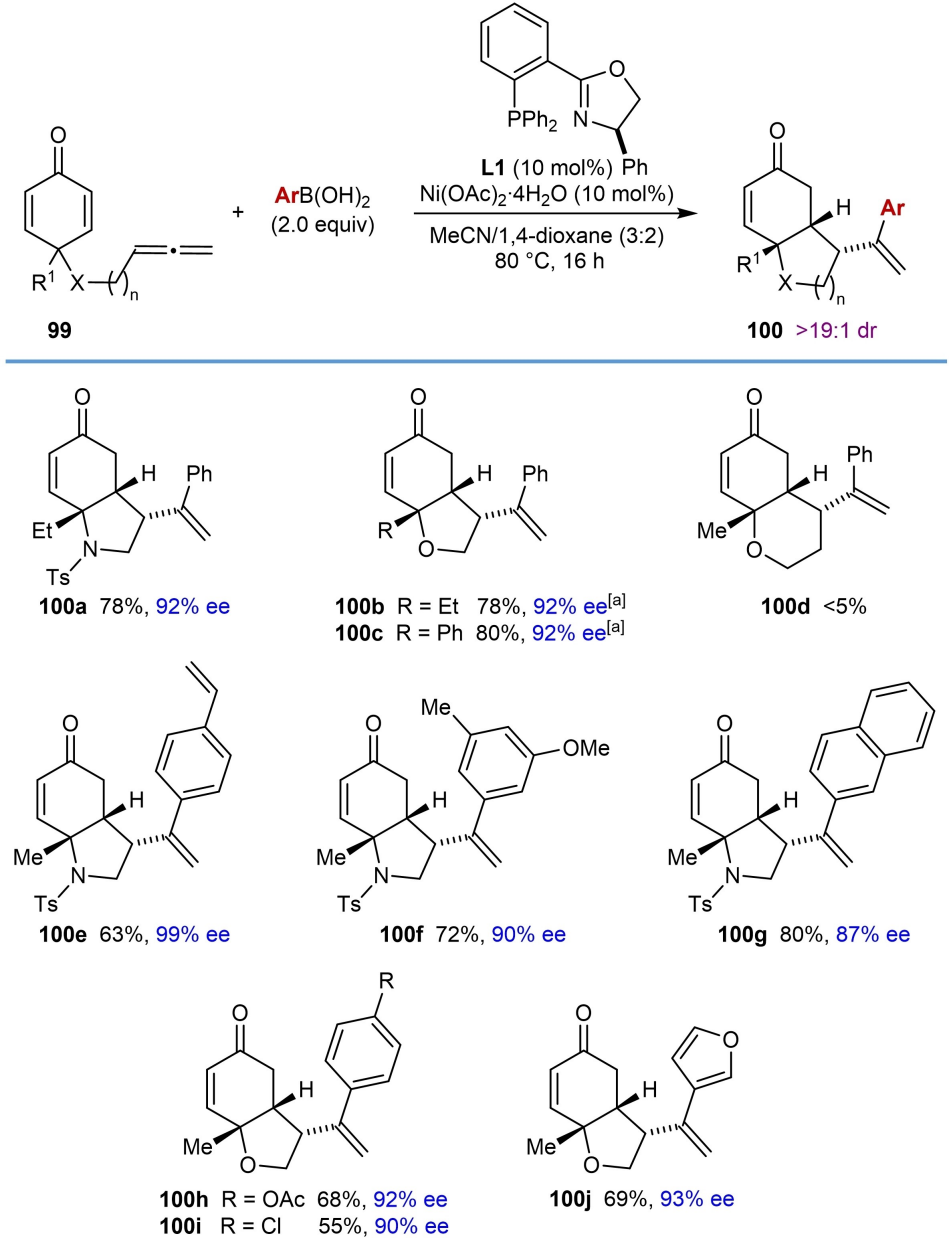
Enantioselective nickel‐catalyzed arylative intramolecular 1,4‐allylations of cyclohexa‐2,5‐dienones.

[a] Isolated together with cyclobutane side products as inseparable mixtures in ratios of between 17 : 1 and 20 : 1. Yields have been adjusted accordingly.

**Scheme 12:**
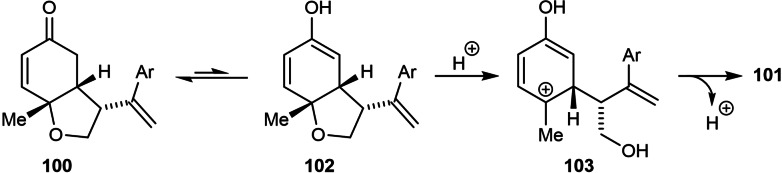
Rationale for phenol formation.

The use of certain substituted phenylboronic acids containing strongly electron‐withdrawing substituents led to the unexpected formation of 3,4‐disubstituted phenols **101** in addition to the desired products **100**, with both products obtained in high enantioselectivities (Table 18). It was suggested that the formation of phenol **101** is the result of enolization of the ketone to give **102**, which then undergoes ring‐opening of the furan ring to give **103** (Scheme 12). Presumably, this step is promoted by a Brønsted acid or a hydrogen bond donor. Finally, proton loss from **103** leads to phenol **101**.

**Table 18:**
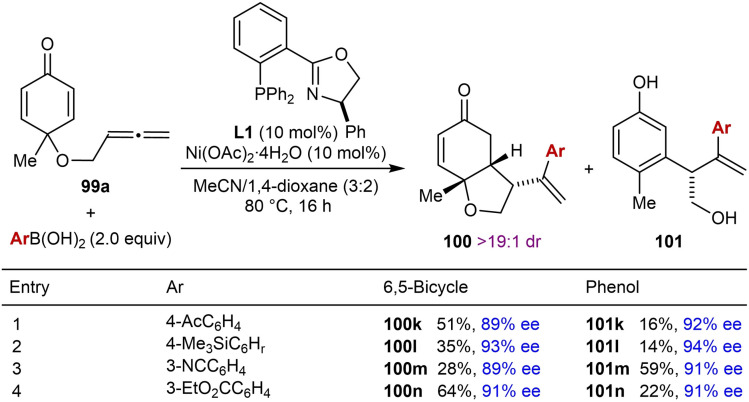
Reactions producing phenols.

The nickel‐catalyzed arylative and alkenylative 1,2‐allylation of allene‐tethered ketones to give enantioenriched tertiary‐alcohol‐containing pyrrolidin‐2‐ones was reported by Lam and co‐workers (Table 19).^[75]^ Substrates containing a terminal allene tethered to an α‐ketoamide were studied first, and the optimized reaction conditions involved reacting the substrates with an arylboronic acid (1.5 equiv.), Ni(OAc)_2_ ⋅ 4H_2_O (5 mol %), and (*S*)‐*t*‐Bu‐PHOX (**L4**, 5 mol %) in TFE at 80 °C for 24 h. Products were formed in high yields and enantioselectivities from substrates **104** having phenyl (**105 a**), methyl (**105 b**), *i*‐propyl (**105 c**), or 2‐furyl (**105 d**) substitutents at the ketone. Changing the nitrogen substituent from a *para*‐methoxyphenyl to a benzyl group led to a slight decrease in the enantioselectivity; however, when switching the solvent to MeCN, the product **105 e** was obtained in 99 % ee. Notably, the reaction was successful with a substrate lacking a protecting group on the nitrogen atom, which gave **105 f** in 90 % yield and 99 % ee. Varying the arylboronic acid showed that *para*‐substituents such as acetoxy (**105 g**), chloro (**105 h**), and vinyl (**105 i**) are compatible with the reaction. A variety of 2‐substituted phenylboronic acids containing methyl (**105 j**), fluoro (**105 k**), or amino (**105 l**) groups are also tolerated; however, the latter example led to the product in a moderate yield and low ee. The proposed mechanism is analogous to the one shown in Scheme 2C.

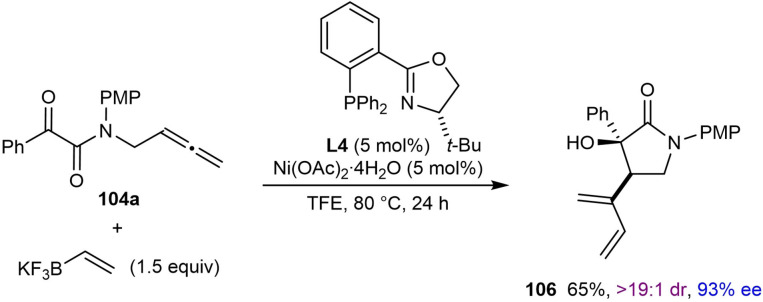




**Table 19:**
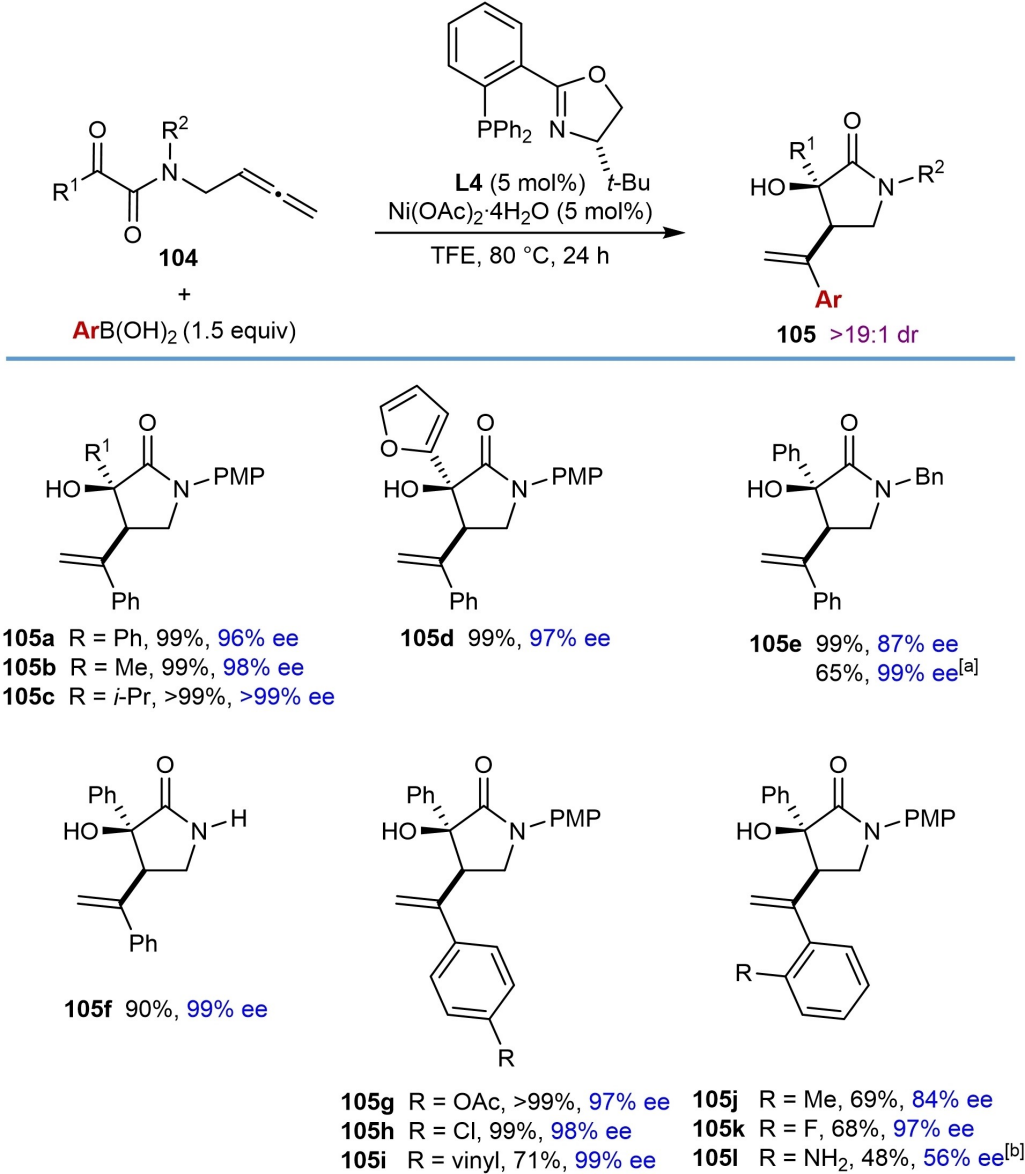
Enantioselective nickel‐catalyzed arylative cyclizations of allene‐tethered ketones to give 3‐hydroxypyrrolidin‐2‐ones.

[a] Conducted in MeCN instead of TFE. [b] Conducted using the pinacol boronate instead of the boronic acid.

Interestingly, the reaction of allene‐tethered α‐ketoamide **104 a** with potassium vinyltrifluoroborate in place of an arylboronic acid was successful in providing pyrrolidin‐2‐one **106** in 65 % yield and 93 % ee [(Eq. (41)].

By replacing the α‐ketoamide with a simple ketone and modifying the tethering group connecting the allene to the electrophile, this process can also be applied to the synthesis of tosyl‐, 4‐methoxyphenyl‐, and 4‐chlorophenyl‐protected pyrrolidines (**108 a**–**108 c**), a piperidine (**108 e**), a cyclopentane (**108 d**), and a cyclohexane (**108 f**) (Table 20). Compared with the synthesis of 3‐hydroxypyrrolidin‐2‐ones (Table 19), the yields and enantioselectivities were more variable and modest in a few cases.

**Table 20:**
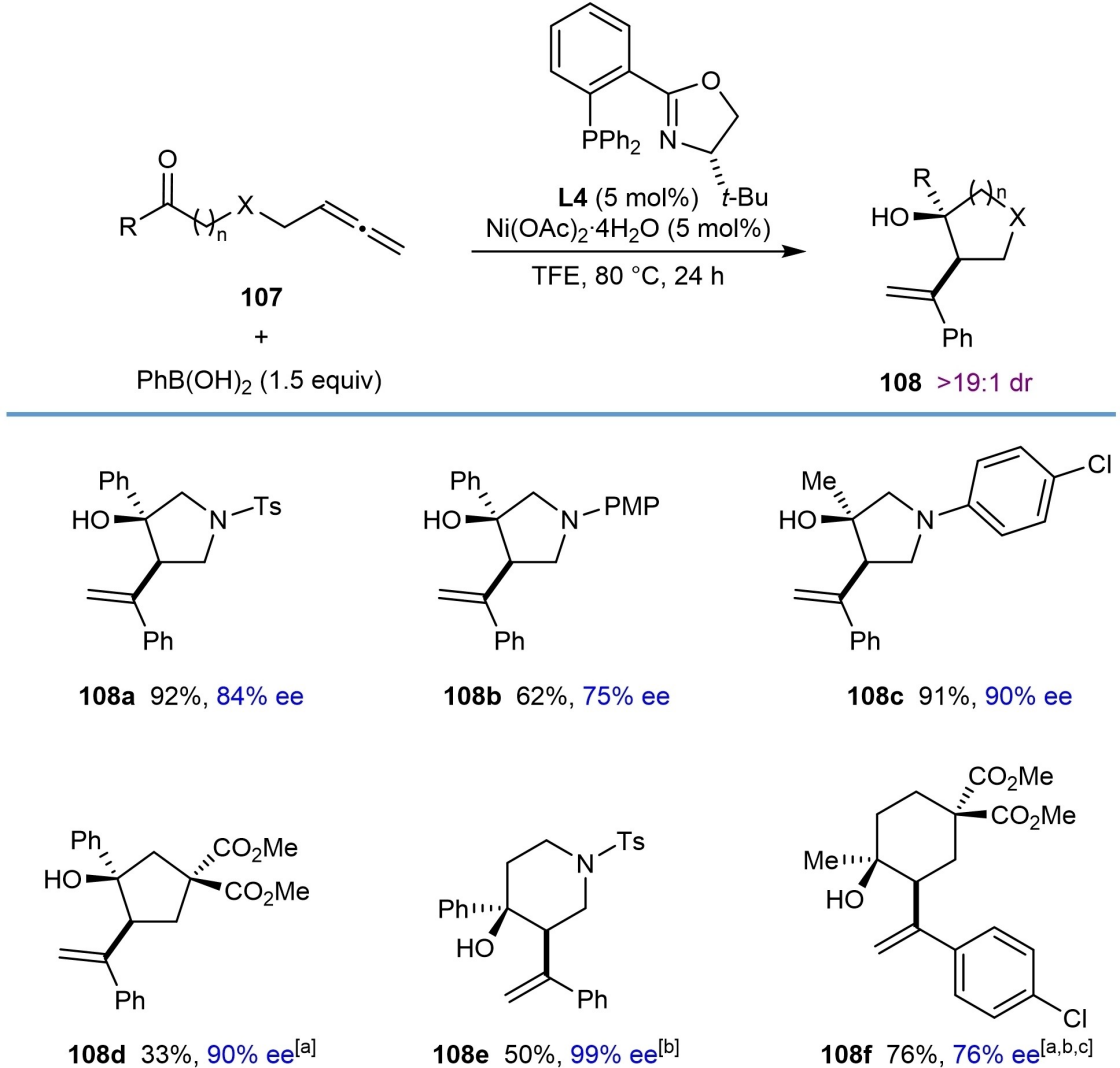
Further exploration of scope of enantioselective nickel‐catalyzed arylative cyclizations of allene‐tethered ketones.

[a] Conducted in MeCN instead of TFE. [b] The reaction time was 48 h. [c] Conducted using 10 mol % each of Ni(OAc)_2_ ⋅ 4H_2_O and **L4**.

## 7 Related Annulation Reactions of 2‐Formyl‐ or 2‐Acylarylboronic Acids with Alkynes or Allenes

This section describes nickel‐catalyzed domino arylation‐cyclization reactions where the electrophile is not tethered to an alkyne or allene, but is instead attached to the 2‐position of the arylboronic acid. Although the connectivity of the reacting components is different, the inclusion of these related annulation reactions is relevant because they proceed via mechanistic steps very similar to those already discussed in the previous sections, involving the cyclization of an alkenyl‐ or allylnickel species onto an electrophile.

In their first report of enantioselective nickel‐catalyzed *anti*‐arylmetallative cyclizations of alkyne‐tethered electrophiles,^[65]^ the Lam group also described one example of the annulation of 1‐phenyl‐1‐butyne with 2‐formylphenylboronic acid using Ni(OAc)_2_ ⋅ 4H_2_O (10 mol %) and (*S*,*S*)‐*t*‐Bu‐FOXAP (**L7**, 10 mol %) in MeCN/2‐MeTHF (3 : 2) at 80 °C for 20 h [(Eq. (42)]. This reaction provided an indenol in 81 % yield and 87 % ee. Gu, Chen, and Xu later developed an enantioselective palladium‐catalyzed version of this reaction.^[112]^


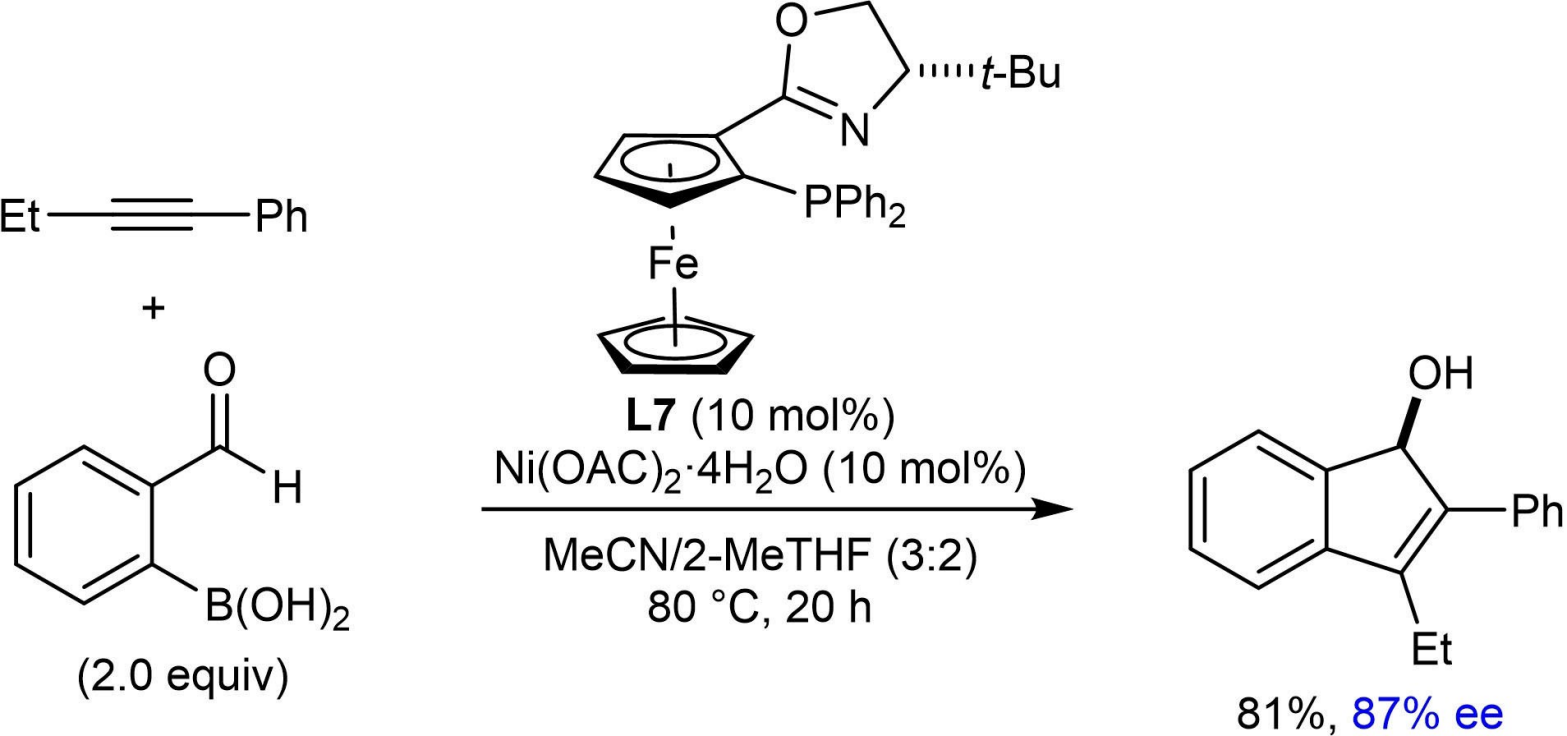




Recently, Kong and co‐workers incorporated the annulation reaction shown in Eq. (42) into more complex domino reactions where three new bonds are formed (Table 21).^[113]^ This process achieves the diastereo‐ and enantioselective synthesis of spirocyclic products via nickel‐catalyzed cascade borrowing hydrogen cyclization. The reactions involve the annulation between 2‐formylphenylboronic acid and 1,6‐enynes **109** in the presence of Ni(OAc)_2_ ⋅ 4H_2_O (10 mol %) and (*S*,*S*)‐*i*‐Pr‐FOXAP (**L8**, 20 mol %) in NMP at 100 °C to give nickel alkoxide **110**. β‐Hydride elimination of **110** gives indene **111** along with a nickel hydride species, which can then undergo migratory insertion into the alkene to give alkylnickel species **112**. Cyclization by 1,4‐addition of the alkylnickel species to the enone provides the nickel enolate **113** that undergoes protonolysis to give the product **114** and regenerate the active nickel catalyst.

**Table 21:**
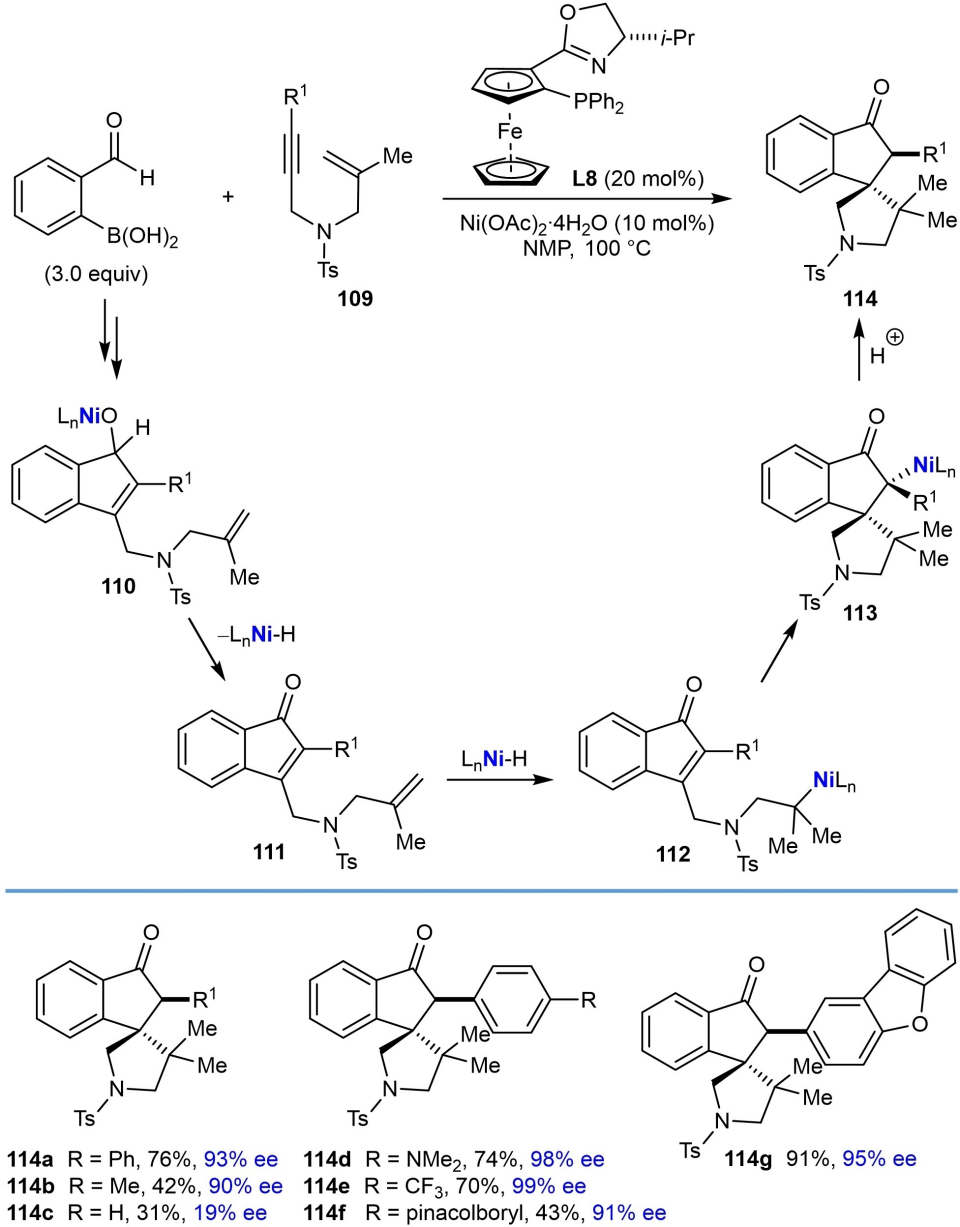
Enantioselective synthesis of spirocycles by nickel‐catalyzed cascade borrowing hydrogen cyclization; 1,6‐enyne scope.

The scope of the reaction with respect to the alkyne substituent was investigated. As well as a simple phenyl group (**114 a**), various substituted phenyl groups containing dimethylamino (**114 d**), trifluoromethyl (**114 e**), or pinacolboronate (**114 f**) groups at the 4‐position are tolerated. A substrate with a dibenzofuran group at the alkyne also reacted well to give **114 g**. In the presence of a methyl alkyne the reaction proceeded to give **114 b** in moderate yield (42 %) but with high enantioselectivity (90 % ee). The reaction of terminal alkyne led to a low yield and poor enantioselectivity (**114 c**, 31 % yield, 19 % ee). Interestingly, the reaction of a substrate **109 h** with a benzyloxymethyl group on the alkyne gave alkene **115**, via β‐alkoxide elimination, in 32 % yield and 98 % ee [Eq. (43)]. This reaction also gave indenol **116** in 30 % yield, where the second cyclization did not occur.

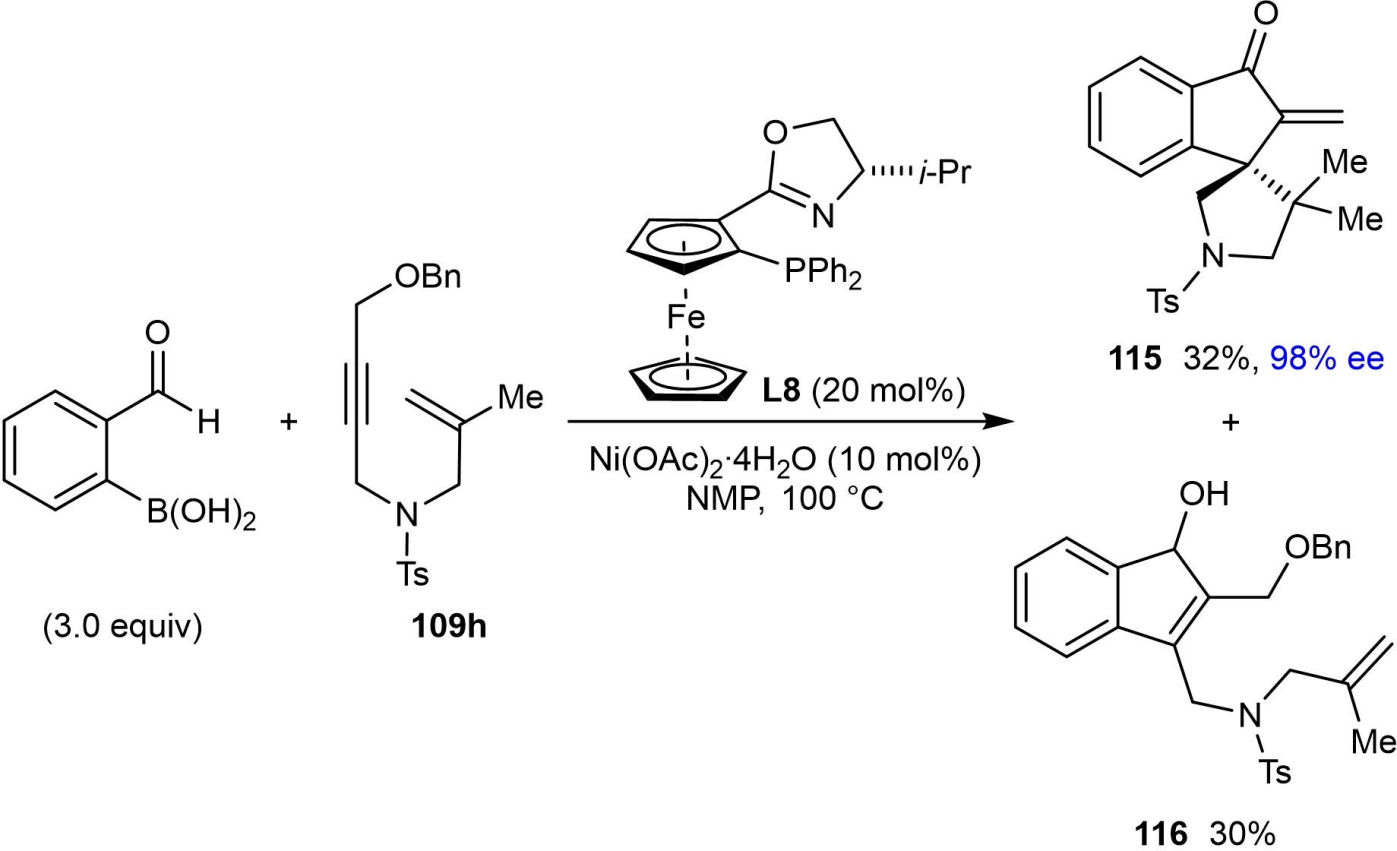




Next, the authors investigated the scope using more highly substituted 2‐formylarylboronic acids in the reaction with electron‐deficient 1,6‐enyne **117 a** (Table 22). Methoxy (**118 b**) or chloro (**118 c**) substitutents on the 2‐formylphenylboronic acid are tolerated, but the use of 5‐[(*tert*‐butyldimethylsilyl)oxy]‐2‐formylphenylboronic acid led to the deprotected phenol **118 d**. (2‐Formylthiophen‐3‐yl)boronic acid also successfully underwent the reaction; however, a low yield and decreased ee was observed (**118 e**, 42 %, 64 % ee). The reaction of 2‐vinylphenylboronic acid with 1,6‐enyne **117 a** was unsuccessful in providing **119** [Eq. 44].

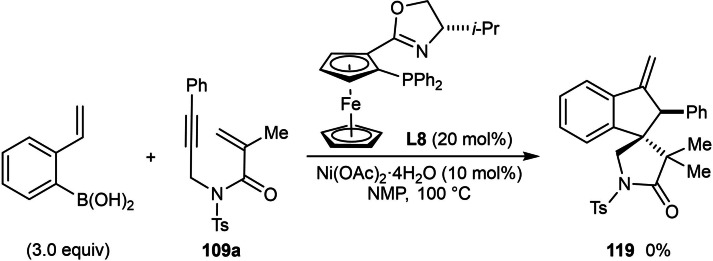




**Table 22:**
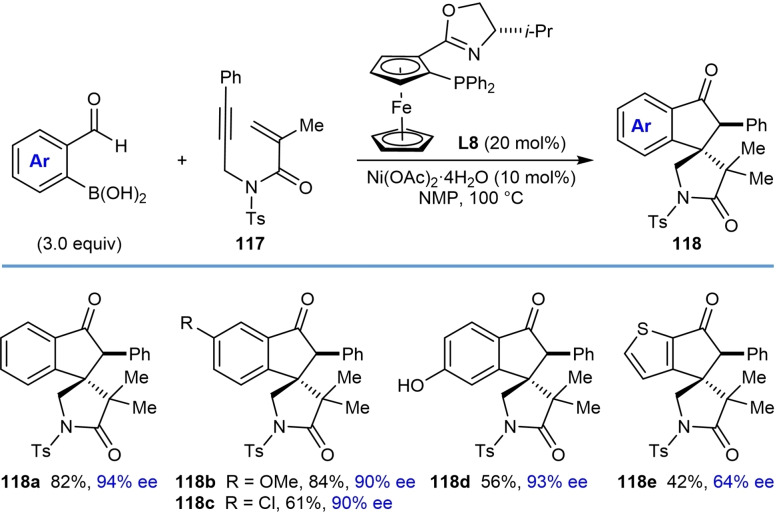
Enantioselective synthesis of spirocycles by nickel‐catalyzed cascade borrowing hydrogen cyclization; 2‐formylarylboronic acid scope.

Finally, the authors investigated the scope of the alkenyl substituent of the 1,6‐enyne **117** in the reaction with 2‐formylphenylboronic acid (Table 23). Substituents such as hydrogen (**120 a**), *n*‐hexyl (**120 b**), ester (**120 c**), and phenyl (**120 d**) are tolerated and provide moderate to good yields of the products in excellent enantiomeric excesses. Also, a 1,6‐enyne **121** with a trisubstituted alkene reacted successfully to give product **122** in 42 % yield and 97 % ee [Eq. 45].

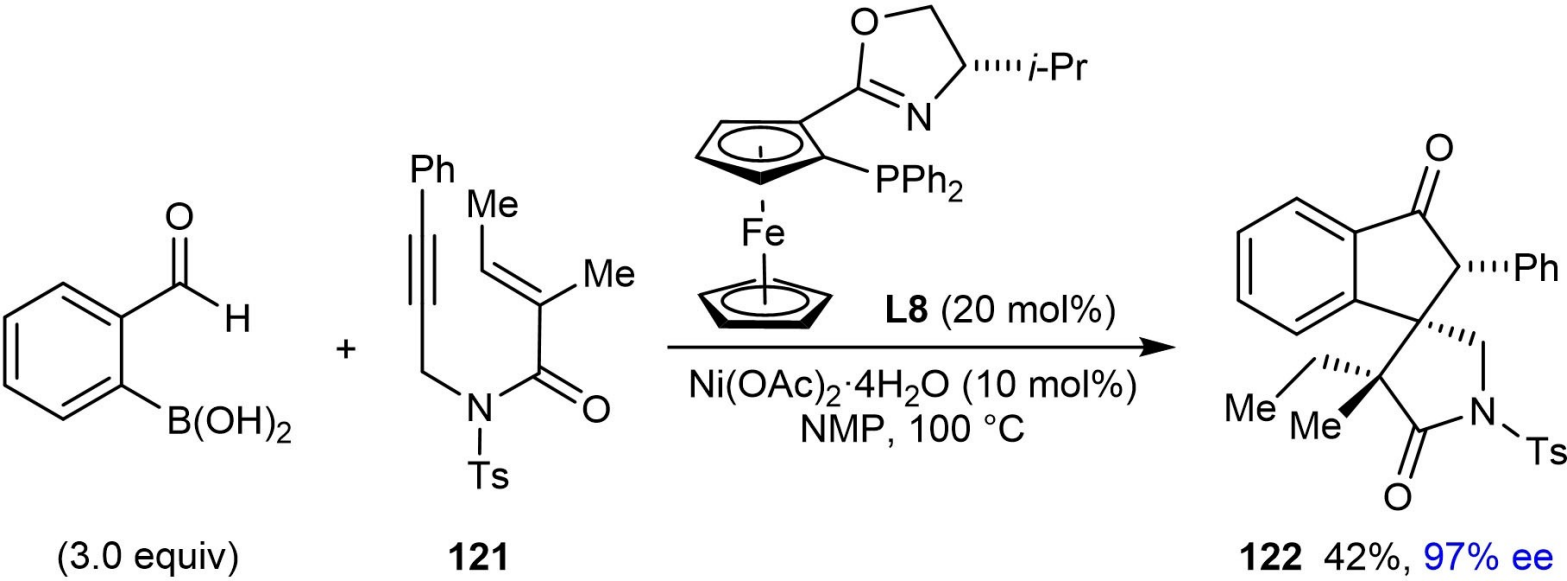




**Table 23:**
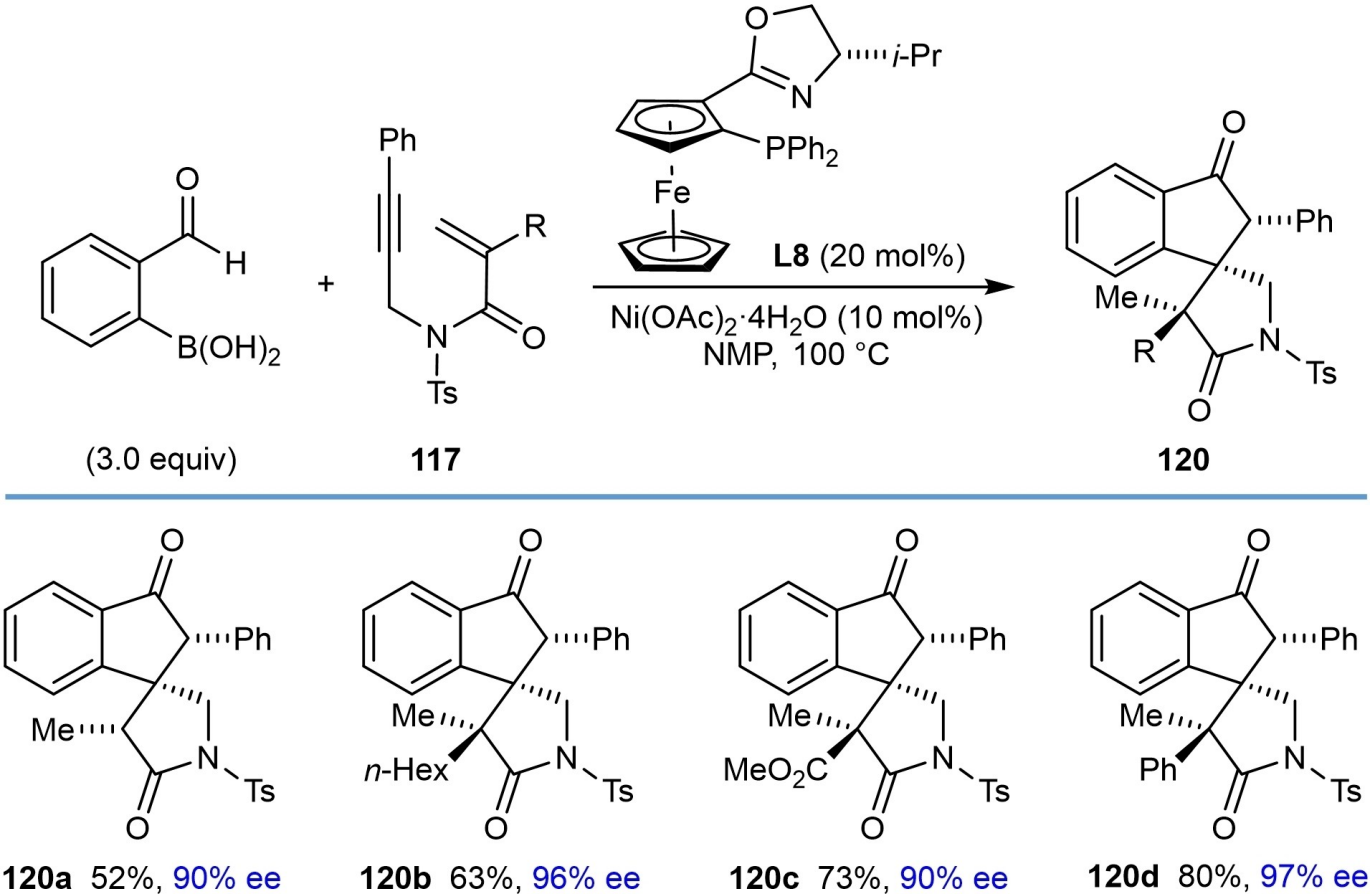
Enantioselective synthesis of spirocycles by nickel‐catalyzed cascade borrowing hydrogen cyclization; scope of alkenyl substituent on the 1,6‐enyne.

Highly diastereoselective nickel‐catalyzed annulation reactions between activated allenes and 2‐formyl‐ or 2‐acetylarylbonic acids to give 3‐methyleneindan‐1‐ols **124** were reported by Lam and co‐workers (Table 24).^[114]^ The optimized reaction conditions were Ni(OAc)_2_ ⋅ 4H_2_O (10 mol %) in MeCN/1,4‐dioxane (3 : 2) at room temperature for 24 h. Using 2‐acetylphenylboronic acid, the scope of the reaction regarding the electron‐withdrawing group on the allene was investigated and benzyl, ethyl, and phenyl esters (**124 a**–**124 c**) are tolerated as well as amide, thioester, and phenyl ketone groups (**124 d**–**124 f**). Non‐carbonyl substituents on the allene, such as phosphonate (**124 g**) or phenylsulfone (**124 h**) are also successful in providing the desired products; however, in the latter example a low yield was observed. 2‐Formylphenylboronic acid reacted successfully with allenes containing a benzyl ester (**124 i**) or diphenylamide (**124 j**). More highly functionalized 2‐formylphenylboronic acids containing chloride or [1,3]‐dioxol‐5‐yl groups also reacted successfully to give products **124 k** and **124 l**, respectively, in modest yields.

**Table 24:**
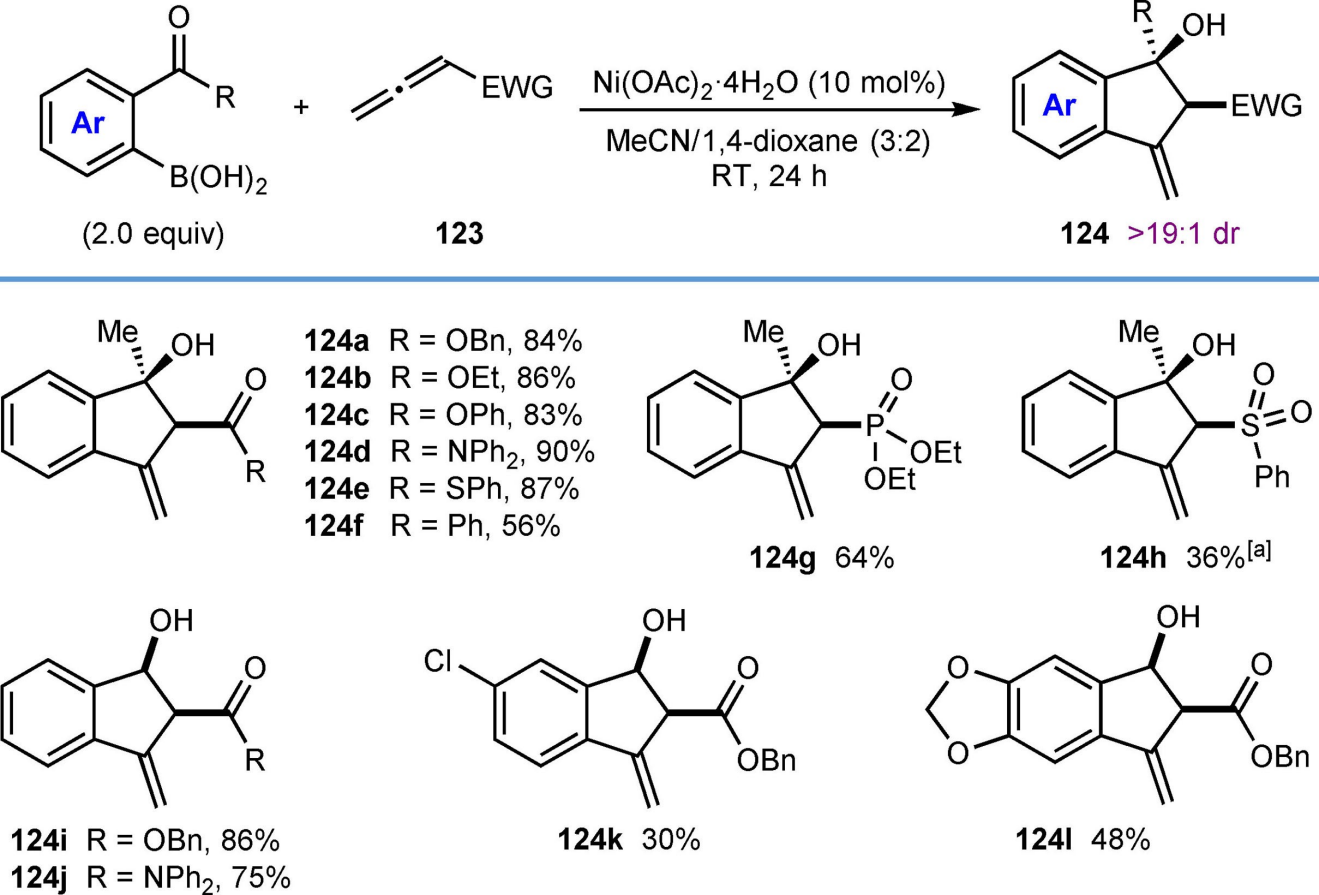
Nickel‐catalyzed annulations between activated allenes and 2‐acetyl‐ or 2‐formylarylboronic acids.

[a] Isolated with an unknown impurity; the yield of **124 h** was determined by ^1^H NMR analysis using 1,3,5‐trimethoxybenzene as an internal standard.

The reaction of 2‐acetylphenylboronic acid with more sterically demanding 1,1‐disubstituted allenes was also investigated (Table 25). Allenyl esters **125** with substituents such as α‐methyl or α‐cyanomethyl were successful in providing the desired products **126 a** and **126 b** in 51 % and 61 % yields, respectively. Also, spirocycle **126 c** was obtained in 71 % yield from 3‐vinylidenedihydrofuran‐2(3*H*)‐one.

**Table 25:**
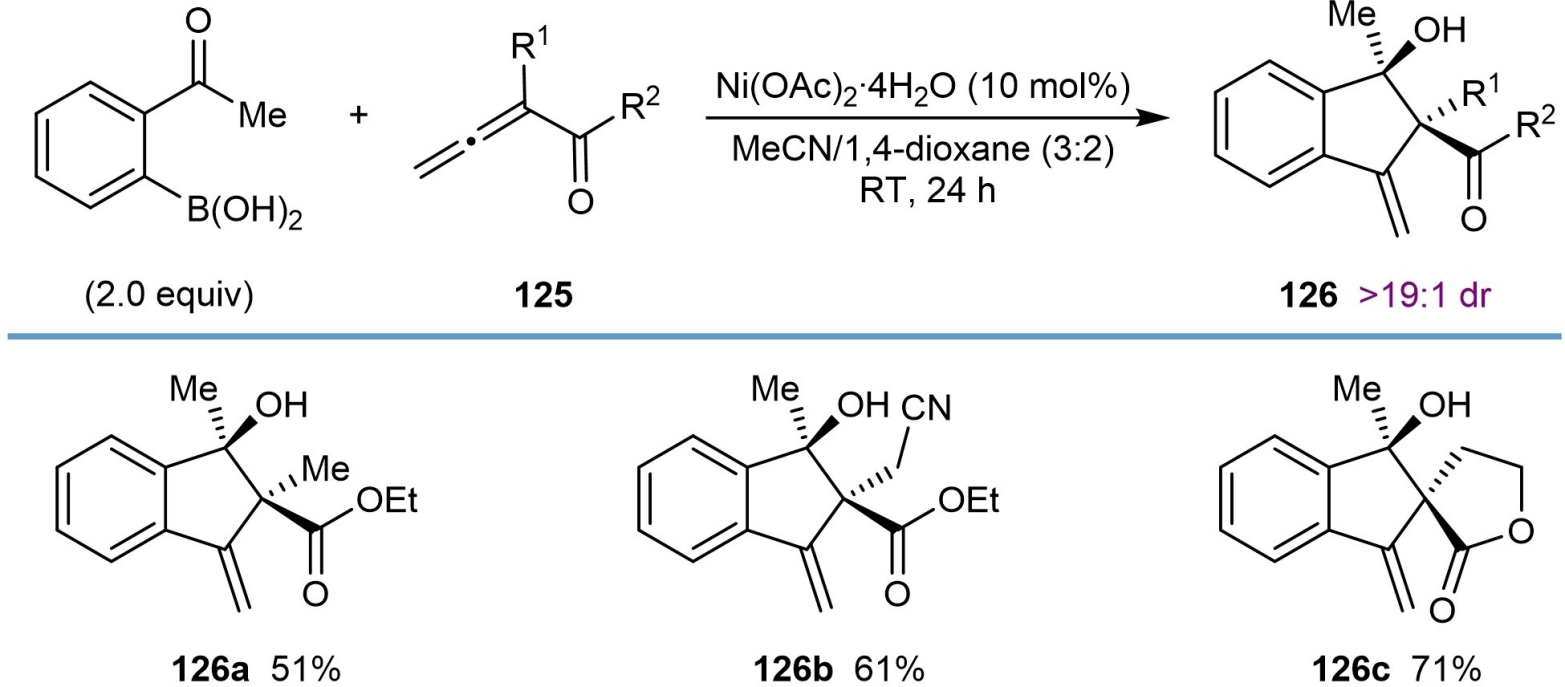
Nickel‐catalyzed annulations between activated allenes and 2‐acetyphenylboronic acid; generation of contiguous quaternary centers.

In addition, the reaction of a trisubstituted allene with 2‐acetylphenylboronic acid gave a product with a fully substituted exocyclic alkene in 31 % yield [Eq. 45].

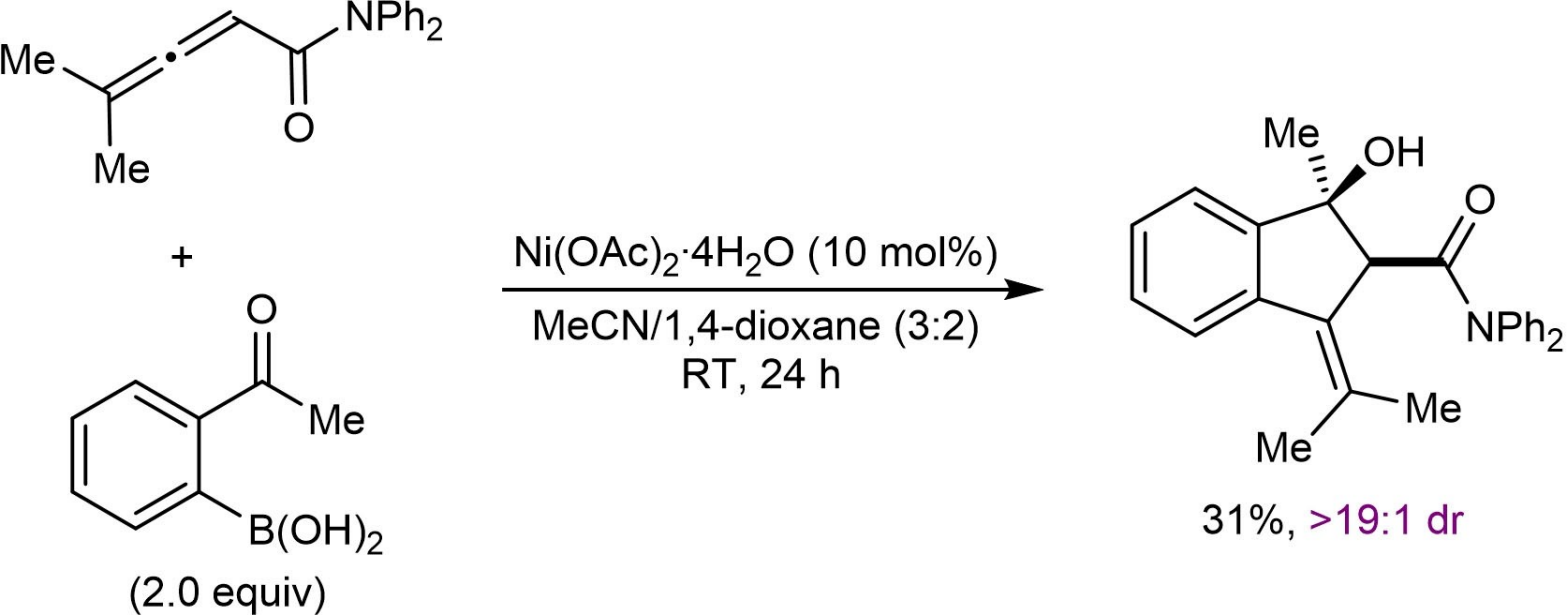




An enantioselective variant of the annulation reaction was also conducted [(Eq. (46)]. Phosphine‐oxazoline ligand **L9** was employed in the presence of Ni(O_2_CCF_3_)_2_ ⋅ 4H_2_O and 1,4‐dioxane to give the enantioenriched product **124 a** in 76 % yield and 74 % ee.

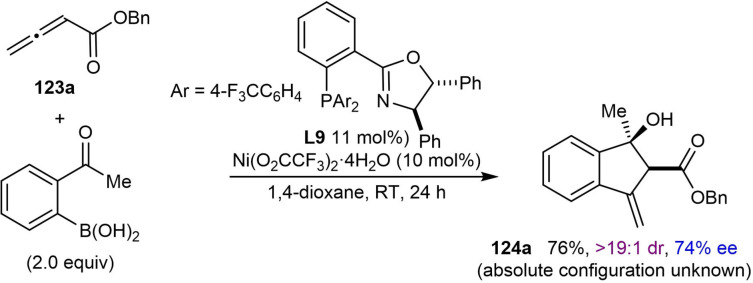




## 8 Summary and Outlook

This Review has summarized the substantial growth in recent years in the application of nickel catalysis to promote arylative cyclizations of alkyne‐ and allene‐tethered electrophiles using arylboron reagents, and in related annulation reactions of 2‐formylarylboronic acids or 2‐acetylphenylboronic acid with alkynes or allenes. Although rhodium and palladium catalysis has featured heavily in these types of reactions in the past, the discovery that nickel opens up new modes of reactivity not readily available to other metals has resulted in an impressive range of new developments and allowing access to a broad range of carbo‐ and heterocyclic products.

For alkyne‐tethered electrophiles, by using electronically dissimilar substituents on the alkyne, the regioselectivity of the migratory insertion of the arylnickel species into the alkyne can be controlled, leading to the selective synthesis of diverse cyclic products containing either exocyclic or endocyclic alkenes. Furthemore, many of the reactions of alkyne‐tethered electrophiles are *anti*‐carbometallative cyclizations that rely upon the reversible *E*/*Z* isomerization of alkenylnickel intermediates, a mode of reactivity that had previously been underexplored. By using allene‐tethered electrophiles, carbo‐ and heterocycles containing an alkenyl group can be obtained. A wide range of electrophiles can be used in nickel‐catalyzed arylative cyclizations, such as cyclic and acyclic ketones, nitriles, allylic phosphates, azides, amides, malonic esters, esters, alkenes, malononitriles, cyclic and acyclic α,β‐unsaturated ketones, α,β‐unsaturated nitriles, and nitroalkenes. Products containing five‐, six‐, or seven‐membered rings have been prepared using this chemistry. Additionally, by using chirally modified catalysts, highly enantioselective reactions have been reported.

The integration of nickel‐catalyzed arylative cyclizations into more complex domino reaction sequences has also recently appeared. Although these processes present greater challenges with respect to chemoselectivity, recent work has demonstrated impressive progress to give complex products with high regio‐, diastereo‐, and enantioselectivities.

However, limitations in this area of nickel catalysis have been identified. In general, increasing the scope of the pronucleophile beyond arylboron reagents has met with limited success, with only a few examples of heteroarylboron or alkenylboron reagents being successfully used. The greater propensity of these reagents to undergo unproductive protodeboronation has been a major challenge, and future methods to overcome this difficulty will have a welcome benefit on increasing the reaction scope. Furthermore, attempted reactions using alkylboronic acids have thus far been unsuccessful, which may stem from difficulties in transmetallation. Possible solutions to successfully engage alkylboron reagents may lie in the generation of alkyl radicals and the formation of open‐shell Ni(I) or Ni(III) species, which may greatly expand the scope of accessible products.

A greater mechanistic understanding of the elementary steps in the nickel‐catalyzed arylative cyclizations (such as the reversible *E*/*Z* isomerization of alkenylnickel species), and what factors influence them, would be advantageous to guide the design of future reactions. Future mechanistic studies are expected to support the continued development of this exciting and rapidly growing area of nickel catalysis.

## Conflict of interest

The authors declare no conflict of interest.

9

## Biographical Information

*Hon Wai Lam received an MChem degree from the University of Oxford in 1998 and a PhD in chemistry from the University of Nottingham in 2001. He conducted postdoctoral studies at Harvard University during 2002 and 2003, and returned to the UK to take up a lectureship at the University of Edinburgh in October 2003, where he was promoted to Reader. In October 2013, he moved to the University of Nottingham as a Professor of Chemistry. Hon's research interests are in the development of new catalytic reactions for organic synthesis, with a focus on transitional metal catalysis and asymmetric synthesis*.

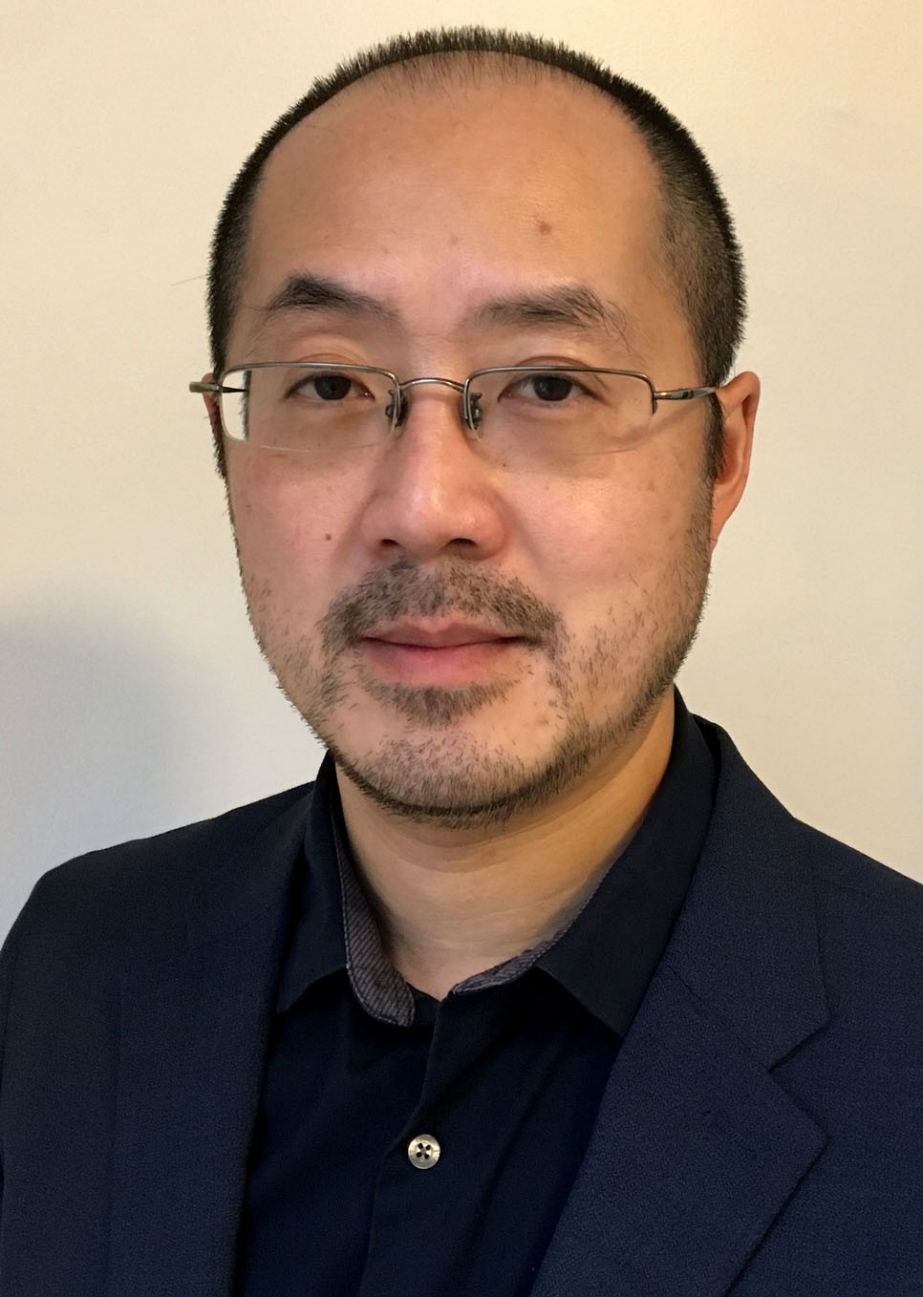

## Biographical Information

*Simone M. Gillbard is currently undertaking her PhD at the University of Nottingham (United Kingdom) under the supervision of Prof. Hon Wai Lam following the completion of her MSc degree in 2018 in the same group. Her research interests are organic synthesis using metal catalysis and she has recently been working on asymmetric nickel‐catalyzed cyclization reactions*.

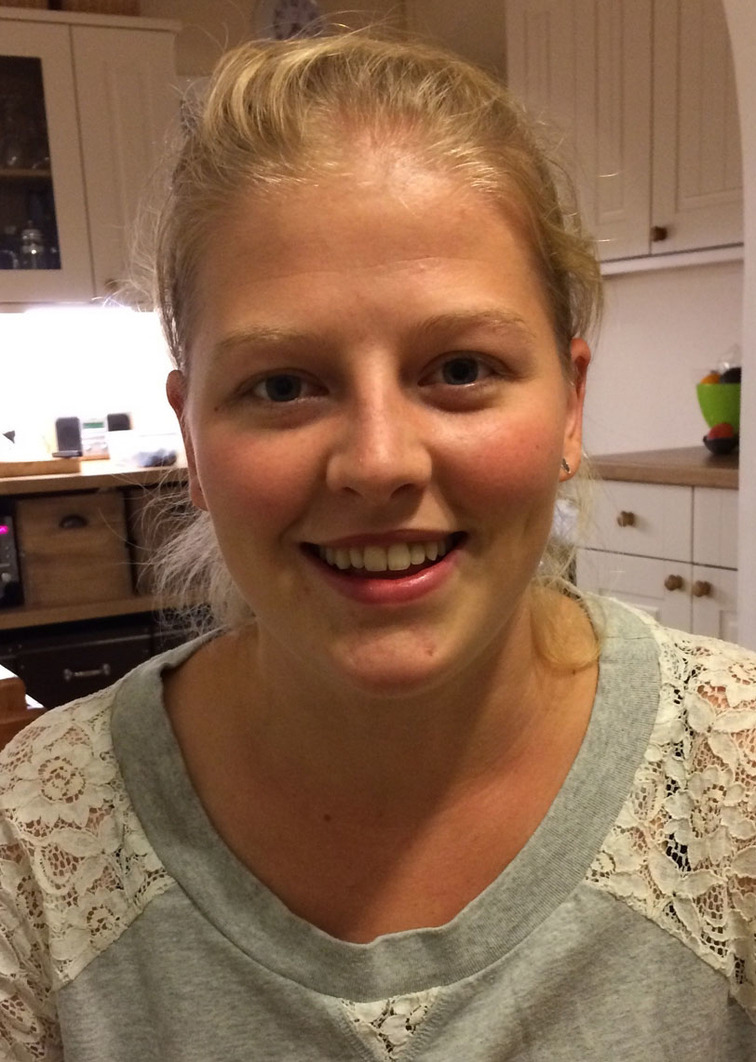

## Supporting information

As a service to our authors and readers, this journal provides supporting information supplied by the authors. Such materials are peer reviewed and may be re‐organized for online delivery, but are not copy‐edited or typeset. Technical support issues arising from supporting information (other than missing files) should be addressed to the authors.

Supporting Information

## Data Availability

Data sharing is not applicable to this article as no new data were created or analyzed in this study.
