# Supplementary material for: Nickel‐Catalyzed Arylative Cyclizations of Alkyne‐ and Allene‐Tethered Electrophiles using Arylboron Reagents
Source: Chemistry. 2022 Jan 27;28(18):e202104230. doi: 10.1002/chem.202104230 (PMC9302687; doi:10.1002/chem.202104230)
Supplement: Supplementary file 1 — Supporting Information [file CHEM-28-0-s001.pdf]

Author Contributions

S.G. Writing – original draft:Lead; Writing – review & editing:Supporting
H.L. Writing – original draft:Supporting; Writing – review & editing:Lead
